# Metal-Free Direct Electrochemical Deoxygenation of Benzylic Alcohols

**DOI:** 10.1021/acssuschemeng.5c08410

**Published:** 2025-12-02

**Authors:** Alex G. Edmonds, Darren A. Walsh, Kristaps Ermanis, James D. Cuthbertson

**Affiliations:** † The GlaxoSmithKline Carbon Neutral Laboratories for Sustainable Chemistry, 6123 University of Nottingham, Jubilee Campus, Triumph Road, Nottingham NG7 2TU, U.K.; ‡ School of Chemistry, University of Nottingham, University Park, Nottingham NG7 2RD, U.K.

**Keywords:** electrosynthesis, deoxygenation, benzylic alcohol, phosphoranyl radical, C−O bond activation

## Abstract

An operationally simple electrochemical deoxygenation of benzylic alcohols is reported. The one-step, one-pot process is metal-free and is conducted at room temperature in an undivided cell, offering a more sustainable and step-economical alternative to conventional derivatization–cleavage deoxygenation sequences. The applicability of the reaction is demonstrated using an electronically and sterically diverse set of primary, secondary, and tertiary benzylic alcohols; excellent functional-group tolerance is exhibited, permitting the presence of esters, amides, and even unactivated alcohols in the substrate scope. Based on preliminary mechanistic investigations using chemical probes, electroanalytical techniques, and computational studies, the reaction is postulated to proceed via the reaction of an alcohol with a phosphine radical cation that is formed in situ.

## Graphical abstract

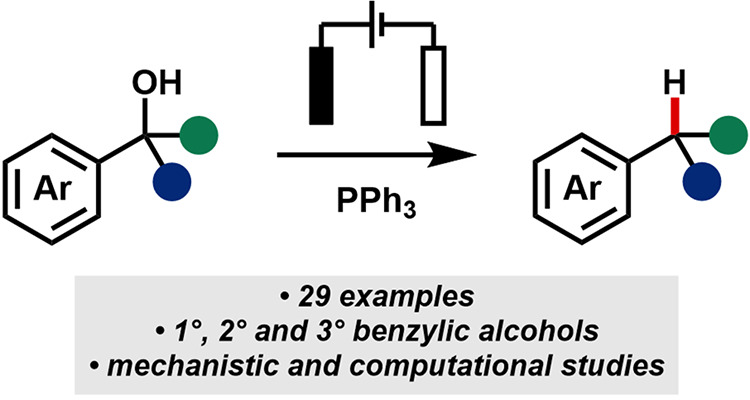

## Introduction

The alcohol functional group, a hydroxy group bound to a saturated carbon atom, is encountered in a diverse range of molecules, including natural products, pharmaceuticals, agrochemicals, and materials.^1^ As a result, a multitude of transformations of the hydroxy group have been developed.^2,3^ However, general methods that enable the deoxygenative functionalization of alcohols, without the requirement for preactivation, remain limited due to the high strength of the C–O bond (94 kcal mol^–1^ C–O EtOH),^4^ the fact that hydroxide is a poor leaving group, and the highly negative potentials required to reduce the C–OH bond.^5^ Furthermore, the direct deoxygenation of alcohols to give the corresponding alkanes still presents a challenge; while a few methods have been reported, they generally require harsh conditions, and are often limited to particular classes of substrate or are limited in their functional-group tolerance.^6–15^ As a result, conventional methods for the deoxygenation of alcohols usually involve an initial derivatization step to give access to more readily cleaved functionalities such as halides, xanthates, oxalates, and tosylates.^9^

Recently, Doyle and co-workers developed a phosphorus-mediated deoxygenation of benzylic alcohols and carboxylic acids enabled through the fragmentation of a photochemically generated phosphoranyl radical (Scheme 1A).^16^ Subsequently, a range of photochemical deoxygenation reactions proceeding via phosphoranyl radical intermediates have been reported.^17,18^ However, despite the value of these processes, they typically rely on the use of precious metal photocatalysts. As a result, there remains a need for deoxygenation reactions that proceed under mild conditions without the requirement for such catalysts.

**Scheme 1:**
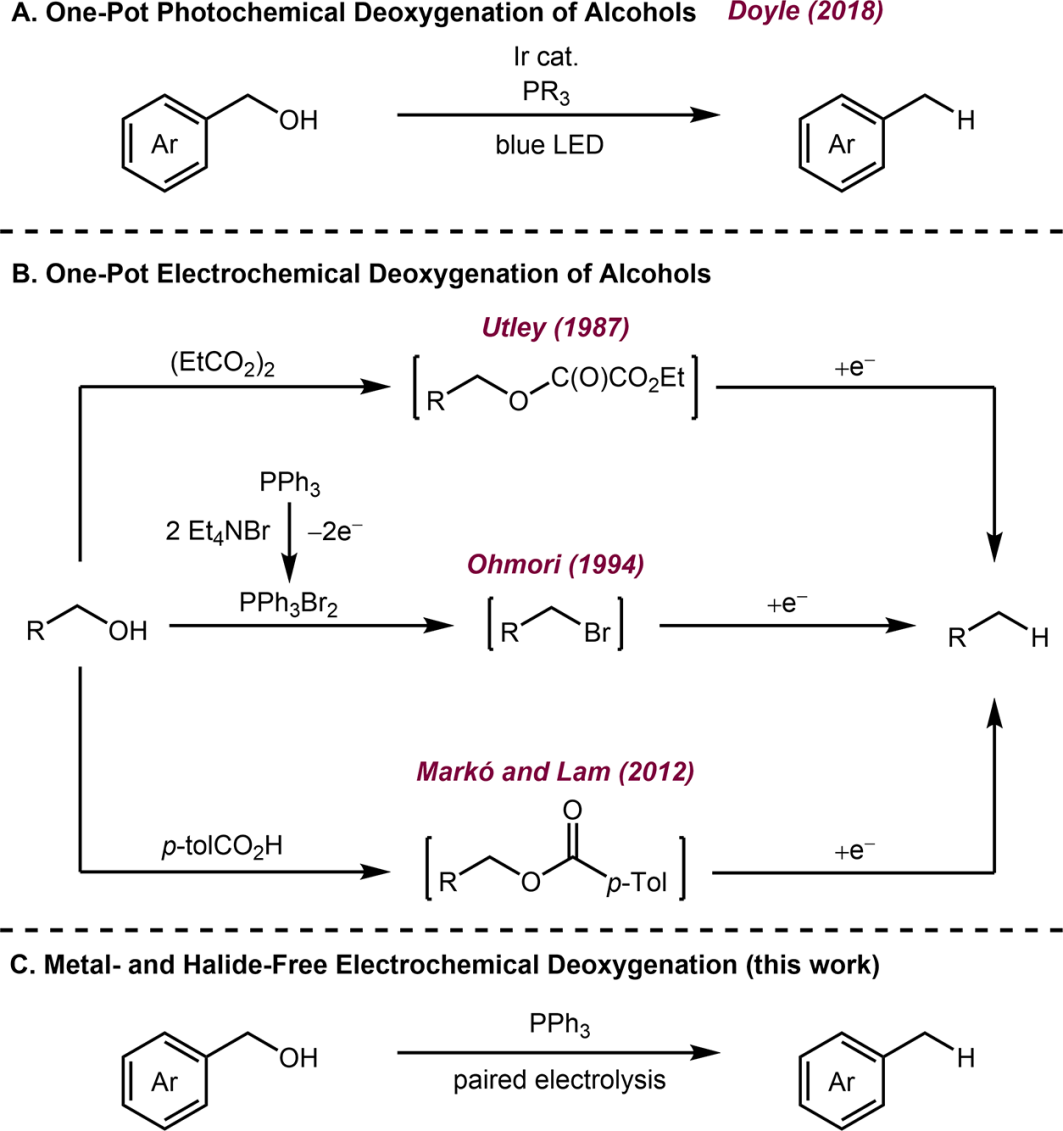
Strategies for the Deoxygenation of Alcohols Using (A) One-pot Photochemical Deoxygenation, (B) One-pot Electrochemical Deoxygenation, and (C) Metal and Halide-Free Deoxygenation

Electrochemical methods can potentially offer a more sustainable alternative to conventional synthetic approaches,^19^ enabling the activation of a range of functional groups under mild conditions.^20,21^ However, while a variety of electrochemical deoxygenations have been developed for alcohol derivatives such as phosphinates,^22–24^ sulfonates,^25–27^ esters,^28,29^ and ethers,^30,31^ few electrochemical methods for the deoxygenation of alcohols exist. Known electrochemical methods for the one-pot deoxygenation of alcohols typically rely on in situ generation of a redox-active intermediate, followed by reductive cleavage (Scheme 1B). In 1987, Utley and co-workers developed a one-pot electrochemical transesterification-reduction sequence to reduce benzyl alcohols via an electrogenerated alkyl oxalate intermediate (Scheme 1B, top).^29^ Subsequently, Ohmori et al. developed an electrochemical deoxygenation proceeding via an Appel-type in situ bromination of alcohols, followed by electroreduction of the resulting alkyl bromide (Scheme 1B, middle).^32^ In 2012, Markó and Lam disclosed an electrochemical deoxygenation of primary alcohols, which proceeds by reduction of toluate esters generated in situ (Scheme 1B, bottom).^33^ In addition to these methods, specific protocols for the direct electrochemical reduction of simple (*C* ≤ 4) allylic^34–36^ and benzylic alcohols have been reported,^5,37^ but general methods enabling the direct deoxygenation of alcohols remain elusive.

Given the propensity of phosphoranyl radicals generated in a photoredox-mediated process to fragment to give the corresponding alkyl radical,^18^ we questioned whether a similar transformation could be achieved electrochemically, thereby effecting a direct deoxygenation without the requirement for a precious metal photocatalyst (Scheme 1C). While, to the best of our knowledge, this metal- and halide-free electrochemical strategy has not been exploited for the deoxygenation of alcohols, during the course of our studies, the groups of Wang and Tian reported a mechanistically similar strategy for the deoxygenation of ketones.^38^ Furthermore, the same groups recently used electrochemically generated phosphoranyl radicals in the dehydroxylative arylation of alcohols and aldehydes/ketones.^39,40^

We report here the results of our studies on the metal-free electrochemical deoxygenation of alcohols enabled by the reaction of an alcohol with an electrochemically generated phosphine radical cation. The reaction, which proceeds under mild conditions, tolerates diverse functionality, including esters, nitriles, boronate esters, and halides, giving access to a range of substituted toluene derivatives. By avoiding prior derivatization of the alcohol substrates, step economy is improved, and the handling and isolation of potentially hazardous intermediates is obviated.

## Experimental Section

All air-sensitive reactions were carried out under an atmosphere of Ar, using an oven-dried apparatus. All commercially available reagents were used as received, while tetrabutylammonium hexafluorophosphate (TBAPF_6_) was purified by recrystallization from boiling ethanol. Thin-layer chromatography (TLC) was performed on Merck 60 F254 0.2 mm precoated plates. Compounds were visualized by exposure to UV light or by dipping the plates into solutions of potassium permanganate, followed by gentle heating. Flash column chromatography was carried out using silica gel (Merck 60 Å particle size 40–63 μm). Melting points were recorded on a Stuart SMP20 melting point apparatus. Infrared (IR) spectra were recorded on a Bruker Platinum Alpha FTIR spectrometer on the neat compound by using the attenuated total reflectance technique.

NMR spectra were acquired on Bruker Ascend 400 and Ascend 500 spectrometers. ^1^H and ^13^C NMR spectra were referenced to external tetramethylsilane via the residual protonated solvent (^1^H) or the solvent itself (^13^C). For CDCl_3_, the shifts were referenced to 7.26 ppm for ^1^H NMR spectroscopy and 77.16 ppm for ^13^C NMR spectroscopy. When using deuterated dimethyl sulfoxide (DMSO-*d*_6_), the shifts were referenced to 2.50 ppm for ^1^H NMR spectroscopy and 39.52 ppm for ^13^C NMR spectroscopy. ^19^F and ^31^P NMR spectra were referenced through the solvent lock (^2^H) signal according to the IUPAC-recommended secondary referencing method, following Bruker protocols. Coupling constants (*J*) are quoted to the nearest 0.1 Hz. Signals are reported as singlet (s), doublet (d), triplet (t), quartet (q), pentet (p), heptet (h), multiplet (m), broad (br), apparent (app), or combinations thereof. All chemical shifts are reported in parts per million (ppm). For ^13^C NMR spectroscopy, peak assignments were made using the DEPT sequence with secondary pulses at 90 and 135°. Yields determined by ^1^H NMR spectroscopic analysis were calculated from the resonance integrals of an internal standard (1,3-benzodioxole or 1,3,5-trimethoxybenzene) in relation to the resonance integral(s) of the target compound. Conversions determined by ^1^H NMR spectroscopic analysis were calculated from the resonance integrals of an internal standard (1,3-benzodioxole or 1,3,5-trimethoxybenzene) in relation to the resonance integral(s) of the starting material.

Gas chromatography-mass spectrometry (GC-MS) was performed using a Thermo Scientific Trace 1300 Gas Chromatograph equipped with a Thermo Trace Gold TG17-MS column and a Thermo Scientific ISQ LT Single Quadrupole Mass Spectrometer. High-resolution mass spectra were recorded using a Bruker MicrOTOF instrument using electrospray ionization (ESI) techniques. Electrolyses were carried out using a homemade cell consisting of a two-necked tube, a polytetrafluoroethylene (PTFE) stopper, and stainless-steel electrode clamps. A TENMA 72-10480 bench power supply was used to supply constant currents. Cyclic voltammetry studies were performed using 0.1 M TBAPF_6_ in anhydrous MeCN as the electrolyte solution. The working electrode was a 3 mm diameter glassy carbon disk (Part No. CHI104, CH Instruments, Austin, TX). The counter electrode was a Pt wire (CH Instruments Part No. CHI115), and the reference electrode was Ag/AgNO_3_ (Ag wire in 10 mM AgNO_3_, 0.1 M TBAPF_6_ in anhydrous MeCN) (CH Instruments Part No. CHI112). Measurements were recorded using CHI1140c and CHI600E potentiostats.

## Results and Discussion

We began by attempting the deoxygenation of alcohol **1a**, using conditions reported by Ohmori and co-workers.^32^ Using the optimized literature conditions, alcohol **1a** underwent the desired deoxygenation to give toluene derivative **2a** in 93% yield (Table 1, entry 1). In the Ohmori protocol, the reaction is believed to proceed by formation of the corresponding benzyl bromide, which then undergoes cathodic reduction to afford the product (vide supra).^41^ Interestingly, when TBAPF_6_ was used as the electrolyte, the deoxygenation product **2a** was formed in excellent yield, demonstrating that the alcohol could be reduced without the requirement for in situ conversion into the corresponding bromide (Table 1, entry 2).

**Table 1:**
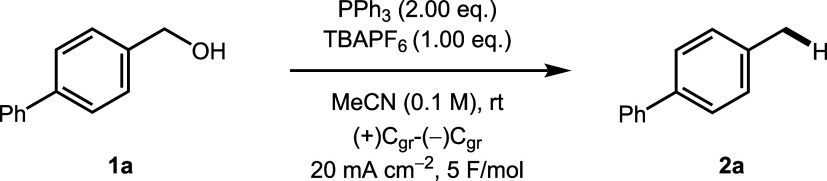
Optimization of the Electrochemical Deoxygenation^a^

entry	deviation from optimized conditions	yield (%)^b^
1	TBABr instead of TBAPF_6_ (Ohmori conditions)^32^	93
2	none	98
3	TBABF_4_ instead of TBAPF_6_	91
4	TBAClO_4_ instead of TBAPF_6_	77
5	5 mA cm^–2^ (13.4 h)	97
6	30 mA cm^–2^ (2.2 h)	94
7	4 F mol^–1^ (2.6 h)	92
8	PPh_3_ (1.50 equiv)	76
9	P(OPh)_3_ instead of PPh_3_	0
10	air atmosphere	68
11	with water (10 equiv)	47
12	no electricity	0
13	no phosphine	21

a Reactions were carried out on a 1.0 mmol scale in MeCN ([**1a**]_0_ = 0.10 M).

b Yields were determined by ^1^H NMR spectroscopy using 1,3-benzodioxole as the internal standard.

The use of alternative electrolytes in this halide-free deoxygenation protocol led to slightly reduced yields (Table 1, entries 3 and 4). Decreasing the current density from 20 to 5 mA cm^–2^ resulted in a comparable yield, but the reaction time increased 4-fold (Table 1, entry 5). Increasing the current density to 30 mA cm^–2^ resulted in a significantly shorter reaction time but led to a marginal decrease in the yield (Table 1, entry 6). Broadly accessible constant current conditions were used, comprising a simple homemade undivided cell (see the SI for details) and an inexpensive bench power supply. A constant potential approach was avoided, which would require a three-electrode cell and potentiostat, significantly increasing the cost and complexity of carrying out this transformation. Reducing the amount of charge passed from 5 to 4 F mol^–1^ also resulted in a slightly decreased yield (Table 1, entry 7). Decreasing the amount of triphenylphosphine from 2.00 to 1.50 equiv resulted in a lower yield of the product, suggesting that the second equivalent may be implicated in the reaction mechanism (vide infra) (Table 1, entry 8). Alternatively, excess phosphine in the reaction mixture may serve to protect the product from further oxidation, as the oxidation potential for triphenylphosphine (PPh_3_ *E*_ox_ = 1.06 V vs saturated calomel electrode (SCE))^42^ is typically lower than that of toluene derivatives (toluene *E*_ox_ = 2.36 V vs SCE).^43^

To form a water-soluble byproduct, the deoxygenation was attempted using triphenylphosphite instead of triphenylphosphine; while the starting material was fully consumed in the reaction, no deoxygenation product was detected (Table 1, entry 9). The optimized deoxygenation protocol could also be run in an air atmosphere, giving the product in a lower, yet synthetically useful, 68% yield (Table 1, entry 10). Furthermore, the product was also formed when water (10 equiv) was introduced (Table 1, entry 11) but the yield was lower, presumably due to a competing reaction between water and the phosphine radical cation.^44^ A control reaction demonstrated that the passage of current was necessary for the reaction to proceed (Table 1, entry 12). Finally, in the absence of phosphine, the deoxygenation product was formed in a 21% yield (Table 1, entry 13). While initially surprising, this result can be attributed to a direct electrochemical reduction, consistent with that recently reported by Lundberg and co-workers.^37^ The *E*-factor for the deoxygenation of **1a** under the optimized conditions was 497 (*E*-factor = 58.1 when excluding chromatographic purification), comparable with the same transformation under the conditions reported by Doyle et al.^16^ (*E*-factor = 500 or 56.4 when excluding chromatographic purification) (see the SI for details).

With the optimized conditions in hand, we explored the scope of the electrochemical deoxygenation reaction. A range of primary benzylic alcohols could be deoxygenated with comparable efficiency to previously reported photochemical methods (Table 2).^16^ The benzyl alcohol derivative **1a** used in optimization studies gave the deoxygenated product **2a** in 80% isolated yield when carried out on a 1.00 mmol scale. Substrates bearing an electron-withdrawing group at the *para*-position gave the corresponding products in good yields (**2b** and **2c**). A naphthyl alcohol was also deoxygenated under the standard conditions, giving product **2d** in 54% yield. Electron-donating *para*-substituents, including methoxy **2e**, BocNH **2f**, and *tert*-butyl **2g**, were also tolerated, but yields were slightly lower than when the electron-deficient substrates were used. In the case of the *tert*-butyl derivative **2g**, the same product could also be obtained from 4-*tert*-butylbenzyl mercaptan (70% yield determined by ^1^H NMR spectroscopy—see SI for details), demonstrating the use of the chemistry for the cleavage of C–S bonds. Halogen substituents could also be present; while *para*-fluoro benzyl alcohol **1h** underwent deoxygenation under the standard conditions, it was necessary to run reactions in dichloromethane (DCM) when chloro, bromo, or trifluoromethyl substituents were present, to avoid undesired reduction of the C–X bonds (**2i**–**2k**). Of note, a B­(Pin) substituent was tolerated, providing a useful handle that could facilitate the derivatization of the scaffold (**2l**). Unfortunately, nitro and aldehyde groups were incompatible with the reaction; in both cases, consumption of the starting material was observed, and a complex mixture of products was formed (**2m** and **2n**). Benzyl alcohols bearing substituents at the *meta*-position also worked, giving the deoxygenated products in moderate yields (**2o** and **2p**). While an amide-containing substrate **1q** could be deoxygenated, attempts to deoxygenate a sulfonamide substrate **1r** led to a complex mixture of unidentified products. Finally, a substrate bearing an *ortho*-methyl substituent **1s** also underwent deoxygenation, giving the product **2s** in 75% yield.

**Table 2:**
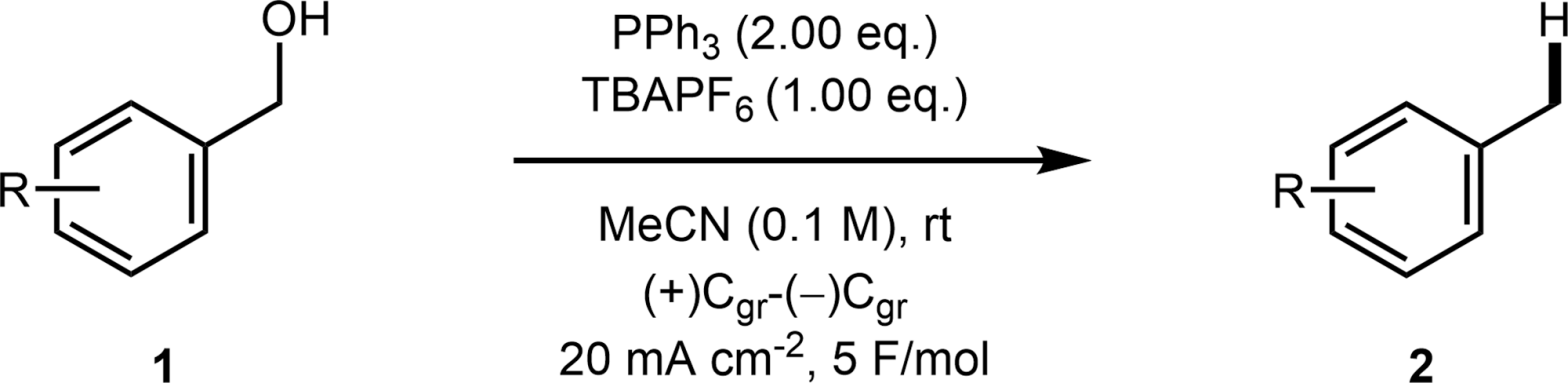

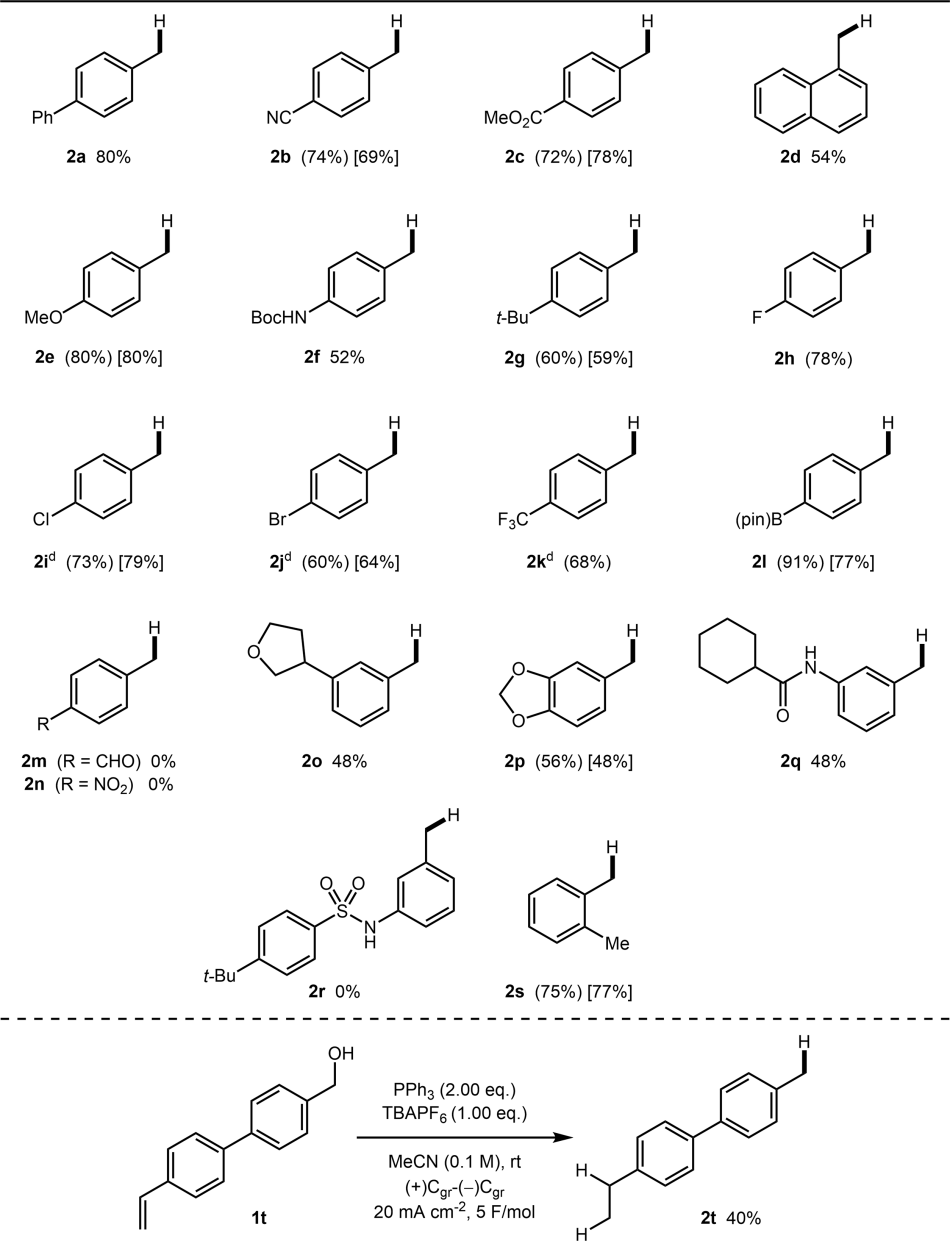
Deoxygenation of Primary Benzylic Alcohols^a^,^b^,^c^,^d^

a Reactions were carried out on a 1.0 mmol scale in MeCN ([**1**]_0_ = 0.10 M).

b Yields refer to material isolated after purification by flash column chromatography.

c Yields in parentheses were determined by ^1^H NMR spectroscopy using 1,3,5-trimethoxybenzene as the internal standard. Yields in brackets were determined by GC-MS analysis using dodecane or tetradecane as the internal standard (see the SI for details).

d Reaction run in DCM.

When styrene derivative **1t** was subjected to the optimized conditions, deoxygenation of the alcohol occurred, along with reduction of the alkene, to give the saturated product **2t** in 40% yield (Table 2). Reaction of phosphine radical cations with alkenes to give the corresponding alkylphosphonium salts is precedented, and such species are reduced under our reaction conditions (see the SI for details).^45–47^ Alternatively, a mechanism similar to the one recently reported by Studer and co-workers could have occurred if traces of water were present.^48^

We next explored the application of the chemistry to more hindered alcohols (Table 3). Secondary benzylic alcohols were tolerated under the optimized conditions (**4a**–**4d**). An α-cyclopropyl group remained intact despite a proposed radical mechanism (**4e**). While surprising, the relief of ring strain in similar systems is known to be insufficient to offset the instability caused by transferring the radical from a benzylic to a primary aliphatic position.^49^ As a result, the cyclopropane form predominates, and a radical intermediate cannot therefore be ruled out based on this observation alone.

**Table 3:**
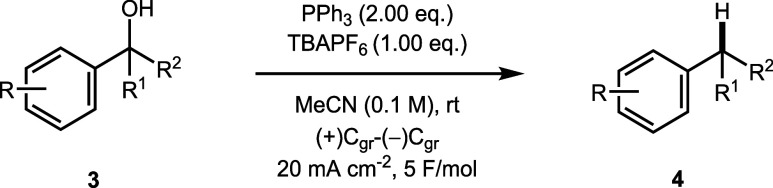

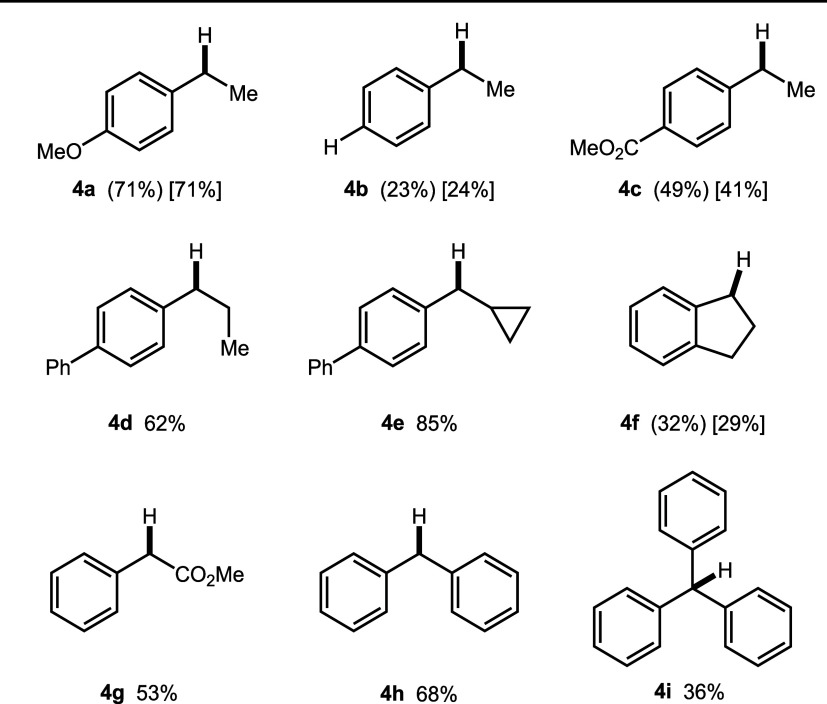
Deoxygenation of Secondary and Tertiary Benzylic Alcohols^a^,^b^,^c^

a Reactions were carried out on a 1.0 mmol scale in MeCN ([**3**]_0_ = 0.10 M).

b Yields refer to material isolated after purification by flash column chromatography.

c Yields in parentheses were determined by ^1^H NMR spectroscopy using 1,3,5-trimethoxybenzene as the internal standard. Yields in brackets were determined by GC-MS analysis using dodecane or tetradecane as the internal standard (see SI for details).

The chemistry was not limited to acyclic alcohols; 1-indanol (**3f**) underwent deoxygenation without significant (<5%) formation of the elimination product. An α-hydroxy ester **3g** was also a suitable substrate and, in this case, the product **4g** was isolated in 53% yield. Typically, deoxygenation of similar alcohols requires harsh conditions or electron-donating substituents on the aromatic ring.^11^ Finally, alcohols adjacent to multiple phenyl rings also underwent deoxygenation (**4h** and **4i**). Notably, the hindered tertiary alcohol triphenylmethanol (**3i**) gave the product triphenylmethane (**4i**) in 36% isolated yield, despite the increased steric hindrance adjacent to the hydroxy group.

We next applied the deoxygenation chemistry to simple aliphatic alcohols. When dodecanol (**5**) was used as the substrate, most of the starting material (>95% as determined by ^1^H NMR spectroscopy) was present at the end of the reaction and no dodecane (**6**) was detected by NMR spectroscopy or GC-MS analysis (Scheme 2a). Furthermore, the predominant species observed by ^31^P NMR spectroscopy at the end of the reaction was triphenylphosphine, providing further evidence to suggest that deoxygenation had not occurred. Consequently, when substrates containing both benzylic and aliphatic alcohols (**7a** and **7b**) were used in the reaction, selective deoxygenation of the benzylic alcohol occurred, giving products **8a** and **8b** in 51 and 48% yield, respectively (Scheme 2b). When 4-phenylbutan-1,4-diol (**9**) was subjected to the deoxygenation protocol, expected product **10** was obtained alongside tetrahydrofuran (THF) **11** (Scheme 2c). Based on previous studies by Ohmori and co-workers,^50,51^ we postulate that this is formed by in situ generation of an alkoxyphosphonium salt (vide infra), which subsequently cyclized to form THF **11**. The formation of THF **11** rules out the possibility of a direct cathodic reduction mechanism; an anionic intermediate resulting from a direct cathodic reduction would not undergo intramolecular substitution. Furthermore, when the expected product **10** was resubjected to the reaction conditions, unreacted starting material **10** was recovered and no evidence of THF **11** was observed, suggesting that a benzylic oxidation mechanism was not operative (see the SI for details).

**Scheme 2:**
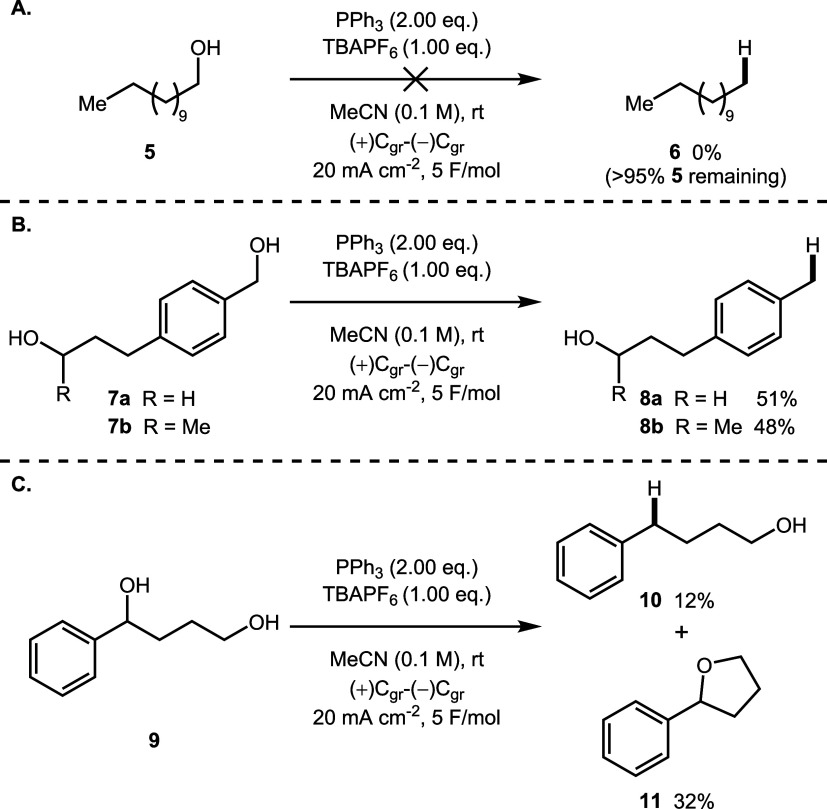
Deoxygenation Reactions Involving Aliphatic Alcohol Substrates^a^

a Reactions were carried out on a 1.0 mmol scale in MeCN ([**XX**]_0_ = 0.10 M). Yields refer to material isolated after purification by flash column chromatography. Yields in parentheses were determined by ^1^H NMR spectroscopy using 1,3,5-trimethoxybenzene as the internal standard.

To obtain insights into the differing reactivity of benzylic and aliphatic alcohols, density functional theory (DFT) studies were undertaken at the PBE0-D3 ^52,53^ /def2-TZVP^54,55^ /SMD (acetonitrile)^56^ level of theory. We initially considered the fragmentation of the phosphoranyl radical intermediate **14** in a manner analogous to that previously proposed by Doyle and co-workers.^16^ The energy profiles for the formation and subsequent reactions of phosphoranyl radicals **14a** and **14b** are shown in Figure 1.

**Figure 1:**
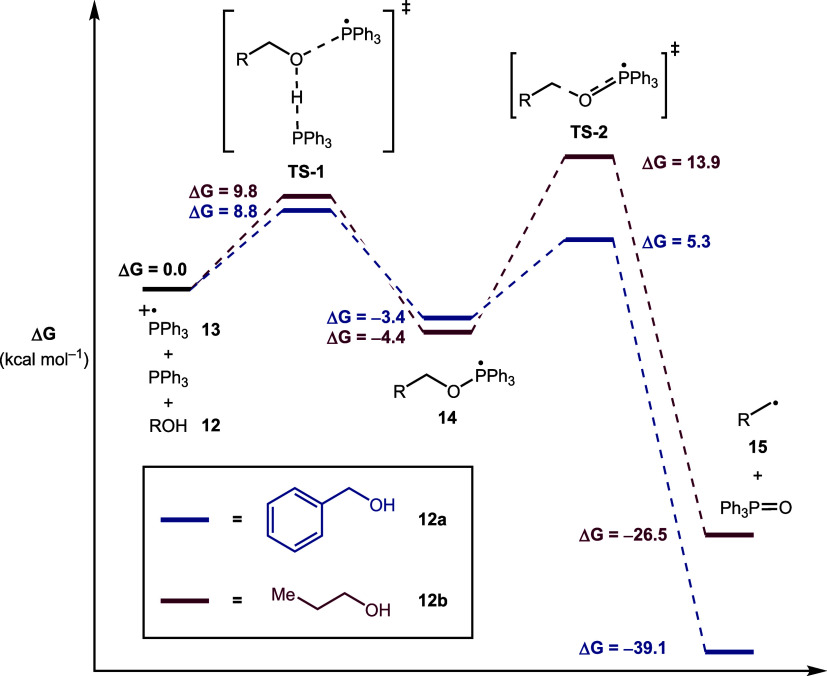
Computational modeling of phosphoranyl radical formation and fragmentation pathways in the electrochemical deoxygenation. Gibbs energies are relative to those of the starting materials and calculated at PBE0/def2-TZVP/SMD (MeCN).

Consistent with studies by Wang et al.,^57^ and the reported oxidation potentials for triphenylphosphine,^42^ oxidation of triphenylphosphine to the corresponding radical cation will readily occur at the anode. Reaction of alcohol **12** and triphenylphosphine radical cation **13** with concurrent deprotonation using triphenylphosphine as the base afforded phosphoranyl radical **14** via **TS-1**. Based on the results of the calculations, the difference in reactivity observed for benzylic and aliphatic alcohols could be attributed to the difference in energies between the fragmentation transition states **TS-2**, which, in the case of the aliphatic alcohol, is almost 10 kcal mol^–1^ higher than the barrier for the fragmentation of the benzylic alcohol derivative **14a**. Furthermore, in the case of the aliphatic alcohol **12b**, reversion to the starting materials has a lower energy barrier than that calculated for the fragmentation step, and as a result, this pathway is likely to predominate.

In contrast, in the case of benzyl alcohol **12a**, fragmentation of the phosphoranyl radical **14a** is lower in energy than the preceding step, making fragmentation favorable. While this is a plausible rationale for the reactivity observed by ourselves and that observed by Doyle and co-workers in their photoredox-mediated deoxygenation,^16^ we are cognizant of the fact that under the electrochemical conditions, oxidation of the phosphoranyl radical could also occur, opening up alternative mechanistic pathways.^41^

Based on the results of our experimental and computational studies and the existing literature, we propose two plausible mechanistic scenarios to explain the observed reactivity in this halide-free deoxygenation reaction (Scheme 3). In both cases, we believe that the reactions proceed via an initial anodic oxidation of triphenylphosphine to give phosphine radical cation **13**. Addition of alcohol **12a** to the phosphine radical cation **13** with concomitant deprotonation affords the phosphoranyl radical **14a** (Scheme 3A). In the case of the phosphoranyl radical derived from benzylic alcohol **12a**, this could fragment to afford benzylic radical **15a** along with triphenylphosphine oxide (Path A). The resulting radical would be readily oxidized (tolyl radical *E*_ox_ = 0.73 V vs SCE)^58^ to give the benzylic carbocation, which would be trapped with triphenylphosphine to afford the alkylphosphonium salt **17a**. Alkoxyphosphonium salt **17a** is then cathodically reduced to afford the deoxygenated product **18a**; such species have been found to undergo electrochemical reduction to give the desired product **18a** when subjected to our optimized reaction conditions (see the SI for details).

**Scheme 3:**
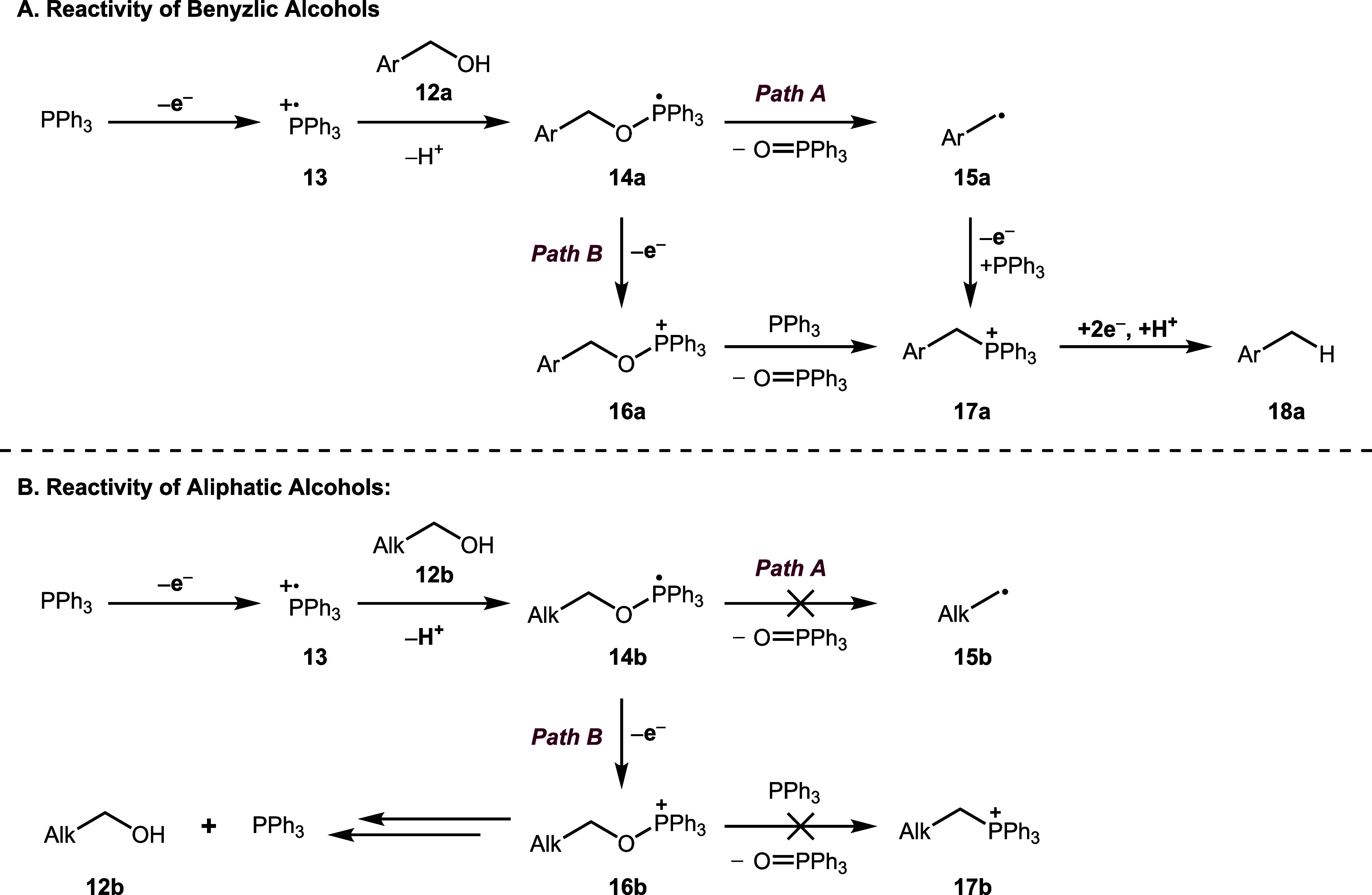
Plausible Mechanisms for Reactions Involving (A) Benzylic and (B) Aliphatic Alcohols

Alternatively, oxidation of the phosphoranyl radical could occur, giving rise to an alkoxyphosphonium salt **16a** (Path B).^51^ The formation of alkoxyphosphonium intermediates has also been proposed by Hu and co-workers in their recently developed “e-Mitsunobu” reaction,^59^ in which aliphatic alcohols are activated toward nucleophilic substitution using an electrogenerated phosphine radical cation. Alkoxyphosphonium salts derived from benzylic alcohols are known to react with triphenylphosphine via an Arbuzov reaction to give the alkylphosphonium salt **17a**,^32^ which we have shown undergoes reduction to afford product **18a**.

In the case of phosphoranyl radicals **14b** derived from aliphatic alcohols (Scheme 3B), the fragmentation is presumably disfavored due to the higher energy barrier associated with forming less stable alkyl radical **15b**, as corroborated by our computational studies. Furthermore, if oxidation of the phosphoranyl radical **14b** occurs (Path B), the corresponding alkoxyphosphonium salts **16b** derived from primary aliphatic alcohols are reported not to undergo the Arbuzov reaction with triphenylphosphine.^60^ If formed, reversion of alkoxyphosphonium salt **16b** to starting alcohol **12b** and triphenylphosphine would likely be the predominant pathway.

## Conclusions

We have developed a straightforward electrochemical method that enables the selective deoxygenation of benzylic alcohols. The reaction is metal- and halide-free and is completed in a single step, offering improved step economy over conventional two-step derivatization–cleavage sequences. The reaction proceeds under mild conditions and tolerates a range of functional groups, including esters, nitriles, boronate esters, and halides. Computational, mechanistic, and electroanalytical studies provide insight into the mechanism of C–O bond activation, offering a foundation for future efforts to exploit this strategy for the deoxygenative functionalization of alcohols.

## Supplementary Material



## Notes

Design of electrochemical cell, additional experimental and computational procedures, analytical data, including NMR spectra and chromatograms (PDF)
