# Supplementary material for: Metal-Free Direct Electrochemical Deoxygenation of Benzylic Alcohols
Source: ACS Sustain Chem Eng. 2025 Dec 2;13(49):21101–10. doi: 10.1021/acssuschemeng.5c08410 (PMC12709617; doi:10.1021/acssuschemeng.5c08410)
Supplement: Supplementary file 1 [file sc5c08410_si_001.pdf]

# Supporting Information

## Metal-free Direct Electrochemical Deoxygenation of Benzylic Alcohols

Alex G. Edmonds,[a,b] Darren A. Walsh,*[a,b] Kristaps Ermanis,*[b] James Cuthbertson*[a,b]

[a] *GlaxoSmithKline Carbon Neutral Laboratories for Sustainable Chemistry, University of Nottingham, Jubilee Campus, Triumph Road, Nottingham, NG7 2TU, U.K.*

[b] *School of Chemistry, University of Nottingham, University Park, Nottingham, NG7 2RD, U.K.*

*E-mail: darren.walsh@nottingham.ac.uk, kristaps.ermanis@nottingham.ac.uk, cuthbertson.james.d@gmail.com

80 Pages: Experimental procedures and characterisation data; Figures S1–S5; Tables S1–S12; NMR spectra; GC–MS chromatograms.

## Table of Contents

1. Design of Electrolysis Cell ..... S2
2. General Procedures for GC-MS Analysis ..... S4
3. Optimisation of the Reaction Conditions ..... S6
4. General Procedures ..... S12
5. Substrate Synthesis ..... S13
6. Deoxygenation of Benzylic Alcohols ..... S22
7. E-Factor ..... S33
8. Preparation of GC–MS Standards ..... S35
9. Mechanistic Studies ..... S37
10. Deoxygenation of an Allylic Alcohol ..... S41
11. Computational Studies ..... S42
12. References ..... S44
13. NMR Spectra of Substrates and Products ..... S49
14. GC–MS Chromatograms of Reaction Mixtures ..... S73

# 1. Design of Electrolysis Cell

The electrolysis cell was made in-house, and consists of a two-necked glass tube (internal diameter 21 mm, total length 150 mm) with B14 ground glass joint and side neck with B19 ground glass joint. A PTFE stopper with two PEEK screw fittings was made in-house according to designs published by Ackermann et al.[1] Electrode holders were made in-house, and consist of a 2 mm diameter stainless steel rod with affixed stainless steel plate, with a threaded hole for tightening a second stainless steel plate with a screw.

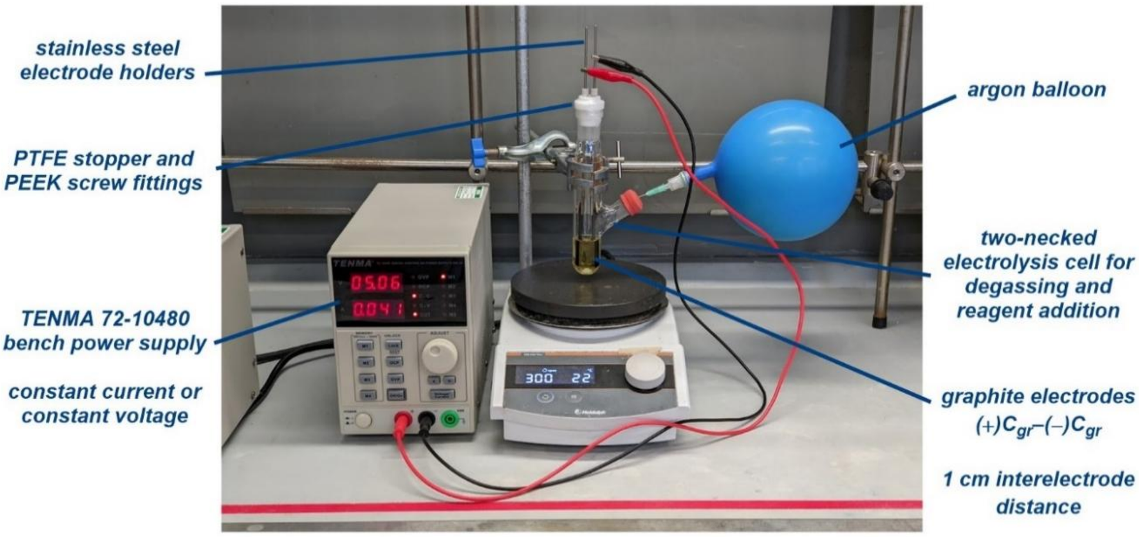


**Figure S1.** Electrolysis Cell in Operation

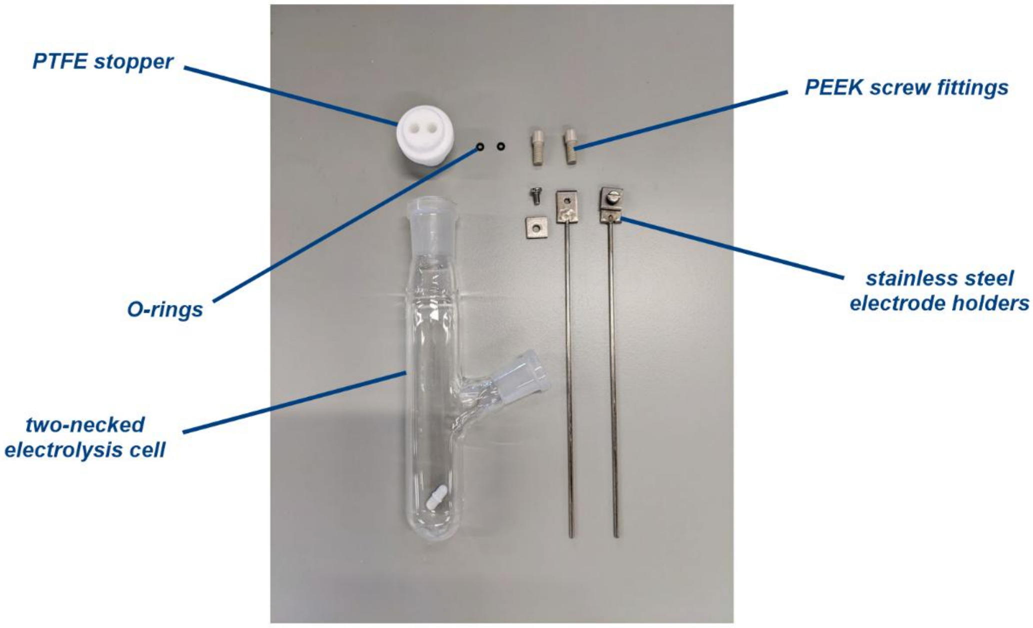


**Figure S2.** Disassembled electrolysis cell.

**Table S1.** Electrode materials and suppliers

| Electrode Material | Supplier | Supplier Product No. |
|---|---|---|
| Graphite ($C_{gr}$) | IKA | 0040002858 |
| Reticulated Vitreous Carbon (RVC) | Goodfellow | VC003830 |
| Nickel (Ni) | U.S. Solid | PNS00028 |
| Stainless Steel Mesh ($SS_{mesh}$) | Advent | FE621015 |
| Platinum wire, 0.368 mm dia. (Pt) | Thermo Scientific | 11322128 |

# 2. General Procedures for GC-MS Analysis

For yields determined by GC-MS analysis, the identity of the product was confirmed by comparison of the retention time with an authentic reference sample purchased from commercial sources or prepared using literature procedures. The authentic reference samples were also used for calibration purposes as detailed below.

**Preparation of Crude Reaction Mixture Sample:**

To the crude reaction mixture was added dodecane (227 μL, 1.00 mmol, 1.00 equiv.) or tetradecane (260 μL, 1.00 mmol, 1.00 equiv.) then the reaction mixture was diluted with $Et_2O$ (*ca.* 10 mL) and stirred. Approximately 1 mL of the diluted reaction mixture was added to a vial, and diluted with $Et_2O$ (*ca.* 19 mL), then approximately 1 mL of the resulting solution was added to a vial and diluted with $Et_2O$ (*ca.* 9 mL). The resulting solution was added to a GC–MS vial and submitted for analysis.

**Preparation of Calibration Samples:**

Solutions of the standard (0.4 mM in $Et_2O$) and the analyte (0.4 mM in $Et_2O$) were prepared. Solutions to acquire response factors for 20%, 40%, 60%, 80% and 100% analyte concentration vs standard concentration were prepared according to **Table S2**.

**Table S2.** Preparation of GC–MS Calibration Samples.

| **Analyte Concentration (vs. Standard)** | **20%** | **40%** | **60%** | **80%** | **100%** |
|---|---|---|---|---|---|
| Vol. 0.4 mM Analyte Solution (μL) | 100 | 200 | 300 | 400 | 500 |
| Vol. 0.4 mM Standard Solution (μL) | 500 | 500 | 500 | 500 | 500 |
| Vol. $Et_2O$ (μL) | 400 | 300 | 200 | 100 | 0 |
| Total Volume (μL) | 1000 | 1000 | 1000 | 1000 | 1000 |
| [Analyte] (mM) | 0.04 | 0.08 | 0.12 | 0.16 | 0.20 |
| [Standard] (mM) | 0.20 | 0.20 | 0.20 | 0.20 | 0.20 |

**GC–MS Instrument Method:**

Gas chromatography–mass spectrometry (GC–MS) was performed on a Thermo Scientific Trace 1300 Gas Chromatograph equipped with Thermo Trace Gold TG17-MS column (length: 30 m; diameter: 0.25 mm; film thickness: 0.25 μm) and Thermo Scientific ISQ LT Single Quadrupole Mass Spectrometer.

Samples were injected using the splitless injection technique and carried using helium at a flow rate of 1.50 mL/min. The oven temperature was held at 40 °C for 3 minutes, then a heating ramp of 10 °C/min was applied until 260 °C was reached, after which the temperature was held at 260 °C for 10 minutes (**Figure S3**).

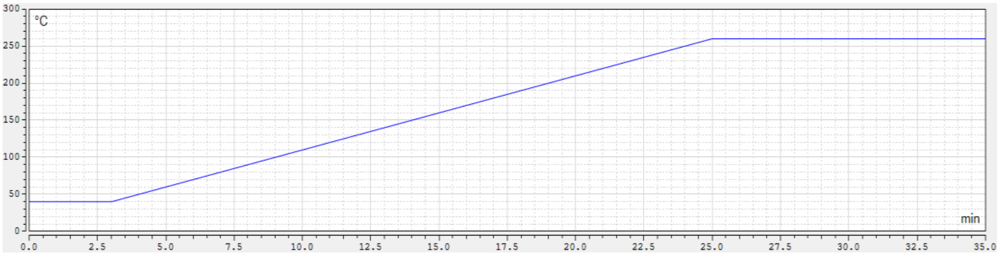


**Figure S3.** GC–MS Oven temperature profile.

Relative component concentrations were determined from total ion counts acquired by the mass spectrometer.

**Determination of Reaction Yield from GC–MS Chromatogram:**

Five solutions of the analyte at concentrations of 0.04, 0.08, 0.12, 0.16 and 0.20 mM were prepared, each containing a standard at a concentration of 0.20 mM. The Response Factor (ratio of total analyte ion counts to total standard ion counts) was determined from the GC–MS chromatogram.

$$Response\ Factor = \frac{Peak\ Area_{Analyte}}{Peak\ Area_{Standard}}$$

For each analyte, Response Factor was plotted against Analyte Concentration to produce a linear line of best fit with gradient $m$.

The response factor of the analyte in the crude reaction mixture sample was determined, and used to calculate the concentration of the analyte in the crude reaction mixture.

$$Concentration_{Analyte} = \frac{Response\ Factor_{Analyte}}{m_{Analyte}}$$

# 3. Optimisation of the Reaction Conditions

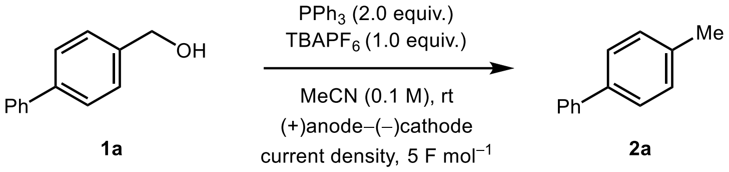


**Scheme S1.** Standard procedure for the optimisation reaction

The electrolysis was carried out in an undivided cell with two electrodes. An oven-dried two-necked cell equipped with a magnetic stir bar was charged with alcohol **1a** (1.00 mmol, 184 mg), phosphine, electrolyte and anhydrous solvent (10 mL). After sparging the solution with argon for 5 minutes, a constant current was applied for the required amount of time. After reaction completion, 1,3-benzodioxole (105 µL, 1.02 mmol, 1.02 equiv.) was added as an internal standard, and the yield of the reaction was determined by quantitative $^1$H NMR spectroscopy.

**Table S3.** Optimisation of the current density for the deoxygenation reaction.

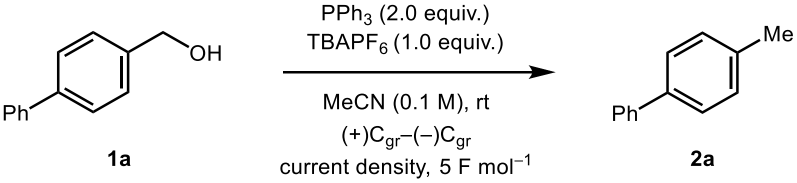


| Entry | Current Density (mA/cm$^2$) | Conversion[a] (%) | Yield[a] (%) | Reaction Time (min) |
|---|---|---|---|---|
| 1 | 5 | 100 | 97 | 804 |
| 2 | 20 | 100 | 98 | 201 |
| 3 | 30 | 100 | 94 | 134 |
| 4 | 40 | 100 | 75 | 101 |

Optimisation reactions conducted on a 1.00 mmol scale. [a]Conversions and yields were determined by $^1$H NMR spectroscopy using 1,3-benzodioxole as an internal standard.

**Table S4.** Optimisation of the equivalents of charge for the deoxygenation reaction.

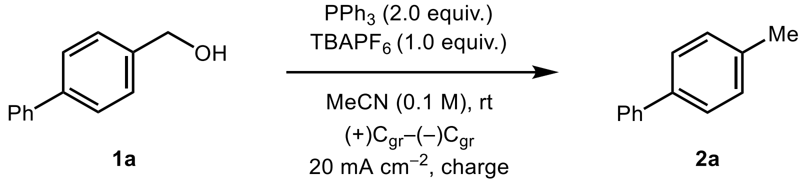


| Entry | Equiv. Charge (F/mol) | Conversion[a] (%) | Yield[a] (%) | Reaction Time (min) |
|---|---|---|---|---|
| 1 | 2.5 | 93 | 86 | 101 |
| 2 | 4.0 | 100 | 92 | 161 |
| 3 | 5.0 | 100 | 98 | 201 |

Optimisation reactions conducted on a 1.00 mmol scale. [a]Conversions and yields were determined by $^{1}H$ NMR spectroscopy using 1,3-benzodioxole as an internal standard.

**Table S5.** Optimisation of the anode and cathode materials for the deoxygenation reaction.

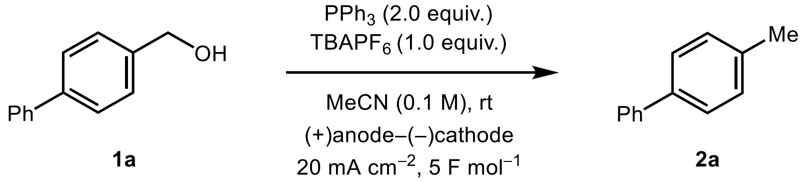


| Entry | Anode | Cathode | Conversion[a] (%) | Yield[a] (%) |
|---|---|---|---|---|
| 1 | $C_{gr}$ | $C_{gr}$ | 100 | 98 |
| 2 | $C_{gr}$ | Pt wire | 100 | 86 |
| 3 | $C_{gr}$ | Ni | 100 | 91 |
| 4 | Pt wire | $C_{gr}$ | 98 | 55 |
| 5 | Pt wire | Pt wire | 91 | 36 |
| 6 | RVC | RVC | 97 | 93 |
| 7 | $SS_{mesh}$ | $SS_{mesh}$ | 35 | 27 |

Optimisation reactions conducted on a 1.00 mmol scale. [a]Conversions and yields were determined by $^{1}H$ NMR spectroscopy using 1,3-benzodioxole as an internal standard.

**Table S6.** Optimisation of the phosphine for the deoxygenation reaction.

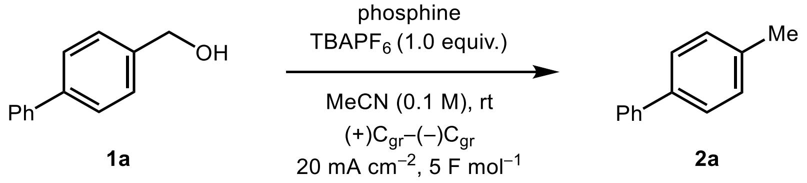


| Entry | Phosphine | Equiv. | Conversion[a] (%) | Yield[a] (%) |
|---|---|---|---|---|
| 1 | $PPh_3$ | 2.00 | 100 | 98 |
| 2 | $PPh_3$ | 1.50 | 100 | 76 |
| 3[b] | $PPh_3$ | 1.00 | 53 | 54 |
| 4 | $P(OEt)_3$ | 2.00 | 100 | 0 |
| 5 | $P(OPh)_3$ | 2.00 | 100 | 0 |

Optimisation reactions conducted on a 1.00 mmol scale. [a]Conversions and yields were determined by $^1$H NMR spectroscopy using 1,3-benzodioxole as an internal standard. [b]Reaction halted before complete delivery of 5 F/mol due to 10 V power supply safety limit.

**Table S7.** Optimisation of the supporting electrolyte for the deoxygenation reaction.

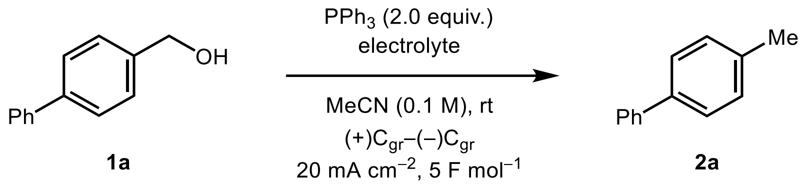


| Entry | Supporting Electrolyte | Equiv. | Conversion[a] (%) | Yield[a] (%) |
|---|---|---|---|---|
| 1 | $TBAPF_6$ | 1.0 | 100 | 98 |
| 2 | $TBAPF_6$ | 0.5 | 100 | 91 |
| 3 | $LiPF_6$ | 1.0 | 32 | 6 |
| 4 | $TBAClO_4$ | 1.0 | 100 | 77 |
| 5 | $TBABF_4$ | 1.0 | 100 | 91 |
| 6 | $TEABF_4$ | 1.0 | 100 | 83 |

Optimisation reactions conducted on a 1.0 mmol scale. [a]Conversions and yields were determined by $^1H$ NMR spectroscopy using 1,3-benzodioxole as an internal standard.

**Table S8.** Optimisation of the solvent for the deoxygenation reaction.

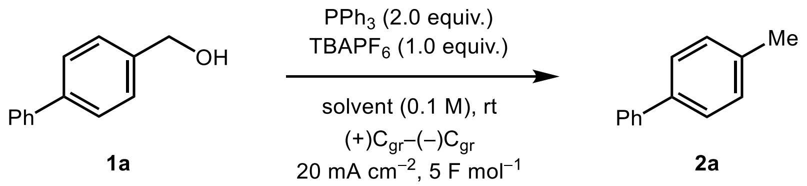


| Entry | Solvent | Conversion[a] (%) | Yield[a] (%) |
|---|---|---|---|
| 1 | MeCN | 100 | 98 |
| 2 | THF | 100 | 75 |
| 3 | $CH_2Cl_2$ | 84 | 53 |

Optimisation reactions conducted on a 1.0 mmol scale. [a]Conversions and yields were determined by $^1H$ NMR spectroscopy using 1,3-benzodioxole as an internal standard.

**Table S9.** Control reactions for the deoxygenation reaction.

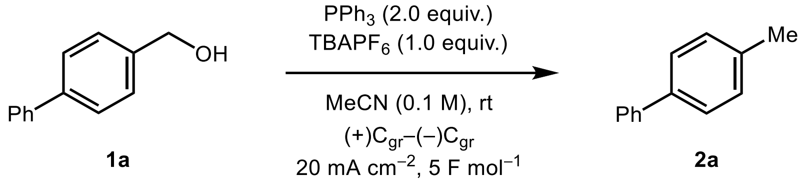


| Entry | Deviation from Above Conditions | Conversion[a] (%) | Yield[a] (%) |
|---|---|---|---|
| 1 | none | 100 | 98 |
| 2 | no electricity | 7 | 0 |
| 3 | no phosphine | 91 | 21 |
| 4 | air atmosphere | 100 | 68 |
| 5 | unstirred | 99 | 57 |
| 6 | 0.50 mmol scale | 100 | 82 |

Optimisation reactions conducted on a 1.0 mmol scale. [a]Conversions and yields were determined by $^1$H NMR spectroscopy using 1,3-benzodioxole as an internal standard.

**Table S10.** Additive screen for the deoxygenation reaction.

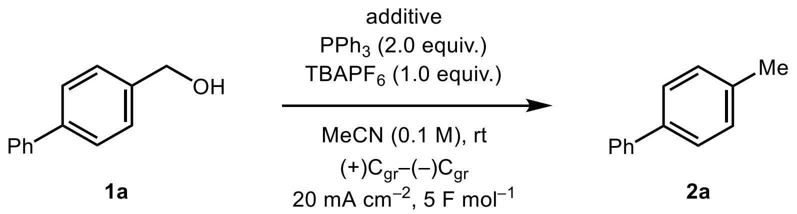


| Entry | Additive | Conversion[a] (%) | Yield[a] (%) |
|---|---|---|---|
| 1 | none | 100 | 98 |
| 2 | water (10 eq.) | 73 | 47 |
| 3 | 1-hexene (2.0 eq.) | 100 | 69 |
| 4 | PTSA (1.0 eq.) | 97 | 52 |
| 5 | 2,6-lutidine (1.0 eq.) | 98 | 79 |
| 6 | acetone (10 eq.) | 100 | 84 |

Optimisation reactions conducted on a 1.0 mmol scale. [a]Conversions and yields were determined by $^{1}H$ NMR spectroscopy using 1,3-benzodioxole as an internal standard.

# 4. General Procedures

### General Procedure A – Grignard Addition to Aldehydes:

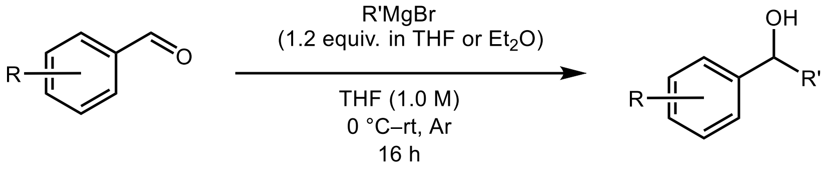


To a stirred solution of the aldehyde (10.0 mmol) in anhydrous THF (10 mL) under argon at 0 °C was added dropwise over 2 min the Grignard reagent (solution in THF or $Et_2O$, 12.0 mmol, 1.20 equiv.). The solution was allowed to warm to room temperature and stirred for 16 hours, then the reaction was quenched with sat. aq. $NH_4Cl$ (5 mL). The reaction mixture was diluted with $H_2O$ (5 mL) and EtOAc (25 mL), then the organic phase was separated. The aqueous phase was extracted with EtOAc (2 × 50 mL), then the combined organic extracts were dried ($MgSO_4$), filtered, concentrated *in vacuo*. Purification by silica gel column chromatography afforded the desired product.

### General Procedure B - Electrochemical Deoxygenation of Benzylic Alcohols:

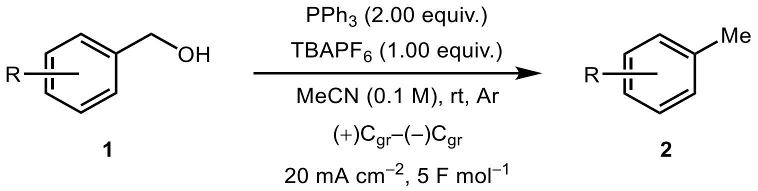


The electrolysis was carried out in an undivided cell with a graphite anode (2 mm × 10 mm × 25 mm submerged volume) and a graphite cathode (2 mm × 10 mm × 25 mm submerged volume). An oven-dried two-necked cell equipped with a magnetic stir bar was charged with the benzylic alcohol (1.00 mmol), triphenylphosphine (525 mg, 2.00 mmol, 2.00 equiv.), tetrabutylammonium hexafluorophosphate (387 mg, 1.00 mmol, 1.00 equiv.) and anhydrous acetonitrile (10 mL). After sparging the solution with argon for 5 minutes, a constant current of 41 mA (20 mA/$cm^2$) was applied for 3.27 h (5 F/mol) under an argon atmosphere.

For volatile products, yields were determined by $^1$H NMR spectroscopic analysis of the crude reaction mixture using 1,3,5-trimethoxybenzene as an internal standard, and GC–MS analysis of the crude reaction mixture using dodecane or tetradecane as an external standard.

For non-volatile products, the electrodes were removed from solution and rinsed with MeCN (1 mL). The electrode washings were combined with the reaction mixture, then the solvent was removed *in vacuo*. Purification by column chromatography afforded the desired product.

# 5. Substrate Synthesis

***tert*-Butyl(4-(hydroxymethyl)phenyl)carbamate (1f)**[2]

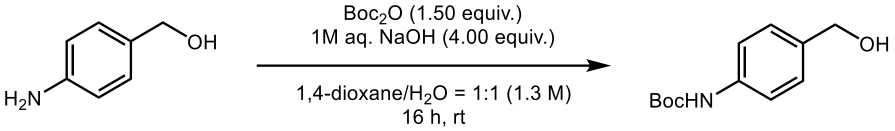


**1f**

To a stirred solution of 4-aminobenzyl alcohol (493 mg, 4.00 mmol, 1.00 equiv.) and di-*tert*-butyl dicarbonate (1.31 g, 6.00 mmol, 1.50 equiv.) in 1,4-dioxane/water (1:1) (3 mL) was added 1 M aq. NaOH (4.00 mL, 4.00 mmol, 1.00 equiv.). The reaction was mixture stirred at room temperature for 16 hours, then was quenched with $H_2O$ (10 mL). The crude product was extracted into EtOAc (2 × 10 mL). The combined organic extracts were washed with 1 M aq. HCl (2 × 10 mL), brine (10 mL), dried ($MgSO_4$) and concentrated *in vacuo* to afford a colourless oil. Purification of the crude product by flash column chromatography ($SiO_2$, Cyclohexane : EtOAc, 7:3) gave the *title compound* **1f** (885 mg, 4.00 mmol, quant.) as a colourless oil; $R_f$ = 0.12 (Cyclohexane / EtOAc, 7:3); $^1$H NMR (400 MHz, $CDCl_3$) $\delta_H$ 7.29 (d, $J$ = 8.3 Hz, 2H), 7.21 (d, J = 8.3 Hz, 2H), 6.78 (s, 1H), 4.55 (s, 2H), 2.51, (s, 1H), 1.50 (s, 9H); $^{13}$C NMR (101 MHz, $CDCl_3$) $\delta_C$ 153.0 (C), 137.8 (C), 135.7 (C), 127.9 (CH), 118.8 (CH), 80.6 (C), 64.8 ($CH_2$), 28.4 ($CH_3$). The analytical data are consistent with those reported in the literature.[3]

**(4-(Tetrahydrofuran-3-yl)phenyl)methanol (1o)**

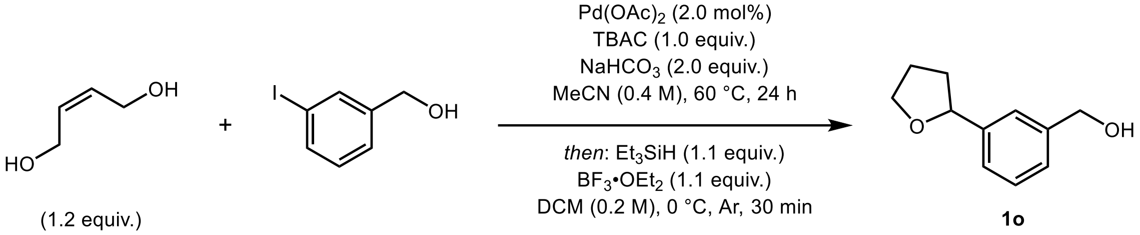


**1o**

Prepared according to conditions described in the literature.[4] To a stirred solution of palladium(II) acetate (22.5 mg, 100 µmol, 0.02 equiv.), tetrabutylammonium chloride (1.39 g, 5.00 mmol, 1.00 equiv.) and sodium hydrogencarbonate (840 mg, 10.0 mmol, 2.00 equiv.) in anhydrous acetonitrile (12.5 mL) under argon was added (*Z*)-but-2-ene-1,4-diol (493 µL, 6.00 mmol, 1.20 equiv.) and (3-iodophenyl)methanol (635 µL, 5.00 mmol, 1.00 equiv.). After stirring at 60 °C for 24 h, the mixture was allowed to cool to room temperature, then diluted with $Et_2O$ (50 mL). The mixture was washed with $H_2O$ (100 mL) and brine (50 mL), dried ($MgSO_4$), filtered and concentrated *in vacuo* to afford a brown oil. The crude product was dissolved in DCM (25 mL) under argon at 0 °C. Triethylsilane (876 µL, 5.50 mmol, 1.10 equiv.) was added, then boron trifluoride etherate (679 µL, 5.50 mmol, 1.10 equiv.) was added dropwise over 1 minute. After stirring at 0 °C for 30 minutes, the reaction mixture was

quenched with sat. aq. $NaHCO_3$ (50 mL). The organic phase was separated then the aqueous phase was extracted with DCM (3 × 25 mL). The combined organic phases were washed with brine (2 × 25 mL), dried ($MgSO_4$), filtered and concentrated *in vacuo* to afford the crude product as a yellow oil. Purification of the crude product by flash column chromatography ($SiO_2$, Cyclohexane : EtOAc, 9:1 → 4:1) gave the *title compound* **1o** (221 mg, 1.24 mmol, 25%) as a colourless oil; $R_f = 0.16$ (Cyclohexane / EtOAc, 1:1); $\nu_{max}/cm^{-1}$ (ATR) 3373, 2931, 2867, 1044; $^1H$ NMR (400 MHz, $CDCl_3$) $\delta_H$ 7.31, (app. t, $J = 7.6$ Hz, 1H), 7.26 (s, 1H), 7.22 (d, $J = 7.6$ Hz, 1H), 7.18 (d, $J = 7.6$ Hz, 1H), 4.68 (s, 2H), 4.13 (app. t, $J = 8.0$ Hz, 1H), 4.06 (app. td, $J = 8.3, 4.5$ Hz, 1H), 3.91 (app. q, $J = 8.3$ Hz, 1H), 3.72 (app. t, $J = 8.2$ Hz, 1H), 3.41 (app. p, $J = 7.8$ Hz, 1H), 2.37 (app. dtd, $J = 15.6, 7.4, 4.5$ Hz, 1H), 2.01 (app. dq, J = 12.4, 8.1 Hz, 1H), 1.97–1.94 (m, 1H); $^{13}C$ NMR (101 MHz, $CDCl_3$) $\delta_C$ 143.2 (C), 141.4 (C), 128.9 (CH), 126.6 (CH), 126.0 (CH), 125.3 (CH), 74.7 ($CH_2$), 68.6 ($CH_2$), 65.4 ($CH_2$), 45.1 (CH), 34.7 ($CH_2$); HRMS (ESI) [$M+Na^+$] calculated for $C_{11}H_{14}NaO_2$: 201.0886, found: 201.0879 (3.60 ppm error).

### *N*-(3-(Hydroxymethyl)phenyl)cyclohexanecarboxamide (1q)

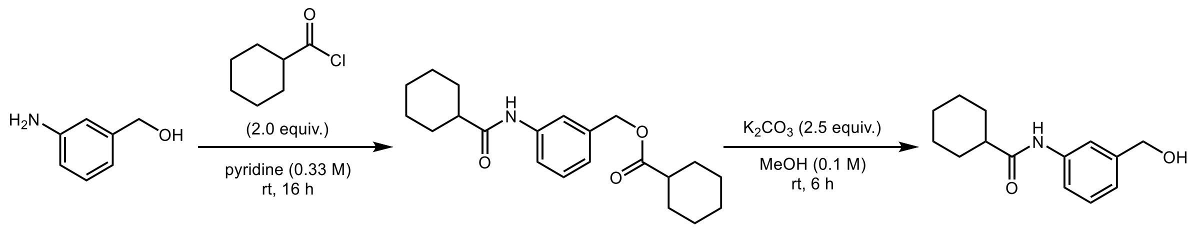


S1 1q

**Step 1: Synthesis of 3-(Cyclohexanecarboxamido)benzyl cyclohexanecarboxylate (S1):** To a stirred solution of 3-aminobenzyl alcohol (615 mg, 5.00 mmol, 1.00 equiv.) in pyridine (15 mL) was added dropwise over 2 min cyclohexanecarbonyl chloride (1.61 mL, 10.0 mmol, 2.00 equiv.). The reaction mixture was stirred at room temperature for 16 hours, then quenched with $H_2O$ (50 mL). The crude product was extracted into EtOAc (3 × 50 mL). The combined organic extracts were dried ($MgSO_4$), filtered, and concentrated *in vacuo* to afford a colourless oil. Purification of the crude product by flash column chromatography ($SiO_2$, Cyclohexane : EtOAc, 9:1) gave the *title compound* **S1** (1.64 g, 4.78 mmol, 96%) as a colourless solid; m.p. 80–82 °C; $R_f = 0.38$ (Cyclohexane / EtOAc, 7:3); $\nu_{max}/cm^{-1}$ (ATR) 3289, 2926, 2852, 1736, 1651; $^1H$ NMR (400 MHz, $CDCl_3$) $\delta_H$ 7.52–7.49 (m, 2H), 7.29 (dd, $J = 7.8, 7.7$ Hz, 1H), 7.19 (s, 1H), 7.07 (d, $J = 7.7$ Hz, 1H), 5.06 (s, 2H), 2.35 (tt, $J = 11.4, 3.7$ Hz, 1H), 2.22 (tt, $J = 11.8, 3.5$ Hz, 1H), 1.97–1.20 (m, 20H); $^{13}C$ NMR (101 MHz, $CDCl_3$) $\delta_C$ 176.1 (C), 174.6 (C), 138.4 (C), 137.3 (C), 129.3 (CH), 123.7 (CH), 119.6 (CH), 119.4 (CH), 65.9 ($CH_2$), 46.7 (CH), 43.3 (CH), 29.8 ($CH_2$), 29.1 ($CH_2$), 25.9 ($CH_2$), 25.8 ($CH_2$), 25.6 ($CH_2$), 25.5 ($CH_2$); HRMS (ESI) $[M+H]^+$ calculated for $C_{21}H_{30}NO_3$: 344.2220, found: 344.2215 (1.50 ppm error).

**Step 2: Synthesis of *N*-(3-(Hydroxymethyl)phenyl)cyclohexanecarboxamide (1q):** To a stirred solution of 3-(cyclohexanecarboxamido)benzyl cyclohexanecarboxylate (**S1**, 1.37 g, 4.00 mmol, 1.00 equiv.) in methanol (40 mL) was added portionwise potassium carbonate (1.38 g, 10.0 mmol, 2.50

equiv.). The reaction mixture was stirred at room temperature for 6 hours, then diluted with $H_2O$ (50 mL). The crude product was extracted into EtOAc (3 × 25 mL), then the combined organic extracts were dried ($MgSO_4$), filtered, and concentrated *in vacuo* to afford a colourless solid. Purification of the crude product by flash column chromatography ($SiO_2$, Cyclohexane : EtOAc, 1:1) gave the *title compound* **1q** (818 mg, 3.52 mmol, 88%) as a colourless solid; m.p. 117–120 °C; $R_f$ = 0.18 (Cyclohexane / EtOAc, 1:1); $\nu_{max}/cm^{-1}$ (ATR) 3305, 3256, 2921, 2851, 1656, 1616; $^1H$ NMR (400 MHz, $CDCl_3$) $\delta_H$ 7.56 (s, 1H), 7.41 (d, $J$ = 7.8 Hz, 1H), 7.34 (s, 1H), 7.29 (t, $J$ = 7.8 Hz, 1H), 7.07 (d, $J$ = 7.8 Hz, 1H), 4.63 (s, 2H), 2.26–2.18 (1H, m), 2.15 (s, 1H), 1.95–1.92 (m, 2H), 1.84–1.81 (m, 2H), 1.72–1.68 (m, 2H), 1.58–1.48 (m, 2H), 1.36–1.19 (m, 3H); $^{13}C$ NMR (101 MHz, $CDCl_3$) $\delta_C$ 174.7 (C), 142.1 (C), 138.4 (C), 129.3 (CH), 122.7 (CH), 119.1 (CH), 118.5 (CH), 65.1 ($CH_2$), 46.6 (CH), 29.8 ($CH_2$), 25.8 ($CH_2$), 25.8 ($CH_2$); HRMS (ESI) $[M+Na]^+$ calculated for $C_{14}H_{19}NNaO_2$: 256.1308, found: 256.1300 (3.00 ppm error).

**4-(*tert*-Butyl)-*N*-(3-(hydroxymethyl)phenyl)benzenesulfonamide (1r)**

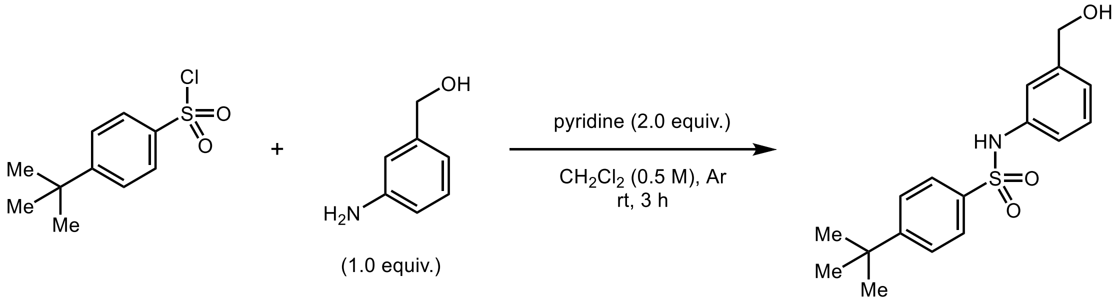


**1r**

A stirred solution of 4-(*tert*-butyl)benzenesulfonyl chloride (1.16 g, 5.00 mmol, 1.00 equiv.) and (3-aminophenyl)methanol (616 mg, 5.00 mmol, 1.00 equiv.) in DCM (10 mL) under argon was sparged with argon for 5 minutes. Pyridine (809 μL, 10.0 mmol, 2.00 equiv.) was added dropwise over 1 minute. The reaction mixture was stirred at room temperature for 3 hours, then poured into 1 M aq. HCl (20 mL). The organic phase was separated, and the aqueous phase was extracted with EtOAc (2 × 25 mL). The combined organic phases were washed with $H_2O$ (25 mL) and brine (25 mL), dried ($MgSO_4$), filtered and concentrated *in vacuo* to afford the crude product as a brown oil. Purification of the crude product by flash column chromatography ($SiO_2$, Cyclohexane : $Et_2O$, 1:1) gave the *title compound* **1r** (1.49 g, 4.65 mmol, 93%) as a colourless crystalline solid; m.p. 127–129°C; $R_f$ = 0.36 (Cyclohexane : EtOAc, 1:1); $\nu_{max}/cm^{-1}$ (ATR) 3482, 3143, 2966, 2917, 2873, 1153; $^1H$ NMR (400 MHz, $CDCl_3$) $\delta_H$ 7.70 (d, $J$ = 8.7 Hz, 2H), 7.44 (d, $J$ = 8.7 Hz, 2H), 7.24 (app. t, $J$ = 7.8 Hz, 1H), 7.11 (d, $J$ = 7.8 Hz, 1H), 7.07 (s, 1H), 7.01 (d, $J$ = 7.8 Hz, 1H), 6.64 (s, 1H), 4.62 (d, $J$ = 5.5 Hz, 2H), 1.70 (t, $J$ = 5.5 Hz, 1H), 1.30 (s, 9H); $^{13}C$ NMR (101 MHz, $CDCl_3$) $\delta_C$ 157.0 (C), 142.4 (C), 137.1 (C), 136.2 (C), 129.6 (CH), 127.2 (CH), 126.2 (CH), 123.6 (CH), 120.2 (CH), 119.7 (CH), 64.8 ($CH_2$), 35.3 (C), 31.2 ($CH_3$); HRMS (ESI) $[M+Na]^+$ calculated for $C_{17}H_{21}NO_3S$: 342.1134, found: 342.1134 (0.00 ppm error).

**(4'-Vinyl-[1,1'-biphenyl]-4-yl)methanol (1t)**[5]

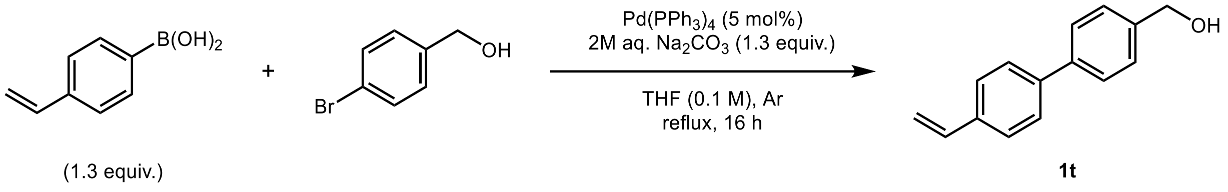


**1t**

A stirred solution of 4-bromobenzyl alcohol (935 mg, 5.00 mmol, 1.00 equiv.), 4-vinylbenzeneboronic acid (962 mg, 6.50 mmol, 1.30 equiv.) and tetrakis(triphenylphosphine)palladium(0) (289 mg, 250 µmol, 5 mol%) in anhydrous THF (50 mL) under argon was sparged with argon for 10 minutes. A solution of sodium carbonate (689 mg, 6.50 mmol, 1.30 equiv.) in water (3.25 mL) was sparged with argon for 10 minutes, then added to the reaction mixture. The mixture was heated at reflux for 16 hours, then allowed to cool to room temperature before $H_2O$ (50 mL) and EtOAc (50 mL) were added. The organic phase was separated, and the aqueous phase extracted with EtOAc (2 × 50 mL). The combined organic extracts were dried ($MgSO_4$), filtered, and concentrated *in vacuo*. Purification of the crude product by flash column chromatography ($SiO_2$, Cyclohexane : EtOAc, 8:2) and then recrystallisation from boiling toluene gave the *title compound* **1t** (233 mg, 1.11 mmol, 22%) as off-white flakes; m.p. 157–159 °C (lit. 156–157 °C)[5]; $R_f$ = 0.11 (Cyclohexane / EtOAc, 8:2); $^1$H NMR (400 MHz, $CDCl_3$) $\delta_H$ 7.61 (d, $J$ = 8.1 Hz, 2H), 7.57 (d, $J$ = 8.1 Hz, 2H), 7.49 (d, $J$ = 8.1 Hz, 2H), 7.44 (d, $J$ = 8.1 Hz, 2H), 6.77 (dd, $J$ = 17.5, 10.9 Hz, 1H), 5.81 (d, $J$ = 17.5 Hz, 1H), 5.29 (d, $J$ = 10.9 Hz, 1H), 4.74 (s, 2H), 1.80 (s, 1H); $^{13}$C NMR (101 MHz, $CDCl_3$) $\delta_C$ 140.3 (CH), 140.1 (CH), 136.8 (CH), 136.5 (CH), 131.8 (CH), 128.7 (CH), 127.6 (CH), 127.3 (CH), 126.8 (CH), 114.4 (CH), 65.3 ($CH_2$). The analytical data are consistent with those reported in the literature.[5]

**Methyl 4-(1-hydroxyethyl)benzoate (3c)**

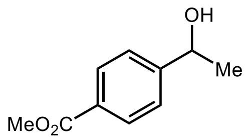


**3c**

Prepared according to General Procedure A, using methyl 4-formylbenzoate (1.64 g, 10.0 mmol, 1.00 equiv.) and methylmagnesium bromide solution (3.0 M in THF, 3.33 mL, 10.0 mmol, 1.00 equiv.). Purification of the crude product by flash column chromatography ($SiO_2$, Cyclohexane : EtOAc, 8:2) gave the *title compound* **3c** (994 mg, 5.51 mmol, 55%) as a pale yellow oil; $R_f$ = 0.11 (Cyclohexane / EtOAc, 8:2); $^1$H NMR (400 MHz, $CDCl_3$) $\delta_H$ 7.94 (d, $J$ = 8.4 Hz, 2H), 7.38 (d, $J$ = 8.4 Hz, 2H), 4.89 (q, $J$ = 6.5 Hz, 1H), 3.86 (s, 3H), 2.76 (s, 1H), 1.45 (d, $J$ = 6.5 Hz, 3H); $^{13}$C NMR (101 MHz, $CDCl_3$) $\delta_C$ 167.1 (C), 151.9 (C), 129.8 (CH), 129.1 (C), 125.4 (C), 69.9 (CH), 52.2 ($CH_3$), 25.3 ($CH_3$). The analytical data are consistent with those reported in the literature.[6]

**1-([1,1'-Biphenyl]-4-yl)propan-1-ol (3d)**

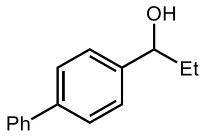

**3d**

Prepared according to General Procedure A, using biphenyl-4-carboxaldehyde (1.82 g, 10.0 mmol, 1.00 equiv.) and ethylmagnesium bromide solution (1.0 M in THF, 12 mL, 12.0 mmol, 1.20 equiv.). Purification of the crude product by flash column chromatography ($SiO_2$, Cyclohexane : EtOAc, 8:2) gave the *title compound* **3d** (1.84 g, 8.69 mmol, 87%) as a colourless solid; m.p. 59–62 °C (lit. 58–59 °C)[7]; $R_f$ = 0.24 (Cyclohexane / EtOAc, 8:2); $^1$H NMR (400 MHz, $CDCl_3$) $\delta_H$ 7.61–7.58 (m, 4H), 7.46–7.41 (m, 4H), 7.35 (tt, *J* = 7.3, 2.1 Hz, 1H), 4.66 (td, *J* = 6.6, 3.4 Hz, 1H), 1.93–1.75 (m, 3H), 0.96 (t, *J* = 7.4 Hz, 3H); $^{13}$C NMR (101 MHz, $CDCl_3$) $\delta_C$ 143.8 (C), 141.0 (C), 140.6 (C), 128.9 (CH), 127.4 (CH), 127.3 (CH), 127.2 (CH), 126.6 (CH), 75.9 (CH), 32.0 ($CH_2$), 10.3 ($CH_3$). The analytical data are consistent with those reported in the literature.[8]

**[1,1'-Biphenyl]-4-yl(cyclopropyl)methanol (3e)**

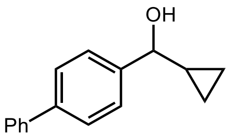

**3e**

Magnesium turnings (1.46 g, 60.0 mmol, 6.00 equiv.) and an iodine crystal were heated under an argon atmosphere until a purple vapor formed. After cooling to room temperature, anhydrous THF (12.0 mL) was added. The reaction mixture was heated to reflux, then bromocyclopropane (1.45 g, 12.0 mmol, 1.20 equiv.) was added dropwise over 2 minutes. After stirring for 2 hours at reflux, the solution of Grignard reagent was added to a stirred solution of biphenyl-4-carboxaldehyde (1.82 g, 10.0 mmol, 1.00 equiv.) in anhydrous THF (100 mL) under argon. The mixture was stirred at room temperature for 48 hours, then quenched with 1 M aq. HCl (60 mL). After stirring at room temperature for 1 hour, the mixture was diluted with EtOAc (50 mL) and $H_2O$ (25 mL). The aqueous phase was separated, then the organic phase was washed with $H_2O$ (50 mL) and brine (50 mL). The organic phase was dried ($MgSO_4$) and concentrated *in vacuo*. Purification of the crude product by flash column chromatography ($SiO_2$, Cyclohexane / EtOAc, 9:1→8:2) gave the *title compound* **3e** (612 mg, 2.73 mmol, 27%) as an orange crystalline solid; m.p. 69–71 °C; $R_f$ = 0.26 (Cyclohexane : EtOAc, 8:2); $^1$H NMR (400 MHz, $CDCl_3$) $\delta_H$ 7.61–7.59 (m, 4H), 7.51 (d, *J* = 8.3 Hz, 2H), 7.45 (app. t, *J* = 7.7 Hz, 2H) 7.35 (t, *J* = 7.3 Hz, 1H), 4.07 (d, *J* = 8.4 Hz, 1H), 1.94 (s, 1H), 1.32–1.23 (m, 1H), 0.71–0.57 (m, 2H), 0.55–0.49 (m, 1H), 0.46–0.40 (m, 1H); $^{13}$C NMR (101 MHz, $CDCl_3$) $\delta_C$ 143.0 (C), 141.1 (C), 140.7 (C), 128.9 (CH), 127.4 (CH),

127.3 (CH), 127.3 (CH), 126.6 (CH), 78.5 (CH), 19.3 (CH), 3.7 ($CH_2$), 3.1 ($CH_2$). The analytical data are consistent with those reported in the literature.[9]

**3-(4-(Hydroxymethyl)phenyl)propan-1-ol (7a)**

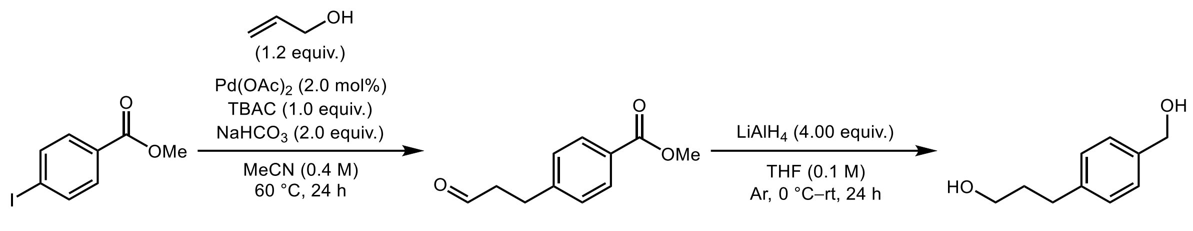


**Step 1: Synthesis of Methyl 4-(3-oxopropyl)benzoate (S2):** Prepared according to conditions described in the literature.[4] To a stirred solution of palladium(II) acetate (45.0 mg, 200 µmol, 0.02 equiv.), tetrabutylammonium chloride (2.78 g, 10.0 mmol, 1.00 equiv.) and sodium hydrogencarbonate (1.68 g, 20.0 mmol, 2.00 equiv.) in anhydrous acetonitrile (25 mL) under argon was added allyl alcohol (816 µL, 12.0 mmol, 1.20 equiv.) and methyl 4-iodobenzoate (2.62 g, 10.0 mmol, 1.00 equiv.). After stirring at 60 °C for 24 h, the mixture was allowed to cool to room temperature, then diluted with $Et_2O$ (50 mL). The mixture was washed with $H_2O$ (100 mL) and brine (50 mL), dried ($MgSO_4$), filtered and concentrated *in vacuo* to afford a brown oil. Purification of the crude product by flash column chromatography ($SiO_2$, Cyclohexane : EtOAc, 8:2) gave the *title compound* **S2** (1.57 g, 8.79 mmol, 88%) as a pale yellow oil; $R_f$ = 0.15 (Cyclohexane / EtOAc, 8:2); $^1$H NMR (400 MHz, $CDCl_3$) $\delta_H$ 9.80 (s, 1H), 7.95 (d, $J$ = 8.2 Hz, 2H), 7.25 (d, $J$ = 8.2 Hz, 2H), 3.89 (s, 3H), 2.99 (t, $J$ = 7.5 Hz, 2H), 2.80 (t, $J$ = 7.5 Hz, 2H); $^{13}$C NMR (101 MHz, $CDCl_3$) $\delta_C$ 200.9 (CH), 167.0 (C), 145.9 (C), 130.0 (CH), 128.4 (CH), 128.4 (C), 52.1 ($CH_3$), 44.9 ($CH_2$), 28.1 ($CH_2$). The analytical data are consistent with those reported in the literature.[10]

**Step 2: Synthesis of 3-(4-(Hydroxymethyl)phenyl)propan-1-ol (7a):** To a stirred solution of methyl 4-(3-oxopropyl)benzoate (**S2**, 5.00 mmol, 961 mg, 1.00 equiv.) in anhydrous THF (30 mL) under argon ay 0 °C was added dropwise $LiAlH_4$ in THF (1.0 M) (30 mL, 20.0 mmol, 4.00 equiv.), over 5 minutes. The mixture was allowed to warm to room temperature and stirred overnight. The mixture was then quenched with $H_2O$ (50 mL) and 1 M aq. NaOH (30 mL), and diluted with $Et_2O$ (100 ml). The organic phase was separated and the aqueous phase extracted with $Et_2O$ (3 × 50 mL). The combined organic extracts were dried ($MgSO_4$), filtered, and concentrated *in vacuo* to afford the crude product as a colourless oil. Purification of the crude product by flash column chromatography ($SiO_2$, Cyclohexane : EtOAc, 4:6) gave the *title compound* **7a** (710 mg, 4.22 mmol, 84%) as a colourless solid; m.p. 48–50 °C; $R_f$ = 0.16 (Cyclohexane / EtOAc, 6:4); $^1$H NMR (400 MHz, $CDCl_3$) $\delta_H$ 7.27 (d, $J$ = 7.9 Hz, 2H), 7.18 (d, $J$ = 7.9 Hz, 2H), 4.63 (s, 2H), 3.64 (t, $J$ = 6.5 Hz, 2H), 2.26 (t, $J$ = 7.6 Hz, 2H), 2.17 (s, OH), 1.90–1.83 (m, 2H), 1.77 (s, OH); $^{13}$C NMR (101 MHz, $CDCl_3$) $\delta_C$ 141.4 (C), 138.2 (C), 128.7 (CH),

127.3 (CH), 65.2 ($CH_2$), 62.2 ($CH_2$), 34.2 ($CH_2$), 31.8 ($CH_2$). The analytical data are consistent with those reported in the literature.[11]

**4-(4-(Hydroxymethyl)phenyl)butan-2-ol (7b)**

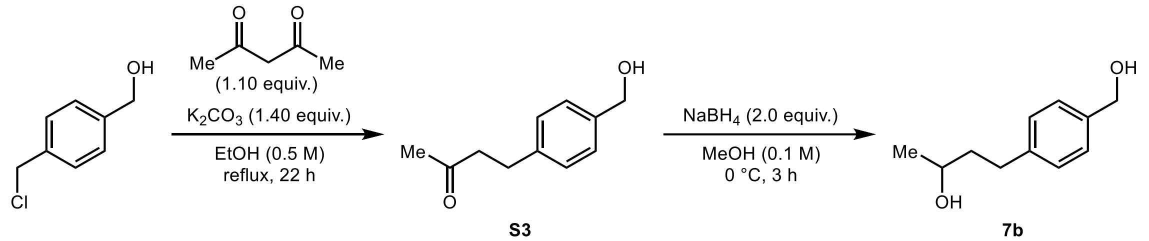


**Step 1: Synthesis of 4-(4-(Hydroxymethyl)phenyl)butan-2-one (S3)[11]:** A stirred solution of 4-(chloromethyl)benzyl alcohol (783 mg, 5.00 mmol, 1.00 equiv.), acetylacetone (1.13 mL, 5.50 mmol, 1.10 equiv.) and $K_2CO_3$ (967 mg, 7.00 mmol, 1.40 equiv.) in ethanol (10 mL) was stirred at reflux for 22 h. After cooling to rt, the mixture was filtered *in vacuo* and concentrated. The resulting residue was diluted with $H_2O$ (50 mL), then extracted with $Et_2O$ (3 × 30 mL). The combined organic extracts were dried ($MgSO_4$), filtered, concentrated *in vacuo* to afford the crude product as a colourless oil. Purification of the crude product by flash column chromatography ($SiO_2$, Cyclohexane : EtOAc, 7:3) gave the *title compound* **S3** (460 mg, 2.58 mmol, 52%) as a colourless oil; $R_f$ = 0.09 (Cyclohexane / EtOAc, 7:3); $^1H$ NMR (400 MHz, $CDCl_3$) $\delta_H$ 7.27 (d, $J$ = 8.1 Hz, 2H), 7.17 (d, $J$ = 8.1 Hz, 2H), 4.63 (s, 2H), 2.88 (t, $J$ = 7.6 Hz, 2H), 2.74 (t, $J$ = 7.6 Hz, 2H), 2.13 (s, 3H); $^{13}C$ NMR (101 MHz, $CDCl_3$) $\delta_C$ 208.1 (C), 140.6 (C), 138.9 (C), 128.6 (CH), 127.4 (CH), 65.2 ($CH_2$), 45.2 ($CH_3$), 30.2 ($CH_2$), 29.5 ($CH_3$). The analytical data are consistent with those reported in the literature.[11]

**Step 2: Synthesis of 4-(4-(Hydroxymethyl)phenyl)butan-2-ol (7b):** To a stirred solution of 4-(4-(hydroxymethyl)phenyl)butan-2-one (**S3**, 357 mg, 2.00 mmol, 1.00 equiv.), in methanol (20 mL) at 0 °C was added sodium borohydride (151 mg, 4.00 mmol, 2.00 equiv.). The mixture was stirred at 0 °C for 3 hours, then quenched with $H_2O$ (5 mL). The mixture was concentrated *in vacuo*, then diluted with $H_2O$ (25 mL) and $Et_2O$ (25 mL). The organic phase was separated, and the aqueous phase extracted with $Et_2O$ (3 × 25 mL). The combined organic extracts were dried ($MgSO_4$), filtered, and concentrated *in vacuo* to afford the crude product as a pale yellow oil. Purification of the crude product by flash column chromatography ($SiO_2$, Cyclohexane : EtOAc, 4:6) gave the *title compound* **7b** (460 mg, 1.28 mmol, 64%) as a colourless oil; $R_f$ = 0.22 (Cyclohexane / EtOAc, 6:4); $\nu_{max}/cm^{-1}$ (ATR) 3316, 2964, 2924, 2860, 1514; $^1H$ NMR (400 MHz, $CDCl_3$) $\delta_H$ 7.28 (d, $J$ = 7.8 Hz, 2H), 7.19 (d, $J$ = 7.8 Hz, 2H), 4.65 (s, 2H), 3.85–3.78 (m, 1H), 2.77–2.65 (m, 2H) 1.82–1.69 (m, 3H), 1.22 (d, $J$ = 6.2 Hz, 3H); $^{13}C$ NMR (101 MHz, $CDCl_3$) $\delta_C$ 141.7 (C), 138.6 (C), 128.7 (CH), 127.4 (CH), 67.6 (CH), 65.3 ($CH_2$), 40.9 ($CH_2$), 31.9 ($CH_2$), 23.8 ($CH_3$); HRMS (ESI) [M+$Na^+$] calculated for $C_{11}H_{16}NaO_2$: 203.1043, found: 203.1038 (2.20 ppm error).

**1-Phenylbutane-1,4-diol (9)**[12]

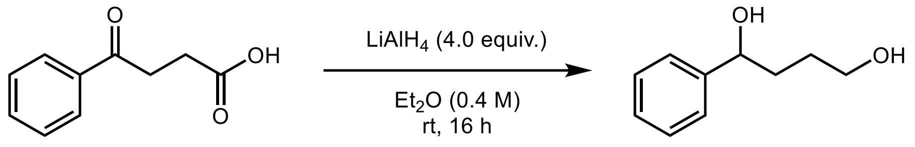


9

To a suspension of lithium aluminium hydride (1.52 g, 40.0 mmol, 4.00 equiv.) in $Et_2O$ (100 mL) under argon was added 3-benzoylpropionic acid (1.78 g, 10.0 mmol, 1.00 equiv.) as a solution in $Et_2O$, dropwise over 2 minutes. The mixture was stirred at room temperature for 16 hours. The reaction mixture was quenched with $H_2O$ (200 mL), and precipitates were removed by vacuum filtration. The mixture was extracted with EtOAc (3 × 50 mL), and the combined organic phases were dried ($MgSO_4$), filtered, and concentrated *in vacuo* to afford the crude product as a colourless oil. Purification of the crude product by flash column chromatography ($SiO_2$, Cyclohexane : EtOAc, 8:2 → 4:6) gave the *title compound* **9** (1.03 g, 6.20 mmol, 62%) which crystallised overnight to afford a colourless microcrystalline solid; m.p. 65–69 °C (lit. 65–67 °C)[13]; $R_f$ = 0.19 (Cyclohexane / EtOAc, 3:7); $^1$H NMR (400 MHz, $CDCl_3$) $\delta_H$ 7.36–7.32 (m, 4H), 7.29–7.25 (m, 1H), 4.73 (t, *J* = 6.3 Hz, 1H), 3.68 (app. p, *J* = 5.2 Hz, 1H), 2.59 (s, 1H), 2.07 (s, 1H), 1.87 (q, *J* = 7.0 Hz, 2H), 1.76–1.64 (m, 2H); $^{13}$C NMR (101 MHz, $CDCl_3$) $\delta_C$ 144.8 (C), 128.6 (CH), 127.6 (C), 125.9 (CH), 74.5 (CH), 62.9 ($CH_2$), 36.4 ($CH_2$), 29.3 ($CH_2$). The analytical data are consistent with those reported in the literature.[14]

**([1,1'-Biphenyl]-4-ylmethyl)triphenylphosphonium bromide (S4)**

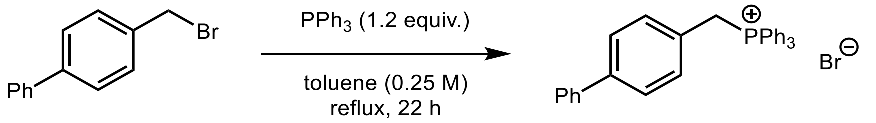


S4

Prepared according to conditions described in the literature.[15] A solution of 4-bromomethylbiphenyl (1.24 g, 5.00 mmol) and triphenylphosphine (1.38 g, 6.00 mmol, 1.20 equiv.) in toluene (20 mL) was stirred at reflux for 22 hours. The resulting precipitate was collected and washed with toluene to remove excess triphenylphosphine, then dried *in vacuo*. Trituration of the crude material with diethyl ether afforded the *title compound* **S4** (2.14 g, 4.20 mmol, 84%); m.p. 295–298 °C; $\nu_{max}$/cm$^{-1}$ (ATR) 3031, 2986, 2862, 2774, 1519, 1450, 1111; $^1$H NMR (400 MHz, $CDCl_3$) $\delta_H$ 7.80–7.74 (m, 9H), 7.64–7.60 (m, 6H), 7.47 (d, *J* = 7.34 Hz, 2H), 7.40–7.30 (m, 5H), 7.19 (dd, *J* = 8.3, 2.6 Hz, 2H), 5.51 (d, *J* = 14.4 Hz, 2H); $^{13}$C NMR (101 MHz, $CDCl_3$) $\delta_C$ 141.1 (d, *J* = 4 Hz, C), 140.1 (d, *J* = 2 Hz, C), 135.1 (d, *J* = 3 Hz, CH), 134.6 (d, *J* = 10 Hz, CH), 132.2 (d, *J* = 6 Hz, CH), 130.3 (d, *J* = 13 Hz, CH), 129.0 (CH), 127.8 (CH), 127.4 (d, *J* = 4 Hz, CH), 127.0 (CH), 126.3 (d, *J* = 9 Hz, C), 118.1 (d, *J* = 86 Hz, C), 30.7 (d, *J* = 47 Hz, $CH_2$); $^{31}$P NMR (162 MHz, $CDCl_3$) $\delta_P$ 23.0; HRMS (ESI) [M–Br]$^+$ calculated for $C_{31}H_{26}P^+$: 429.1767, found: 429.1764 (0.60 ppm error).

**([1,1'-Biphenyl]-4-ylmethyl)triphenylphosphonium hexafluorophosphate (S5)**

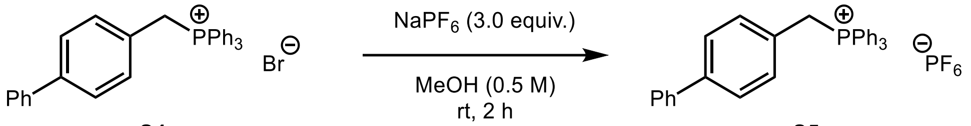


S4 S5

Prepared according to conditions described in the literature.[15] To a solution of ([1,1'-biphenyl]-4-ylmethyl)triphenylphosphonium bromide (**S4**, 1.53 g, 3.00 mmol) in methanol (6 mL) was added portionwise sodium hexafluorophosphate (1.51 g, 9.00 mmol, 3.00 equiv.). The reaction mixture was stirred at room temperature for 2 hours. The solvent was removed *in vacuo*, then the resulting precipitate was collected and washed with water, then dried *in vacuo*. Trituration of the crude material with diethyl ether afforded the *title compound* **S5** (1.53 g, 2.66 mmol, 89%); m.p. 205–206 °C (lit. 283–287 °C)[16]; $\nu_{max}/cm^{-1}$ (ATR) 2980, 1438, 1113, 828; $^1$H NMR (400 MHz, $CDCl_3$) $\delta_H$ 7.78 (td, *J* = 7.5, 1.8 Hz, 3H), 7.62 (td, *J* = 7.8, 3.5 Hz, 6H), 7.55–7.46 (m, 8H), 7.41–7.31 (m, 5H), 6.97 (dd, *J* = 8.4, 2.5 Hz, 2H); $^{13}$C NMR (101 MHz, $CDCl_3$) $\delta_C$ 141.6 (d, *J* = 4 Hz, C), 139.8 (d, *J* = 2 Hz, C), 135.5 (d, *J* = 3 Hz, CH), 134.2 (d, *J* = 10 Hz, CH), 131.7 (d, *J* = 5 Hz, CH), 130.5 (d, *J* = 13 Hz, CH), 129.0 (CH), 128.0 (CH), 127.7 (d, *J* = 3 Hz, CH), 125.4 (d, *J* = 9 Hz, C), 117.3 (d, *J* = 86 Hz, C), 30.3 (d, *J* = 49 Hz, $CH_2$); $^{31}$P NMR (162 MHz, $CDCl_3$) $\delta_P$ 22.3 (s), −144.2 (hept, *J* = 713 Hz); $^{19}$F NMR (376 MHz, $CDCl_3$) $\delta_F$ −72.4 (d, *J* = 713 Hz); HRMS (ESI) $[M–PF_6]^+$ calculated for $C_{31}H_{26}P^+$: 429.1767, found: 429.1762 (1.10 ppm error).

# 6. Deoxygenation of Benzylic Alcohols

**4-Methylbiphenyl (2a)**

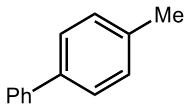


**2a**

Synthesised according to General Procedure B using 4-biphenylmethanol (184 mg, 1.00 mmol) as the starting material. Purification of the crude product by flash column chromatography ($SiO_2$, Cyclohexane) gave the *title compound* **2a** (134 mg, 795 µmol, 80%) as a colourless crystalline solid; m.p. 47–48 °C (lit. 45–47 °C)[17]; $R_f$ = 0.24 (Cyclohexane); $^1$H NMR (101 MHz, $CDCl_3$) $\delta_H$ 7.58 (dd, $J$ = 8.2, 1.3 Hz, 2H), 7.50 (d, $J$ = 8.2 Hz, 2H), 7.45–7.41 (m, 2H), 7.32 (t, $J$ = 7.4 Hz, 1H), 7.27–7.25 (m, 2H) 2.40 (s, 3H); $^{13}$C NMR (101 MHz, $CDCl_3$) $\delta_C$ 141.3 (C), 138.5 (C), 137.2 (C), 129.6 (CH), 128.9 (CH), 127.1 (CH), 127.1 (CH), 127.1 (CH), 21.3 ($CH_3$). The analytical data are consistent with those reported in the literature.[18]

**4-Methylbenzonitrile (2b)**

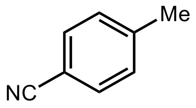


**2b**

Synthesised according to General Procedure B using 4-(hydroxymethyl)benzonitrile (133 mg, 1.00 mmol) as the starting material. A yield of 69% was determined by GC–MS analysis using dodecane as an external standard. EI-MS $[M]^+$ calculated for $C_8H_7N^+$: 117.1, found: 117.1. Retention time: 11.99 min. A yield of 74% was determined by $^1$H NMR spectroscopic analysis using 1,3,5-trimethoxybenzene as an internal standard, in relation to methyl resonance of the target compound at 2.36 ppm (s, 3H) in $CDCl_3$ (400 MHz), this value is consistent with those reported in the literature.[19]

**Methyl 4-methylbenzoate (2c)**

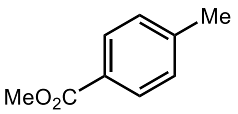


**2c**

Synthesised according to General Procedure B using methyl (4-hydroxymethyl)benzoate (166 mg, 1.00 mmol) as the starting material. A yield of 78% was determined by GC–MS analysis using dodecane as an external standard. EI-MS $[M]^+$ calculated for $C_9H_{10}O_2^+$: 150.1, found: 150.1. Retention time: 13.29 min. A yield of 72% was determined by $^1$H NMR spectroscopic analysis using 1,3,5-trimethoxybenzene

as an internal standard, in relation to methyl resonance of the target compound at 2.38 ppm (s, 3H) in $CDCl_3$ (400 MHz), this value is consistent with those reported in the literature.[19]

**1-Methylnaphthalene (2d)**

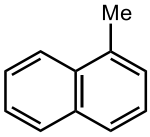

**2d**

Synthesised according to General Procedure B using 1-naphthalenemethanol (158 mg, 1.00 mmol) as the starting material. Purification of the crude product by flash column chromatography ($SiO_2$, Cyclohexane) gave the *title compound* **2d** (76.4 mg, 0.54 mmol, 54%) as a colourless oil; $R_f$ = 0.27 (Cyclohexane); $^1H$ NMR (400 MHz, $CDCl_3$) $\delta_H$ 8.03 (d, $J$ = 8.1 Hz, 1H), 7.88 (d, $J$ = 7.7 Hz, 1H), 7.74 (d, $J$ = 8.1 Hz, 1H), 7.57–7.49 (m, 2H), 7.43–7.39 (m, 1H), 7.35 (d, $J$ = 7.0 Hz, 1H), 2.73 (s, 3H); $^{13}C$ NMR (101 MHz, $CDCl_3$) $\delta_C$ 134.4 (C), 133.7 (C), 132.8 (C), 128.7 (CH), 126.7 (CH), 126.5 (CH), 125.9 (CH), 125.7 (CH), 125.7 (CH), 124.3 (CH), 19.5 ($CH_3$). The analytical data are consistent with those reported in the literature.[20]

**4-Methylanisole (2e)**

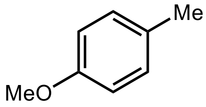

**2e**

Synthesised according to General Procedure B using 4-methoxybenzyl alcohol (138 mg, 1.00 mmol) as the starting material. A yield of 80% was determined by GC–MS analysis using dodecane as an external standard. EI-MS $[M]^+$ calculated for $C_8H_{10}O^+$: 122.1, found: 122.1. Retention time: 9.95 min. A yield of 80% was determined by $^1H$ NMR spectroscopic analysis using 1,3,5-trimethoxybenzene as an internal standard, in relation to methyl resonance of the target compound at 2.22 ppm (s, 3H) in $CDCl_3$ (400 MHz), this value is consistent with those reported in the literature.[19]

***tert*-Butyl *p*-tolylcarbamate (2f)**

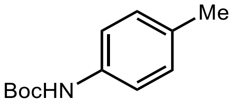

**2f**

Synthesised according to General Procedure B using *tert*-butyl (4-(hydroxymethyl)phenyl)carbamate (223 mg, 1.00 mmol) as the starting material. Purification of the crude product by flash column chromatography ($SiO_2$, Cyclohexane : EtOAc, 1:0→19:1) gave the *title compound* **2f** (108 mg, 0.52

mmol, 52%) as a colourless solid; m.p. 81–83 °C (lit. 84–85 °C)[21]; $R_f$ = 0.29 (Cyclohexane / EtOAc, 9:1); $^1$H NMR (400 MHz, $CDCl_3$) $\delta_H$ 7.23 (d, $J$ = 7.2 Hz, 2H), 7.09 (d, $J$ = 8.2 Hz, 2H), 6.40 (s, 1H), 2.29 (s, 3H), 1.51 (s, 9H); $^{13}$C NMR (101 MHz, $CDCl_3$) $\delta_C$ 153.0 (C), 135.9 (C), 132.7 (C), 129.6 (CH), 118.8 (CH), 80.4 (C), 28.5 ($CH_3$), 20.9 ($CH_3$). The analytical data are consistent with those reported in the literature.[22]

**4-*tert*-Butyltoluene (2g)**

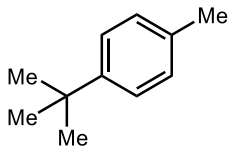


**2g**

Synthesised according to General Procedure B using 4-*tert*-butylbenzyl alcohol (164 mg, 1.00 mmol) as the starting material. A yield of 59% was determined by GC–MS analysis using tetradecane as an internal standard. EI-MS $[M]^+$ calculated for $C_{11}H_{16}^+$: 148.1, found: 148.1. Retention time: 10.24 min. A yield of 60% was determined by $^1$H NMR spectroscopic analysis using 1,3,5-trimethoxybenzene as an internal standard, in relation to methyl resonance of the target compound at 2.26 ppm (s, 3H) in $CDCl_3$ (400 MHz), this value is consistent with those reported in the literature.[19]

Also synthesised according to General Procedure B using 4-*tert*-butylbenzyl mercaptan (188 µL, 1.00 mmol) as the starting material. A yield of 66% was determined by GC–MS analysis using tetradecane as the external standard. EI-MS $[M]^+$ calculated for $C_{11}H_{16}^+$: 148.1, found: 148.1. Retention time: 10.26 min. A yield of 70% was determined by $^1$H NMR spectroscopic analysis using 1,3,5-trimethoxybenzene as an internal standard, in relation to methyl resonance of the target compound at 2.26 ppm (s, 3H) in $CDCl_3$ (400 MHz), this value is consistent with those reported in the literature.[19]

**4-Fluorotoluene (2h)**

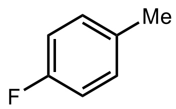


**2h**

Synthesised according to General Procedure B using 4-fluorobenzyl alcohol (126 mg, 1.00 mmol) as the starting material. A yield of 78% was determined by $^1$H NMR spectroscopic analysis using 1,3,5-trimethoxybenzene as an internal standard, in relation to methyl resonance of the target compound at 2.24 ppm (s, 3H) in $CDCl_3$ (400 MHz), this value is consistent with those reported in the literature.[23]

**4-Chlorotoluene (2i)**

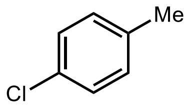

**2i**

Synthesised according to General Procedure B using 4-chlorobenzyl alcohol (143 mg, 1.00 mmol) as the starting material and anhydrous DCM as the solvent. A yield of 79% was determined by GC–MS analysis using dodecane as an external standard. EI-MS $[M]^+$ calculated for $C_7H_7Cl^+$: 126.0, found: 126.0. Retention time: 8.42 min. A yield of 73% was determined by $^1H$ NMR spectroscopic analysis using 1,3,5-trimethoxybenzene as an internal standard, in relation to methyl resonance of the target compound at 2.31 ppm (s, 3H) in $CDCl_3$ (400 MHz), this value is consistent with those reported in the literature.[24]

**4-Bromotoluene (2j)**

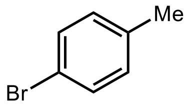

**2j**

Synthesised according to General Procedure B using 4-bromobenzyl alcohol (187 mg, 1.00 mmol) as the starting material and anhydrous DCM as the solvent. A yield of 64% was determined by GC–MS spectroscopic analysis using tetradecane as an internal standard. EI-MS $[M]^+$ calculated for $C_7H_7Br^+$: 170.0, found: 170.90. Retention time: 10.23 min. A yield of 60% was determined by $^1H$ NMR spectroscopic analysis using 1,3,5-trimethoxybenzene as an internal standard, in relation to methyl resonance of the target compound at 2.29 ppm (s, 3H) in $CDCl_3$ (400 MHz), this value is consistent with those reported in the literature.[24]

**1-Methyl-4-(trifluoromethyl)benzene (2k)**

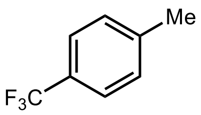

**2k**

Synthesised according to General Procedure B using 4-trifluoromethylbenzyl alcohol (176 mg, 1.00 mmol) as the starting material and anhydrous DCM as the solvent. A yield of 68% was determined by $^1H$ NMR spectroscopic analysis using 1,3,5-trimethoxybenzene an internal standard, in relation to methyl resonance of the target compound at 2.41 ppm (s, 3H) in $CDCl_3$ (400 MHz), this value is consistent with those reported in the literature.[24]

**4,4,5,5-Tetramethyl-2-(*p*-tolyl)-1,3,2-dioxaborolane (2l)**

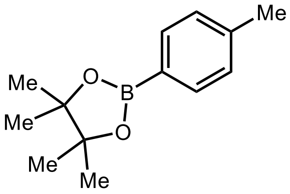

**2l**

Synthesised according to General Procedure B using (4-(4,4,5,5-tetramethyl-1,3,2-dioxaborolan-2-yl)phenyl)methanol (234 mg, 1.00 mmol) as the starting material. A yield of 77% was determined by GC–MS analysis using dodecane as an external standard. EI-MS $[M]^+$ calculated for $C_{13}H_{19}BO_2^+$: 218.2, found: 218.2. Retention time: 16.45 min. A yield of 91% was determined by $^1H$ NMR spectroscopic analysis using 1,3,5-trimethoxybenzene as an internal standard, in relation to methyl resonance of the target compound at 2.31 ppm (s, 3H) in $CDCl_3$ (400 MHz), this value is consistent with an authentic sample of **2l** (vide infra).

**3-(*m*-Tolyl)tetrahydrofuran (2o)**

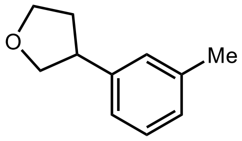

**2o**

Synthesised according to General Procedure B using (4-(tetrahydrofuran-3-yl)phenyl)methanol **1o** (178 mg, 1.00 mmol) as the starting material. Purification of the crude product by flash column chromatography ($SiO_2$, Cyclohexane / EtOAc, 19:1) gave the *title compound* **2o** (78.5 mg, 484 µmol, 48%) as a pale yellow oil; $R_f$ = 0.26 (Cyclohexane : EtOAc, 9:1); $\nu_{max}/cm^{-1}$ (ATR) 3022, 2968, 2926, 2859, 1056; $^1H$ NMR (400 MHz, $CDCl_3$) $\delta_H$ 7.26 (t, $J$ = 7.5 Hz, 1H), 7.13–7.08 (m, 3H), 4.19 (app. t, $J$ = 8.0 Hz, 1H), 4.12 (app. td, $J$ = 8.4, 4.5 Hz, 1H), 3.97 (app. q, $J$ = 7.9 Hz, 1H), 3.78 (app. t, $J$ = 8.0 Hz, 1H), 3.42 (app. p, $J$ = 7.9 Hz, 1H), 2.44–2.36 (m, 1H), 2.40 (s, 3H), 2.06 (app. dq, $J$ = 12.3, 8.2 Hz, 1H); $^{13}C$ NMR (101 MHz, $CDCl_3$) $\delta_C$ 142.7 (C), 138.3 (C), 128.6 (CH), 128.1 (CH), 127.3 (CH), 124.4 (CH), 74.8 ($CH_2$), 68.6 ($CH_2$), 45.0 (CH), 34.8 ($CH_2$), 21.6 ($CH_3$); HRMS (ES) $[M]^+$ calculated for $C_{11}H_{14}O$: 162.1039, found: 162.1039 (0.21 ppm error).

**5-Methylbenzo[d][1,3]dioxole (2p)**

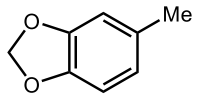

**2p**

Synthesised according to General Procedure B using benzo[d][1,3]dioxol-5-ylmethanol (152 mg, 1.00 mmol) as the starting material. A yield of 48% was determined by GC–MS analysis using dodecane as an external standard. EI-MS $[M]^+$ calculated for $C_8H_7O_2^+$: 135.0, found: 135.0. Retention time: 11.66 min. A yield of 56% was determined by $^1H$ NMR spectroscopic analysis using 1,3,5-trimethoxybenzene as an internal standard, in relation to methyl resonance of the target compound at 2.23 ppm (s, 3H) in $CDCl_3$ (400 MHz), this value is consistent with an authentic sample of **2p** (vide infra).

***N*-(*m*-Tolyl)cyclohexanecarboxamide (2q)**

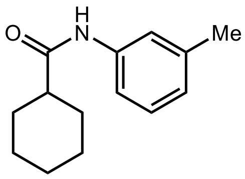

**2q**

Synthesised according to General Procedure B using *N*-(3-(hydroxymethyl)phenyl)cyclohexanecarboxamide (**2q**, 233 mg, 1.00 mmol) as the starting material. Purification of the crude product by flash column chromatography ($SiO_2$, Cyclohexane : EtOAc, 9:1) gave the *title compound* **2q** (78.5 mg, 484 μmol, 48%) as a colourless solid; m.p. 90–93°C; $R_f$ = 0.11 (Cyclohexane / EtOAc, 9:1); $\nu_{max}/cm^{-1}$ (ATR) 3253, 2927, 2851, 1659; $^1H$ NMR (400 MHz, $CDCl_3$) $\delta_H$ 7.42 (s, 1H), 7.28–7.17 (m, 2H), 7.10 (s, 1H), 6.91 (d, *J* = 7.5 Hz, 1H), 2.33 (s, 3H), 2.21 (tt, *J* = 12.0, 3.6 Hz, 1H), 1.95 (d, *J* = 13.0 Hz, 2H), 1.84 (app. dq, *J* = 13.7, 3.8 Hz, 2H), 1.72–1.69 (m, 1H), 1.54 (app. qd, *J* = 12.1 Hz, 3.1 Hz, 2H), 1.37–1.20 (m, 3H); $^{13}C$ NMR (101 MHz, $CDCl_3$) $\delta_C$ 174.6 (C), 139.0 (C), 138.2 (C), 128.8 (CH), 124.9 (CH), 120.6 (CH), 116.9 (CH), 46.6 ($CH_2$), 29.8 ($CH_2$), 25.8 ($CH_2$), 25.8 ($CH_2$), 21.6 ($CH_3$); HRMS (ESI) $[M+H]^+$ calculated for $C_{14}H_{20}NO$: 218.1539, found: 218.1534 (2.40 ppm error)].

***o*-Xylene (2s)**

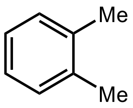

**2s**

Synthesised according to General Procedure B using 2-methylbenzyl alcohol (122 mg, 1.00 mmol) as the starting material. A yield of 77% was determined by GC–MS analysis using dodecane as an external

standard. EI-MS $[M]^+$ calculated for $C_8H_{10}^+$: 106.1, found: 106.1. Retention time: 7.04 min. A yield of 75% was determined by $^1$H NMR spectroscopic analysis using 1,3,5-trimethoxybenzene as an internal standard, in relation to methyl resonance of the target compound at 2.22 ppm (s, 3H) in $CDCl_3$ (400 MHz), this value is consistent with those reported in the literature.[24]

**4-Ethyl-4'-methyl-1,1'-biphenyl (2t)**

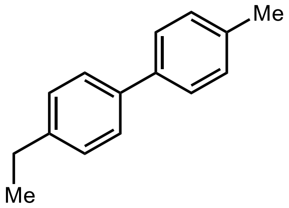


**2t**

Synthesised according to General Procedure B using 4-methyl-4'-vinyl-1,1'-biphenylmethanol (210 mg, 1.00 mmol) as the starting material. Purification of the crude product by flash column chromatography ($SiO_2$, Cyclohexane) gave the *title compound* **2t** (78.9 mg, 400 μmol, 40%) as a crystalline yellow solid, m.p. 58–62 °C (lit. 56–58 °C)[25]; $R_f$ = 0.25 (Cyclohexane); $^1$H NMR (400 MHz, $CDCl_3$) $\delta_H$ 7.52–7.48 (m, 4H), 7.28–7.23 (m, 4H), 2.70 (q, $J$ = 7.5 Hz, 2H), 2.40 (s, 3H), 1.38 (t, $J$ = 7.5 Hz, 3H); $^{13}$C NMR (101 MHz, $CDCl_3$) $\delta_C$ 143.1 (C), 138.6 (C), 138.4 (C) 136.7 (C) 129.4 (CH), 128.3 (CH), 126.9 (CH), 126.9 (CH), 28.5 ($CH_2$), 21.1 ($CH_3$), 15.6 ($CH_3$). The analytical data are consistent with those reported in the literature.[14]

**4-Ethylanisole (4a)**

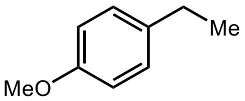


**4a**

Synthesised according to General Procedure B using 4-methoxy-α-methylbenzyl alcohol (152 mg, 1.00 mmol) as the starting material. A yield of 71% was determined by GC–MS analysis using dodecane as an external standard. EI-MS $[M]^+$ calculated for $C_9H_{12}O^+$: 136.1, found: 136.1. Retention time: 11.43 min. A yield of 71% was determined by $^1$H NMR spectroscopic analysis using 1,3,5-trimethoxybenzene as an internal standard, in relation to methyl resonance of the target compound at 2.53 ppm (q, $J$ = 7.9 Hz, 2H) in $CDCl_3$ (400 MHz), this value is consistent with those reported in the literature.[24]

**Ethylbenzene (4b)**

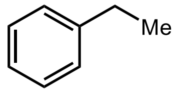


**4b**

Synthesised according to General Procedure B using 4-methoxy-α-methylbenzyl alcohol (122 mg, 1.00 mmol) as the starting material. A yield of 24% was determined by GC–MS analysis using dodecane as an external standard. EI-MS $[M]^+$ calculated for $C_8H_{10}^+$: 106.1, found: 106.1. Retention time: 6.35 min. A yield of 23% was determined by $^1$H NMR spectroscopic analysis using 1,3,5-trimethoxybenzene as an internal standard, in relation to methyl resonance of the target compound at 1.19 ppm (t, $J$ = 7.7 Hz 3H) in $CDCl_3$ (400 MHz), this value is consistent with those reported in the literature.[24]

**Methyl 4-ethylbenzoate (4c)**

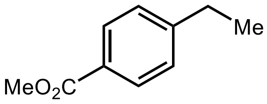


**4c**

Synthesised according to General Procedure B using methyl 4-(1-hydroxyethyl)benzoate (180 mg, 1.00 mmol) as the starting material. A yield of 41% was determined by GC–MS analysis using dodecane as an external standard. EI-MS $[M]^+$ calculated for $C_{10}H_{12}O_2^+$: 164.1, found: 164.1. Retention time: 14.57 min. A yield of 49% was determined by $^1$H NMR spectroscopic analysis using 1,3,5-trimethoxybenzene as an internal standard, in relation to methyl resonance of the target compound at 2.63 ppm (q, $J$ = 7.7 Hz, 2H) in $CDCl_3$ (400 MHz), this value is consistent with an authentic sample of **4c** (vide infra).

**4-Propyl-1,1'-biphenyl (4d)**

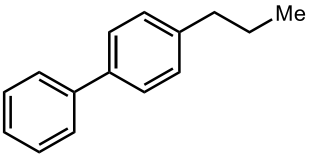


**4d**

Synthesised according to General Procedure B using 1-([1,1'-biphenyl]-4-yl)propan-1-ol (212 mg, 1.00 mmol) as the starting material. Purification of the crude product by flash column chromatography ($SiO_2$, Cyclohexane) gave the *title compound* **4d** (122 mg, 0.62 mmol, 62%) as a colourless oil; $R_f$ = 0.26 (Cyclohexane); $^1$H NMR (400 MHz, $CDCl_3$) $\delta_H$ 7.64 (d, $J$ = 8.4 Hz, 2H), 7.57 (d, $J$ = 7.9 Hz, 2H), 7.49–7.45 (m, 2H), 7.38–7.35 (m, 1H), 7.30 (d, $J$ = 7.9 Hz, 2H), 2.68 (t, $J$ = 7.7 Hz, 2H), 1.79–1.69 (m, 2H), 1.03 (t, $J$ = 7.4 Hz, 3H); $^{13}$C NMR (101 MHz, $CDCl_3$) $\delta_C$ 142.0 (C), 141.3 (C), 138.7 (C), 129.0 (CH), 128.8 (CH), 127.1 (CH), 127.1 (CH), 127.1 (CH), 37.8 ($CH_2$), 24.7 ($CH_2$), 14.0 ($CH_3$). The analytical data are consistent with those reported in the literature.[26]

**4-(Cyclopropylmethyl)-1,1'-biphenyl (4e)**

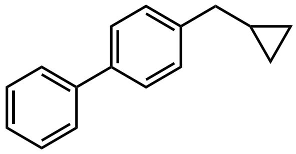

**4e**

Synthesised according to General Procedure B using [1,1'-biphenyl]-4-yl(cyclopropyl)methanol (**3e**, 224 mg, 1.00 mmol) as the starting material. Purification of the crude product by flash column chromatography ($SiO_2$, Cyclohexane) gave the *title compound* **4e** (174 mg, 0.84 mmol, 85%) as a colourless oil; $R_f = 0.21$ (Cyclohexane); $^1$H NMR (400 MHz, $CDCl_3$) $\delta_H$ 7.63 (d, $J = 7.7$ Hz, 2H), 7.56 (d, $J = 8.1$ Hz, 2H), 7.47 (t, $J = 7.4$ Hz, 2H), 7.40–7.34 (m, 3H) 2.64 (d, $J = 7.0$ Hz, 2H), 1.10–1.04 (m, 1H), 0.60 (d, $J = 6.8$ Hz, 2H), 0.29 (d, $J = 4.9$ Hz, 2H); $^{13}$C NMR (101 MHz, $CDCl_3$) $\delta_C$ 141.4 (C), 141.3 (C), 139.0 (C), 128.9 (CH), 128.9 (CH), 127.2 (CH), 127.2 (CH), 127.1 (CH), 40.1 ($CH_2$), 12.0 (CH), 4.9 ($CH_2$). The analytical data are consistent with those reported in the literature.[9]

**Indane (4f)**

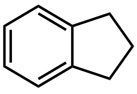

**4f**

Synthesised according to General Procedure B using 1-indanol (134 mg, 1.00 mmol) as the starting material. A yield of 29% was determined by GC–MS analysis using tetradecane as an internal standard. EI-MS $[M]^+$ calculated for $C_9H_{10}^+$: 118.1, found: 118.1. Retention time: 10.11 min. A yield of 32% was determined by $^1$H NMR spectroscopic analysis using 1,3,5-trimethoxybenzene as an internal standard, in relation to methyl resonance of the target compound at 2.85 ppm (t, $J = 7.4$ Hz, 4H) in $CDCl_3$ (400 MHz), this value is consistent with those reported in the literature.[24]

**Methyl 2-phenylacetate (4g)**

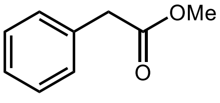


**4g**

Synthesised according to General Procedure B using methyl mandelate (166 mg, 1.00 mmol) as the starting material. Purification of the crude product by flash column chromatography ($SiO_2$, Cyclohexane : EtOAc, 1:0→19:1) gave the *title compound* **4g** (79.8 mg, 0.53 mmol, 53%) as a yellow oil; $R_f = 0.33$ (Cyclohexane / EtOAc, 9:1); $^1$H NMR (400 MHz, $CDCl_3$) $\delta_H$ 7.36–7.26 (m, 5H), 3.70 (s, 3H), 3.64 (s, 2H); $^{13}$C NMR (101 MHz, $CDCl_3$) $\delta_C$ 172.1 (C), 134.1 (C). 129.4 (CH), 128.7 (CH), 127.2 (CH), 52.2 ($CH_3$), 41.3 ($CH_2$). The analytical data are consistent with those reported in the literature.[27]

**Diphenylmethane (4h)**

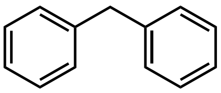

**4h**

Synthesised according to General Procedure B using diphenylmethanol (184 mg, 1.00 mmol) as the starting material. Purification of the crude product by flash column chromatography ($SiO_2$, Cyclohexane) gave the *title compound* **4h** (115 mg, 683 µmol, 68%) as a colourless oil; $R_f$ = 0.26 (Cyclohexane); $^1$H NMR (400 MHz, $CDCl_3$) $\delta_H$ 7.37–7.32 (m, 4H), 7.27–7.24 (m, 6H), 4.05 (s, 2H); $^{13}$C NMR (101 MHz, $CDCl_3$) $\delta_C$ 141.2 (C), 129.1 (CH), 128.6 (CH), 126.2 (CH), 42.1 ($CH_2$). The analytical data are consistent with those reported in the literature.[28]

**Triphenylmethane (4i)**

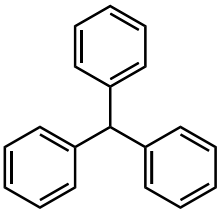

**4i**

Synthesised according to General Procedure B using triphenylmethanol (260 mg, 1.00 mmol) as the starting material. Purification of the crude product by flash column chromatography ($SiO_2$, Cyclohexane) gave the *title compound* **4i** (77.1 mg, 0.30 mmol, 36%) as a colourless solid; m.p. 92–94 °C (lit. 92–94 °C)[29]; $R_f$ = 0.25 (Cyclohexane); $^1$H NMR (400 MHz, $CDCl_3$) $\delta_H$ 7.30–7.25 (m, 6H), 7.22–7.16 (t, $J$ = 7.3 Hz, 3H), 7.12 (d, $J$ = 7.4 Hz, 6H), 5.55 (s, 1H); $^{13}$C NMR (101 MHz, $CDCl_3$) $\delta_C$ 144.1 (C), 129.6 (CH), 128.4 (CH), 126.4 (CH), 57.0 (CH). The analytical data are consistent with those reported in the literature.[30]

**3-(*p*-Tolyl)propan-1-ol (8a)**

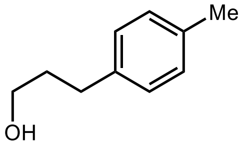


**8a**

Synthesised according to General Procedure B using 4-(4-(hydroxymethyl)phenyl)butan-2-ol (**7a**, 166 mg, 1.00 mmol) as the starting material. Purification of the crude product by flash column chromatography ($SiO_2$, Cyclohexane : EtOAc, 7:3) gave the *title compound* **8a** (78.6 mg, 480 mmol, 48%) as an orange oil; $R_f$ = 0.21 (Cyclohexane / EtOAc, 7:3); $^1$H NMR (400 MHz, $CDCl_3$) $\delta_H$ 7.10 (s, 4H), 3.68 (t, $J$ = 6.4 Hz, 2H), 2.67 (t, $J$ = 7.5 Hz, 2H), 2.32 (s, 3H), 1.92–1.85 (m, 2H); $^{13}$C NMR (101

MHz, $CDCl_3$) $\delta_C$ 138.8 (C), 135.4 (C), 129.2 (CH), 128.4 (CH), 62.4 ($CH_2$), 34.5 ($CH_2$), 31.7 ($CH_2$), 21.1 ($CH_3$). The analytical data are consistent with those reported in the literature.[31]

**4-(*p*-Tolyl)butan-2-ol (8b)**

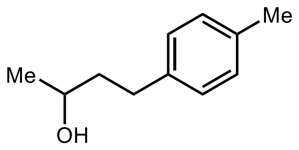

**8b**

Synthesised according to General Procedure B using 4-(4-(hydroxymethyl)phenyl)butan-2-ol (**7b**, 180 mg, 1.00 mmol) as the starting material. Purification of the crude product by flash column chromatography ($SiO_2$, Cyclohexane : EtOAc, 7:3) gave the *title compound* **8b** (78.6 mg, 0.48 mmol, 48%) as a colourless oil; $R_f$ = 0.13 (Cyclohexane / EtOAc, 7:3); $\nu_{max}/cm^{-1}$ (ATR) 3349, 2965, 2923, 2860, 1515; $^1$H NMR (400 MHz, $CDCl_3$) $\delta_H$ 7.10 (s, 4H), 3.86–3.77 (m, 1H), 2.76–2.60 (m, 2H), 2.32 (s, 3H), 1.82–1.69 (m, 2H), 1.23 (d, $J$ = 6.2 Hz, 3H); $^{13}$C NMR (101 MHz, $CDCl_3$) $\delta_C$ 139.1 (C), 135.3 (C), 129.2 (CH), 128.4 (CH), 67.2 (CH), 41.1 ($CH_2$), 31.8 ($CH_2$), 23.7 ($CH_3$), 21.1 ($CH_3$). HRMS (ESI) $[M+Na]^+$ calculated for $C_{11}H_{16}NaO$: 187.1093, found: 187.1092 (0.60 ppm error).

**4-Phenylbutan-1-ol (10) and 2-phenyltetrahydrofuran (11)**

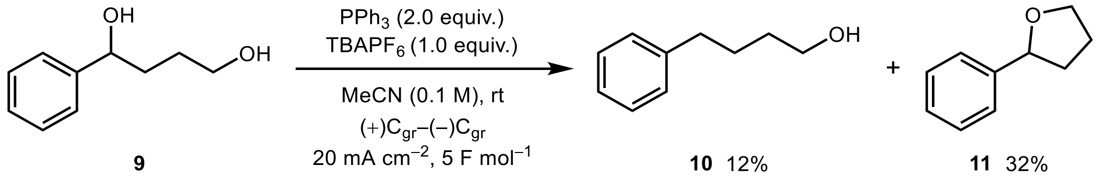


Synthesised according to General Procedure B using 1-phenylbutane-1,4-diol **9** (1.00 mmol, 166 mg) as the starting material. Purification of the crude products by flash column chromatography ($SiO_2$, Cyclohexane / EtOAc, 9:1) gave:

**4-Phenylbutan-1-ol (10)** (17.7 mg, 120 μmol, 12%) as a colourless oil; $R_f$ = 0.21 (Cyclohexane : EtOAc, 9:1); $^1$H NMR (400 MHz, $CDCl_3$) $\delta_H$ 7.30–7.17 (m, 2H), 7.20–7.17 (m, 3H), 3.67 (t, $J$ = 6.4 Hz, 2H), 2.65 (t, $J$ = 7.5 Hz, 2H), 1.73–1.58 (m, 4H); $^{13}$C NMR (101 MHz, $CDCl_3$) $\delta_C$ 142.5 (C), 128.5 (CH), 128.4 (CH), 125.9 (CH), 63.0 ($CH_2$), 35.8 ($CH_2$), 32.5 ($CH_2$), 27.7 ($CH_2$). The analytical data are consistent with those reported in the literature.[32]

**2-Phenyltetrahydrofuran (11)** (46.9 mg, 320 μmol, 32%) as a colourless oil; $R_f$ = 0.26 (Cyclohexane : EtOAc, 9:1); $^1$H NMR (400 MHz, $CDCl_3$) $\delta_H$ 7.36–7.32 (m, 4H), 7.27–7.24 (m, 1H), 4.91 (t, $J$ = 7.2 Hz, 1H), 4.11 (q, $J$ = 7.2 Hz, 1H), 3.95 (q, $J$ = 7.5 Hz, 1H), 2.38–2.30 (m, 1H), 2.08–1.95 (m, 2H), 1.87–1.78 (m, 1H); $^{13}$C NMR (101 MHz, $CDCl_3$) $\delta_C$ 143.6 (C), 128.4 (CH), 127.2 (CH), 125.7 (CH), 80.8 (CH), 68.8 ($CH_2$), 34.7 ($CH_2$), 26.1 ($CH_2$). The analytical data are consistent with those reported in the literature.[33]

# 7. E-Factor

E-Factor was calculated for synthesis of **2a** under the optimized conditions, at a 1 mmol scale:

**Table S1112.** Calculation of E-factor for the electrochemical deoxygenation reaction.

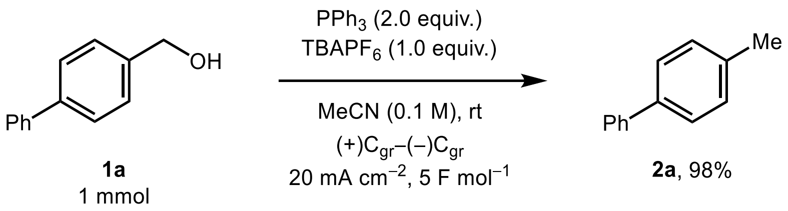


| | Amount / mmol | Molecular weight / g mol$^{-1}$ | Volume / mL | Density / g cm$^{-3}$ | Mass / mg |
|---|---|---|---|---|---|
| **1a** | 1.00 | 184.24 | - | - | 184.24 |
| $PPh_3$ | 2.00 | 287.29 | - | - | 524.58 |
| $TBAPF_6$ | 1.00 | 387.43 | - | - | 387.43 |
| MeCN | - | - | 10 | 0.786 | 7860 |
| MeCN (electrode washing) | - | - | 1 | 0.786 | 786 |
| silica | - | - | - | - | 10000 |
| cyclohexane | - | - | 80 | 0.779 | 62320 |
| Product **2a** | 0.98 | 168.23 | - | - | 164.87 |
| Total Mass of Materials | | | | | 82062.25 |
| Total Mass of Waste | | | | | 81897.38 |

$$E\ Factor = \frac{Mass\ of\ Waste\ (mg)}{Mass\ of\ Product\ (mg)} = \frac{81897.38}{164.87} = 496.73$$

$$E\ Factor\ (excluding\ purification) = \frac{Mass\ of\ Waste\ (mg)}{Mass\ of\ Product\ (mg)} = \frac{9577.38}{164.87} = 58.09$$

A comparative calculation was made based on the work of Doyle *et al.*,[34] under the assumption that purification would be carried out using the same chromatographic method:

**Table S13.** Calculation of E-factor for the photochemical deoxygenation reaction developed by Doyle *et al.*

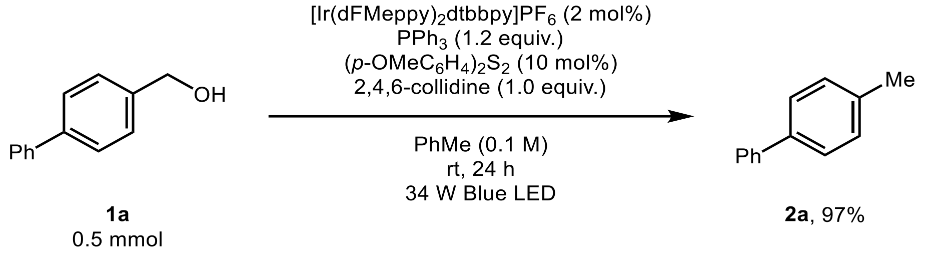


| | **Amount / mmol** | **Molecular weight / g mol$^{-1}$** | **Volume / mL** | **Density / g cm$^{-3}$** | **Mass / mg** |
|---|---|---|---|---|---|
| **1a** | 0.50 | 184.24 | - | - | 92.12 |
| [Ir] | 0.01 | 1013.97 | - | - | 10.14 |
| $PPh_3$ | 0.60 | 287.29 | - | - | 172.37 |
| (*p*-$OMeC_6H_4)_2S_2$ | 0.05 | 278.39 | - | - | 13.92 |
| 2,4,6-collidine | 0.50 | 121.18 | - | - | 60.59 |
| toluene | - | - | 5 | 0.867 | 4335 |
| silica | - | - | - | - | 5000 |
| cyclohexane | - | - | 40 | 0.779 | 31160 |
| Product **2a** | 0.49 | 168.23 | - | - | 81.59 |
| Total Mass of Materials | | | | | 40844.14 |
| Total Mass of Waste | | | | | 40762.55 |

$$E\ Factor = \frac{Mass\ of\ Waste\ (mg)}{Mass\ of\ Product\ (mg)} = \frac{40762.55}{81.59} = 499.60$$

$$E\ Factor\ (excluding\ purification) = \frac{Mass\ of\ Waste\ (mg)}{Mass\ of\ Product\ (mg)} = \frac{4602.55}{81.59} = 56.41$$

# 8. Preparation of GC–MS Standards

**4,4,5,5-Tetramethyl-2-(*p*-tolyl)-1,3,2-dioxaborolane (2l)**[35]

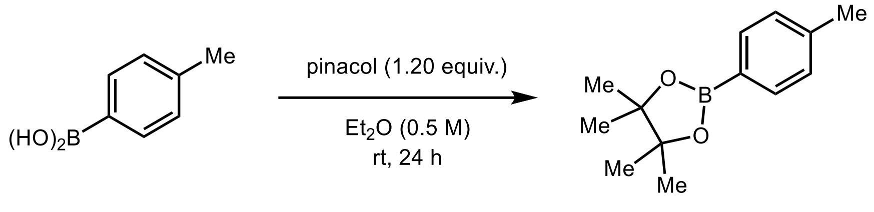


**2l**

To a stirred solution of 4-methylphenylboronic acid (408 mg, 3.00 mmol) in $Et_2O$ (6 mL) was added pinacol (428 mg, 3.60 mmol, 1.20 equiv.). The reaction was stirred at room temperature for 24 hours. The solvent was removed *in vacuo* and the crude product was purified by flash column chromatography ($SiO_2$, Pentane : $Et_2O$, 9:1) to afford the *title compound* **2l** (669 mg, 3.07 mmol, quant.) as a colourless crystalline solid; m.p. 54–56 °C (lit. 53–54 °C)[36]; $R_f$ = 0.51 (Pentane / $Et_2O$, 9:1); $^1H$ NMR (400 MHz, $CDCl_3$) $\delta_H$ 7.72 (d, $J$ = 7.6 Hz, 2H), 7.20 (d, $J$ = 7.6 Hz, 2H), 2.37 (s, 3H), 1.35 (s, 12H); $^{13}C$ NMR (101 MHz, $CDCl_3$) $\delta_C$ 141.5 (C), 135.0 (CH), 128.7 (CH), 83.8 (C), 25.0 ($CH_3$), 21.9 ($CH_3$); $^{11}B$ NMR (128 MHz, $CDCl_3$) 31.0. The analytical data are consistent with those reported in the literature.[35]

**5-Methylbenzo[d][1,3]dioxole (2p)**[37]

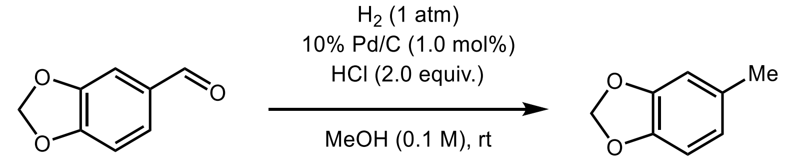


**2p**

To a stirred suspension of 10% Pd/C (106 mg, 100 μmol, 0.01 equiv.) and benzo[d][1,3]dioxole-5-carbaldehyde (1.50 g, 10.0 mmol) in methanol (100 mL) was added 37% HCl (136 μL, 5.00 mmol, 0.50 equiv.). The flask was evacuated and backfilled with hydrogen three times, then stirred at room temperature for 72 hours. The reaction vessel was purged with argon, and the Pd/C was removed by filtration through a short plug of celite. The solvent was removed *in vacuo* and the crude product was diluted in DCM (100 mL). The solution was washed with brine (100 mL), dried ($MgSO_4$), filtered and concentrated *in vacuo* to afford the crude product as a brown oil. Purification of the crude product by flash column chromatography ($SiO_2$, Pentane) gave the *title compound* **2p** (479 mg, 3.52 mmol, 35%) as a colourless oil; $R_f$ = 0.14 (Pentane); $^1H$ NMR (400 MHz, $CDCl_3$) $\delta_H$ 6.77 (d, $J$ = 7.8 Hz, 1H), 6.66 (s, 1H), 6.61 (d, $J$ = 7.8 Hz, 1H), 5.91 (s, 2H), 2.28 (s, 3H); $^{13}C$ NMR (101 MHz, $CDCl_3$) $\delta_C$ 147.6 (C), 145.4 (C), 131.5 (C), 121.6 (CH), 109.6 (CH), 108.1 (CH), 100.7 ($CH_2$), 21.2 ($CH_3$). The analytical data are consistent with those reported in the literature.[37]

**Methyl 4-ethylbenzoate (4c)**

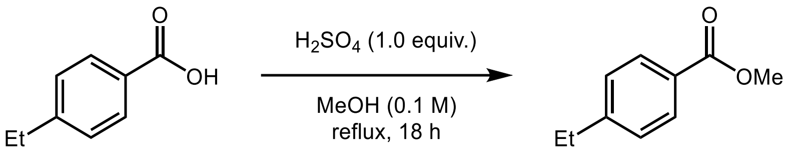


**4c**

To a stirred solution of 4-ethylbenzoic acid (1.50 g, 10.0 mmol) in methanol (100 mL) at reflux was added conc. $H_2SO_4$ (550 μL, 10.0 mmol, 1.00 equiv.), dropwise over 2 minutes. The reaction was stirred at reflux for 18 hours, then cooled to room temperature. The solvent was removed *in vacuo* and the crude product was diluted with EtOAc (100 mL). The solution was washed with sat. aq. $NaHCO_3$ (3 × 10 mL), dried ($MgSO_4$), filtered and concentrated in vacuo to afford the crude product as a pale yellow oil. Purification of the crude product by flash column chromatography ($SiO_2$, Cyclohexane : EtOAc, 9:1) gave the *title compound* **2c** (1.53 g, 9.34 mmol, 93%) as a colourless oil; $R_f$ = 0.43 (Cyclohexane / EtOAc, 9:1); $^1$H NMR (400 MHz, $CDCl_3$) $\delta_H$ 7.96 (d, $J$ = 8.4 Hz, 2H), 7.25 (d, $J$ = 8.4 Hz, 2H), 3.89 (s, 3H), 2.69 (q, $J$ = 7.7 Hz, 2H), 1.25 (t, $J$ = 7.7 Hz, 3H); $^{13}$C NMR (101 MHz, $CDCl_3$) $\delta_C$ 167.2 (C), 149.8 (C), 129.7 (CH), 128.0 (CH), 127.7 (CH), 52.0 ($CH_3$), 29.0 ($CH_2$), 15.3 ($CH_3$). The analytical data are consistent with those reported in the literature.[38]

# 9. Mechanistic Studies

**Deoxygenation of Substrates Used as Mechanistic Probes:**

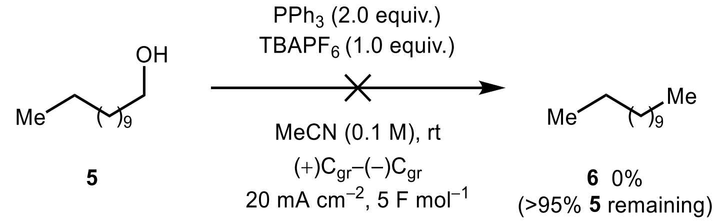


1-Dodecanol **5** (186 mg, 1.00 mmol) was subjected to the deoxygenation conditions according to General Procedure B. *n*-Dodecane (**6**) was not observed by $^1$H NMR or GC–MS analysis, and >5% consumption of the starting material was detected by $^1$H NMR spectroscopic analysis.

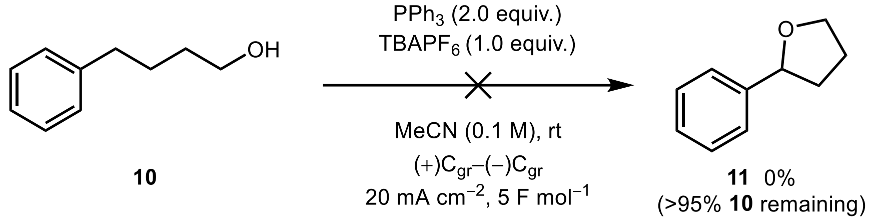


4-Phenylbutan-1-ol **10** (150 mg, 1.00 mmol) was subjected to the deoxygenation conditions according to General Procedure B. 2-Phenyltetrahydrofuran (**11**) was not observed by $^1$H NMR or GC–MS analysis.

**Electrochemical Reduction of Benzyltriphenylphosphonium Salt S5:**

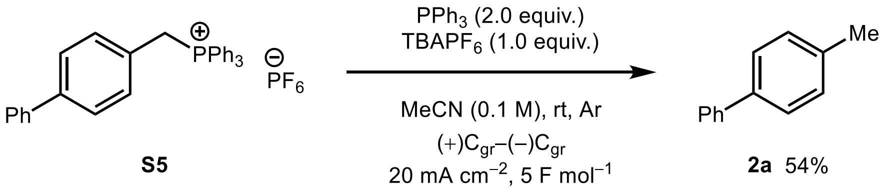


([1,1'-Biphenyl]-4-ylmethyl)triphenylphosphonium hexafluorophosphate (**S5**) (574 mg, 1.00 mmol) was subjected to the deoxygenation conditions according to General Procedure B. A yield of 66% was determined by GC–MS analysis using dodecane as an internal standard. EI-MS $[M]^+$ calculated for $C_{13}H_{12}^+$: 168.1, found: 168.1. Retention time: 17.32 min. A yield of 54% was determined by $^1$H-NMR spectroscopic analysis using 1,3,5-trimethoxybenzene as an internal standard, in relation to methyl resonance of the target compound at 2.33 ppm (s, 3H) in $CDCl_3$ (400 MHz), this value is consistent with an authentic sample of **2a** (vide supra).

**Cyclic Voltammetry**

Voltammetry experiments were performed on a CH Instruments 1140c potentiostat. 5mM analyte in 0.1 M $TBAPF_6$ / MeCN. Scan rate = 100 mV/s.

Working Electrode: 3 mm diameter glassy carbon disk. Counter Electrode: Pt Wire. Reference Electrode: 10 mM $Ag/AgNO_3$, 0.1 M $TBAPF_6$ / MeCN.

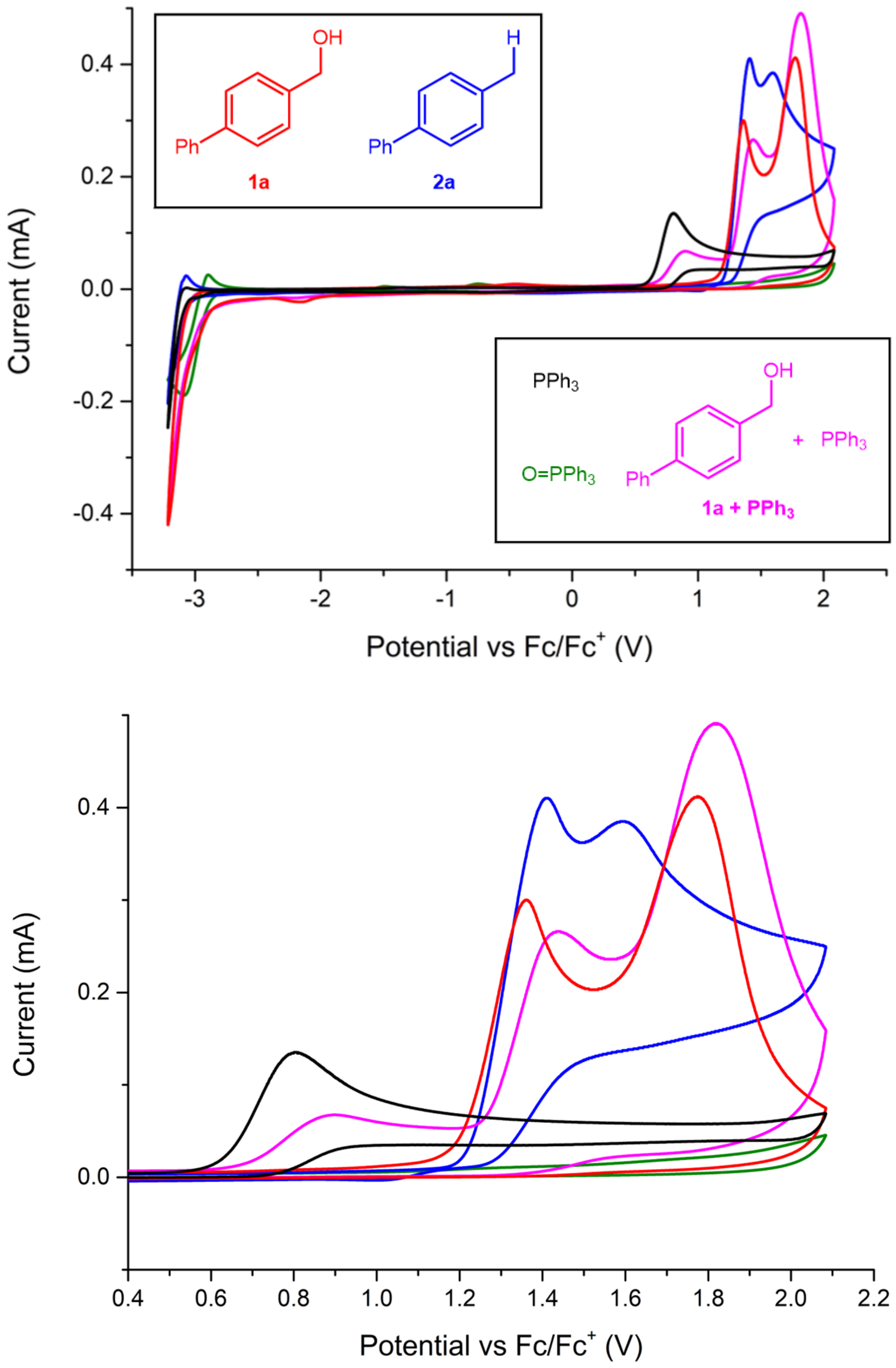


**Figure S4.** Cyclic voltammograms of triphenylphosphine (black) $E_{p,a}$ = +0.804 V vs Fc/Fc$^+$; biphenyl-4-methanol **1a** (red) $E_{p,a}$ = +1.361 V and +1.775 V vs Fc/Fc$^+$; triphenylphosphine and biphenyl-4-methanol **1a** (magenta) $E_{p,a}$ = +0.898 V, +1.439 V and +1.818 vs Fc/Fc$^+$; 4-methylbiphenyl **2a** (blue) $E_{p,a}$ +1.411 V and +1.594 V vs Fc/Fc$^+$; and triphenylphosphine oxide (green) $E_{p,c}$ = −3.093 V vs Fc/Fc$^+$. Upper: Full Voltammogram. Lower: Zoomed Voltammogram. Solvent = 0.1 M $TBAPF_6$ / MeCN, WE = GC, RE = 10 mM $Ag/AgNO_3$ / MeCN, CE = Pt, Scan Rate = 100 mV/s.

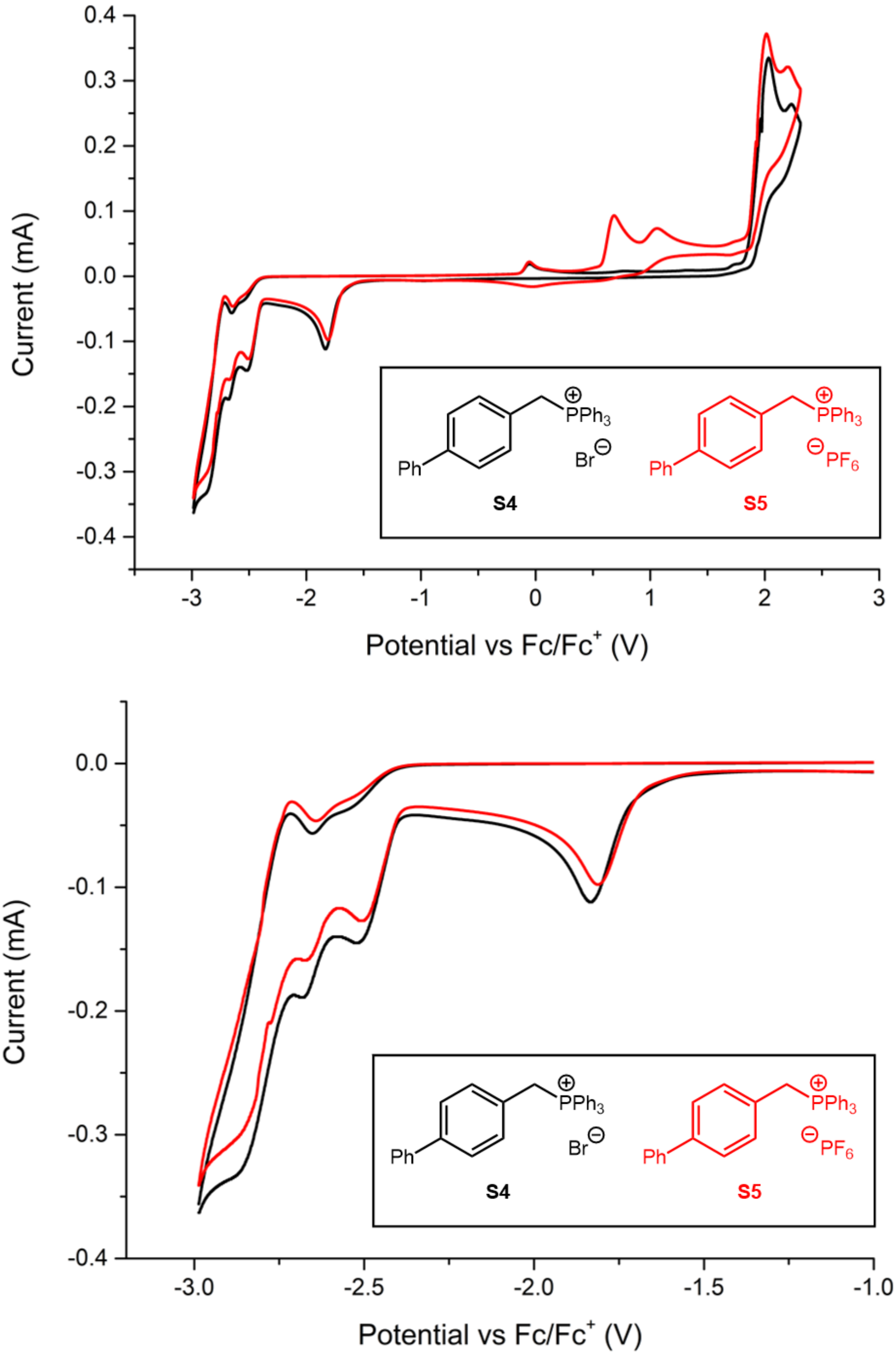


**Figure S5.** Cyclic voltammograms of ([1,1'-Biphenyl]-4-ylmethyl)triphenylphosphonium bromide **S4** (black, $E_{p,a} = +0.684$ and $+1.059$ V vs Fc/Fc$^+$, $E_{p,c} = -1.813$ V vs Fc/Fc$^+$) and ([1,1'-Biphenyl]-4-ylmethyl)triphenylphosphonium hexafluorophosphate **S5** (red, $E_{p,c} = -1.834$ V vs Fc/Fc$^+$). Upper: Full Voltammogram. Lower: Zoomed Voltammogram. Solvent = 0.1 M $TBAPF_6$ / MeCN, WE = GC, RE = 10 mM $Ag/AgNO_3$ / MeCN, CE = Pt, Scan Rate = 100 mV/s.

# 10. Deoxygenation of an Allylic Alcohol

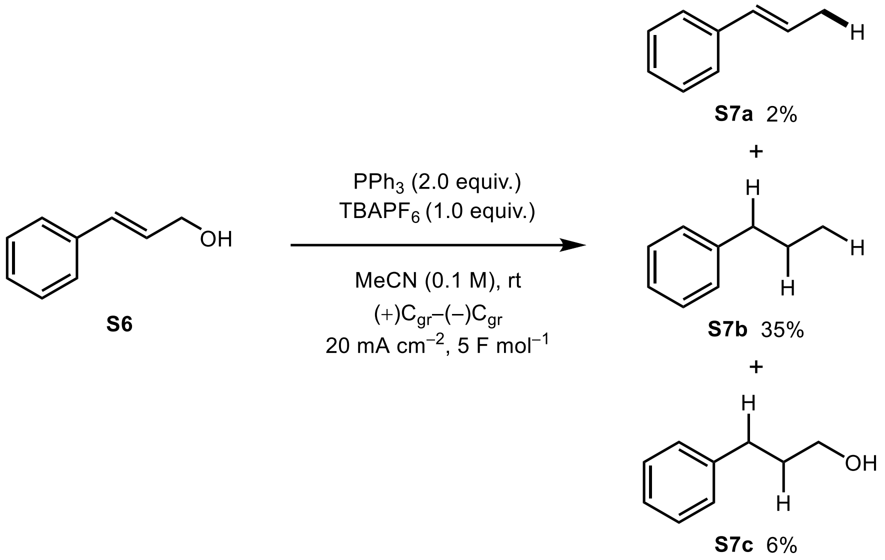


Cinnamyl alcohol (**S6**, 134 mg, 1.00 mmol) was subjected to the deoxygenation conditions according to General Procedure B. A yield of 2% *trans*-phenylpropene **S7a**, 35% *n*-propylbenzene **S7b** and 6% 3-phenyl-1-propanol **S7c** was determined by GC–MS analysis using dodecane as an internal standard. **S7a**: EI-MS $[M]^+$ calculated for $C_9H_{10}^+$: 118.1, found: 118.1. Retention time: 9.81 min. **S7b**: EI-MS $[M]^+$ calculated for $C_9H_{12}^+$: 120.1, found: 120.1. **S7c**: Retention time: 8.06 min. EI-MS $[M]^+$ calculated for $C_9H_{12}O^+$: 136.1, found: 136.1. Retention time: 13.89 min. The presence of *n*-Propylbenzene **S7b** was verified by the presence of characteristic peaks in $^1H$ NMR spectroscopic analysis (400 MHz, $CDCl_3$) $\delta_H$ 2.53 (t, $J$ = 7.6 Hz, 2H), 0.89 (t, $J$ = 7.4, 3H), these values are consistent with those reported in the literature.[39]

# 11. Computational Studies

All computational studies were carried out using Gaussian 16 Rev A.03.[40] The molecular geometries were optimized using PBE0-D3 functional[41, 42] using def2-TZVP basis set[43, 44] and SMD(acetonitrile) implicit solvent model[45]. Frequency calculations were performed on all structures and confirmed to contain no imaginary frequencies or just one imaginary frequency for ground states and transition states, respectively. Full set of DFT output files with optimized structures, frequencies and energies are available at the University of Nottingham Research Data Repository via: http://doi.org/10.17639/nott.7416.

This dataset contains Gaussian DFT output files of the key ground-states and transition state DFT optimized structures. The dataset contains 22 files in total. The data is organized in folders to match the reaction steps shown in Figure 1 in the manuscript. The folder structure and files in the dataset are shown below:

```
Computational data/
├── BnOH_pathway
│   ├── 1_SM
│   │   ├── BnOH_c0m1Afreq.out
│   │   ├── Ph3P_c0m1Afreq.out
│   │   └── Ph3P_c1m2Afreq.out
│   ├── 2_OHdeprot_TS
│   │   └── BnOHPh3Px2Ctzfreq.out
│   ├── 3_PhosphoranylRad
│   │   ├── BnOPh3P_c0m2Afreq.out
│   │   └── PPh3HAfreq.out
│   ├── 4_FragmentationTS
│   │   ├── BnOPh3P_c0m2Bfreq.out
│   │   └── PPh3HAfreq.out
│   └── 5_AlkylRad_P=O
```

```
│       ├── Bn_c0m2Afreq.out
│       ├── PPh3HAfreq.out
│       └── Ph3PO_c0m1Afreq.out
└── PrOH_pathway
    ├── 1_SM
    │   ├── Ph3P_c0m1Afreq.out
    │   ├── Ph3P_c1m2Afreq.out
    │   └── PrOH_c0m1Afreq.out
    ├── 2_OHdeprot_TS
    │   └── PrOHPh3Px2Ftzfreq.out
    ├── 3_PhosphoranylRad
    │   ├── PPh3HAfreq.out
    │   └── PrOPh3P_c0m2Afreq.out
    ├── 4_FragmentationTS
    │   ├── PPh3HAfreq.out
    │   └── PrOPh3P_c0m2Bfreq.out
    └── 5_AlkylRad_P=O
        ├── PPh3HAfreq.out
        ├── Ph3PO_c0m1Afreq.out
        └── Pr_c0m2Afreq.out
```

# 12. References


(1) Tian, C.; Meyer, T. H.; Stangier, M.; Dhawa, U.; Rauch, K.; Finger, L. H.; Ackermann, L. Cobaltaelectro-Catalyzed C-H Activation for Resource-Economical Molecular Syntheses. *Nat. Protoc.* **2020**, *15* (5), 1760-1774

(2) Fu, D.; Dong, J.; Du, H.; Xu, J. Methanesulfinylation of Benzyl Halides with Dimethyl Sulfoxide. *J. Org. Chem.* **2020**, *85* (4), 2752-2758

(3) Schadendorf, T.; Hoppmann, C.; Rück-Braun, K. Synthesis of Rigid Photoswitchable Hemithioindigo ω-Amino Acids. *Tetrahedron Lett.* **2007**, *48* (51), 9044-9047

(4) Byrne, T. J. M.; Mylrea, M. E.; Cuthbertson, J. D. A Redox-Relay Heck Approach to Substituted Tetrahydrofurans. *Org. Lett.* **2023**, *25* (13), 2361-2365

(5) Sellner, H.; Faber, C.; Rheiner, P. B.; Seebach, D. Immobilization of BINOL by Cross-Linking Copolymerization of Styryl Derivatives with Styrene, and Applications in Enantioselective Ti and Al Lewis Acid Mediated Additions of $Et_2Zn$ and $Me_3SiCN$ to Aldehydes and of Diphenyl Nitrone to Enol Ethers. *Chem. Eur. J.* **2000**, *6* (20), 3692-3705

(6) Hsu, S. F.; Plietker, B. Selective Transfer Hydrogenation and Hydrogenation of Ketones Using a Defined Monofunctional (P^N(Bn)^N(Bn)^P)-$Ru^{II}$ Complex. *Chem. Eur. J.* **2014**, *20* (15), 4242-4245

(7) Werner, T.; Riahi, A.; Schramm, H. Phosphonium Salt Catalyzed Addition of Diethylzinc to Aldehydes. *Synthesis* **2011**, *2011* (21), 3482-3490

(8) Hatano, M.; Miyamoto, M.; Ishihara, K. 3,3‘-Diphosphoryl-1,1‘-bi-2-naphthol−Zn(II) Complexes as Conjugate Acid−Base Catalysts for Enantioselective Dialkylzinc Addition to Aldehydes. *J. Org. Chem.* **2006**, *71* (17), 6474-6484

(9) Colombel, V.; Rombouts, F.; Oehlrich, D.; Molander, G. A. Suzuki Coupling of Potassium Cyclopropyl- and Alkoxymethyltrifluoroborates with Benzyl Chlorides. *J. Org. Chem.* **2012**, *77* (6), 2966-2970

(10) Wu, L. F.; Yao, J. W.; Zhang, X.; Liu, S. Y.; Zhuang, Z. N.; Wei, K. Pd-Catalyzed beta-C-H Arylation of Aldehydes and Ketones Based on a Transient Directing Group. *Org. Lett.* **2021**, *23* (16), 6237-6241

(11) Kuwano, S.; Harada, S.; Oriez, R.; Yamada, K. Chemoselective Conversion of alpha-Unbranched Aldehydes to Amides, Esters, and Carboxylic Acids by NHC-Catalysis. *Chem. Commun.* **2012**, *48* (1), 145-147

(12) Zhao, L. M.; Meng, Q. Y.; Fan, X. B.; Ye, C.; Li, X. B.; Chen, B.; Ramamurthy, V.; Tung, C. H.; Wu, L. Z. Photocatalysis with Quantum Dots and Visible Light: Selective and Efficient Oxidation of Alcohols to Carbonyl Compounds through a Radical Relay Process in Water. *Angew. Chem. Int. Ed.* **2017**, *56* (11), 3020-3024

(13) Estopiñá-Durán, S.; Donnelly, L. J.; McLean, E. B.; Hockin, B. M.; Slawin, A. M. Z.; Taylor, J. E. Aryl Boronic Acid Catalysed Dehydrative Substitution of Benzylic Alcohols for C-O Bond Formation. *Chem. Eur. J.* **2019**, *25* (15), 3950-3956

(14) Huy, P. H.; Koskinen, A. M. Efficient, Stereodivergent Access to 3-Piperidinols by Traceless $P(OEt)_3$ Cyclodehydration. *Org. Lett.* **2013**, *15* (20), 5178-5181

(15) Vikse, K. L.; Ahmadi, Z.; Manning, C. C.; Harrington, D. A.; McIndoe, J. S. Powerful Insight into Catalytic Mechanisms through Simultaneous Monitoring of Reactants, Products, and Intermediates. *Angew. Chem. Int. Ed.* **2011**, *50* (36), 8304-8306

(16) Nakasuji, K.; Akiyama, S.; Nakagawa, M. Linear Conjugated Systems Bearing Aromatic Terminal Groups. VIII. Syntheses and Electronic Spectra of Bis(4-biphenylyl)- and 2,2′-Difluorenylpoly-ynes. *Bull. Chem. Soc. Jpn.* **1972**, *45* (3), 883-891

(17) Barham, J. P.; Coulthard, G.; Emery, K. J.; Doni, E.; Cumine, F.; Nocera, G.; John, M. P.; Berlouis, L. E.; McGuire, T.; Tuttle, T.; Murphy, J. A. KOtBu: A Privileged Reagent for Electron Transfer Reactions? *J. Am. Chem. Soc.* **2016**, *138* (23), 7402-7410

(18) Li, H.; Sun, C. L.; Yu, M.; Yu, D. G.; Li, B. J.; Shi, Z. J. The Catalytic Ability of Various Transition Metals in the Direct Functionalization of Aromatic C-H Bonds. *Chem. Eur. J.* **2011**, *17* (13), 3593-3597

(19) Tao, C.; Liu, F.; Zhu, Y.; Liu, W.; Cao, Z. Copper-Catalyzed Aerobic Oxidative Synthesis of Aryl Nitriles from Benzylic Alcohols and Aqueous Ammonia. *Org. Biomol. Chem.* **2013**, *11* (20), 3349-3354

(20) Müller, V.; Ghorai, D.; Capdevila, L.; Messinis, A. M.; Ribas, X.; Ackermann, L. C-F Activation for $C(sp^2)$-$C(sp^3)$ Cross-Coupling by a Secondary Phosphine Oxide (SPO)-Nickel Complex. *Org. Lett.* **2020**, *22* (17), 7034-7040

(21) Sobhani, S.; Chahkamali, F. O.; Sansano, J. M. A New Bifunctional Heterogeneous Nanocatalyst for One-Pot Reduction-Schiff Base Condensation and Reduction-Carbonylation of Nitroarenes. *RSC Adv.* **2019**, *9* (3), 1362-1372

(22) Xiong, T.; Li, Y.; Lv, Y.; Zhang, Q. Remote Amide-Directed Palladium-Catalyzed Benzylic C-H Amination with N-Fluorobenzenesulfonimide. *Chem. Commun.* **2010**, *46* (36), 6831-6833

(23) Peng, Y.; Oestreich, M. $B(C_6F_5)_3$-Catalyzed Reductive Denitrogenation of Benzonitrile Derivatives. *Org. Lett.* **2022**, *24* (15), 2940-2943

(24) Deng, M.; Liu, K.; Yuan, S.; Luo, G.; Dian, L. Photoinduced $FeCl_3$-Catalyzed Chlorination of Aromatic Sulfonyl Chloride via Extrusion of $SO_2$ at Room Temperature. *Org. Lett.* **2023**, *25* (24), 4576-4580

(25) Luan, Y. X.; Zhang, T.; Yao, W. W.; Lu, K.; Kong, L. Y.; Lin, Y. T.; Ye, M. Amide-Ligand-Controlled Highly para-Selective Arylation of Monosubstituted Simple Arenes with Arylboronic Acids. *J. Am. Chem. Soc.* **2017**, *139* (5), 1786-1789

(26) Tsubouchi, A.; Muramatsu, D.; Takeda, T. Copper(I)-Catalyzed Alkylation of Aryl- and Alkenylsilanes Activated by Intramolecular Coordination of an Alkoxide. *Angew. Chem. Int. Ed.* **2013**, *52* (48), 12719-12722

(27) Zheng, Y.; Zhao, Y.; Tao, S.; Li, X.; Cheng, X.; Jiang, G.; Wan, X. Green Esterification of Carboxylic Acids Promoted by tert-Butyl Nitrite. *Eur. J. Org. Chem.* **2021**, *2021* (18), 2713-2718

(28) Bazyar, Z.; Hosseini-Sarvari, M. On/Off $O_2$ Switchable Photocatalytic Oxidative and Protodecarboxylation of Carboxylic Acids. *J. Org. Chem.* **2019**, *84* (21), 13503-13515

(29) Sai, M. An Efficient $Ga(OTf)_3$/Isopropanol Catalytic System for Direct Reduction of Benzylic Alcohols. *Adv. Synth. Catal.* **2018**, *360* (22), 4330-4335

(30) Kumar, G. G.; Laali, K. K. Facile Coupling of Propargylic, Allylic and Benzylic Alcohols with Allylsilane and Alkynylsilane, and their Deoxygenation with $Et_3SiH$, Catalyzed by $Bi(OTf)_3$ in [BMIM][$BF_4$] Ionic Liquid (IL), with Recycling and Reuse of the IL. *Org. Biomol. Chem.* **2012**, *10* (36), 7347-7355

(31) Cheung, F. K.; Lin, C.; Minissi, F.; Crivillé, A. L.; Graham, M. A.; Fox, D. J.; Wills, M. An Investigation into the Tether Length and Substitution Pattern of Arene-Substituted Complexes for Asymmetric Transfer Hydrogenation of Ketones. *Org. Lett.* **2007**, *9* (22), 4659-4662

(32) Harenberg, J. H.; Reddy Annapureddy, R.; Karaghiosoff, K.; Knochel, P. Continuous Flow Preparation of Benzylic Sodium Organometallics. *Angew. Chem. Int. Ed.* **2022**, *61* (30), e202203807

(33) Dilauro, G.; Cicco, L.; Vitale, P.; Perna, F. M.; Capriati, V. Ligand-Free Pd-Catalyzed Reductive Mizoroki-Heck Reaction Strategy for the One-Pot Synthesis of Functionalized Oxygen Heterocycles in Deep Eutectic Solvents. *Eur. J. Org. Chem.* **2022**, *26* (3), e202200814

(34) Stache, E. E.; Ertel, A. B.; Rovis, T.; Doyle, A. G. Generation of Phosphoranyl Radicals via Photoredox Catalysis Enables Voltage-Independent Activation of Strong C-O Bonds. *ACS Catal.* **2018**, *8* (12), 11134-11139

(35) Tan, P. W.; Haughey, M.; Dixon, D. J. Palladium(II)-Catalysed ortho-Arylation of N-Benzylpiperidines. *Chem. Commun.* **2015**, *51* (21), 4406-4409

(36) Wang, Z.; Wang, X.; Nishihara, Y. Nickel-Catalysed Decarbonylative Borylation of Aroyl Fluorides. *Chem. Commun.* **2018**, *54* (99), 13969-13972

(37) Xing, L.; Wang, X.; Cheng, C.; Zhu, R.; Liu, B.; Hu, Y. A Solvent-Controlled Highly Efficient Pd–C Catalyzed Hydrogenolysis of Benzaldehydes to Methylbenzenes via a Novel 'Acetal Pathway'. *Tetrahedron* **2007**, *63* (38), 9382-9386

(38) Zhang, D.; Iwai, T.; Sawamura, M. Iridium-Catalyzed Alkene-Selective Transfer Hydrogenation with 1,4-Dioxane as Hydrogen Donor. *Org. Lett.* **2019**, *21* (15), 5867-5872

(39) Carter, T. S.; Guiet, L.; Frank, D. J.; West, J.; Thomas, S. P. Iron-Catalysed Reduction of Olefins using a Borohydride Reagent. *Adv. Synth. Catal.* **2013**, *355* (5), 880-884

(40) *Gaussian 16 Rev. A.03*; Frisch, M. J.; Trucks, G. W.; Schlegel, H. B.; Scuseria, G. E.; Robb, M. A.; Cheeseman, J. R.; Scalmani, G.; Barone, V.; Petersson, G. A.; Nakatsuji, H.; Li, X.; Caricato, M.; Marenich, A. V.; Bloino, J.; Janesko, B. G.; Gomperts, R.; Mennucci, B.; Hratchian, H. P.; Ortiz, J. V.; Izmaylov, A. F.; Sonnenberg, J. L.; Williams; Ding, F.; Lipparini, F.; Egidi, F.; Goings, J.; Peng, B.; Petrone, A.; Henderson, T.; Ranasinghe, D.; Zakrzewski, V. G.; Gao, J.; Rega, N.; Zheng, G.; Liang, W.; Hada, M.; Ehara, M.; Toyota, K.; Fukuda, R.; Hasegawa, J.; Ishida, M.; Nakajima, T.; Honda, Y.;

Kitao, O.; Nakai, H.; Vreven, T.; Throssell, K.; Montgomery Jr., J. A.; Peralta, J. E.; Ogliaro, F.; Bearpark, M. J.; Heyd, J. J.; Brothers, E. N.; Kudin, K. N.; Staroverov, V. N.; Keith, T. A.; Kobayashi, R.; Normand, J.; Raghavachari, K.; Rendell, A. P.; Burant, J. C.; Iyengar, S. S.; Tomasi, J.; Cossi, M.; Millam, J. M.; Klene, M.; Adamo, C.; Cammi, R.; Ochterski, J. W.; Martin, R. L.; Morokuma, K.; Farkas, O.; Foresman, J. B.; Fox, D. J. Gaussian, Inc.: Wallingford, CT, 2016.

(41) Adamo, C.; Barone, V. Toward Reliable Density Functional Methods Without Adjustable Parameters: The PBE0 Model. *J. Chem. Phys.* **1999**, *110* (13), 6158-6170

(42) Grimme, S.; Antony, J.; Ehrlich, S.; Krieg, H. A Consistent and Accurate Ab Initio Parametrization of Density Functional Dispersion Correction (DFT-D) for the 94 Elements H-Pu. *J. Chem. Phys.* **2010**, *132* (15), 154104

(43) Weigend, F. Accurate Coulomb-Fitting Basis Sets for H to Rn. *Phys. Chem. Chem. Phys.* **2006**, *8* (9), 1057-1065

(44) Weigend, F.; Ahlrichs, R. Balanced Basis Sets of Split Valence, Triple Zeta Valence and Quadruple Zeta Valence Quality for H to Rn: Design and Assessment of Accuracy. *Phys. Chem. Chem. Phys.* **2005**, *7* (18), 3297-3305

(45) Marenich, A. V.; Cramer, C. J.; Truhlar, D. G. Universal Solvation Model Based on Solute Electron Density and on a Continuum Model of the Solvent Defined by the Bulk Dielectric Constant and Atomic Surface Tensions. *J. Phys. Chem. B* **2009**, *113* (18), 6378-6396

# 13. NMR Spectra of Substrates and Products

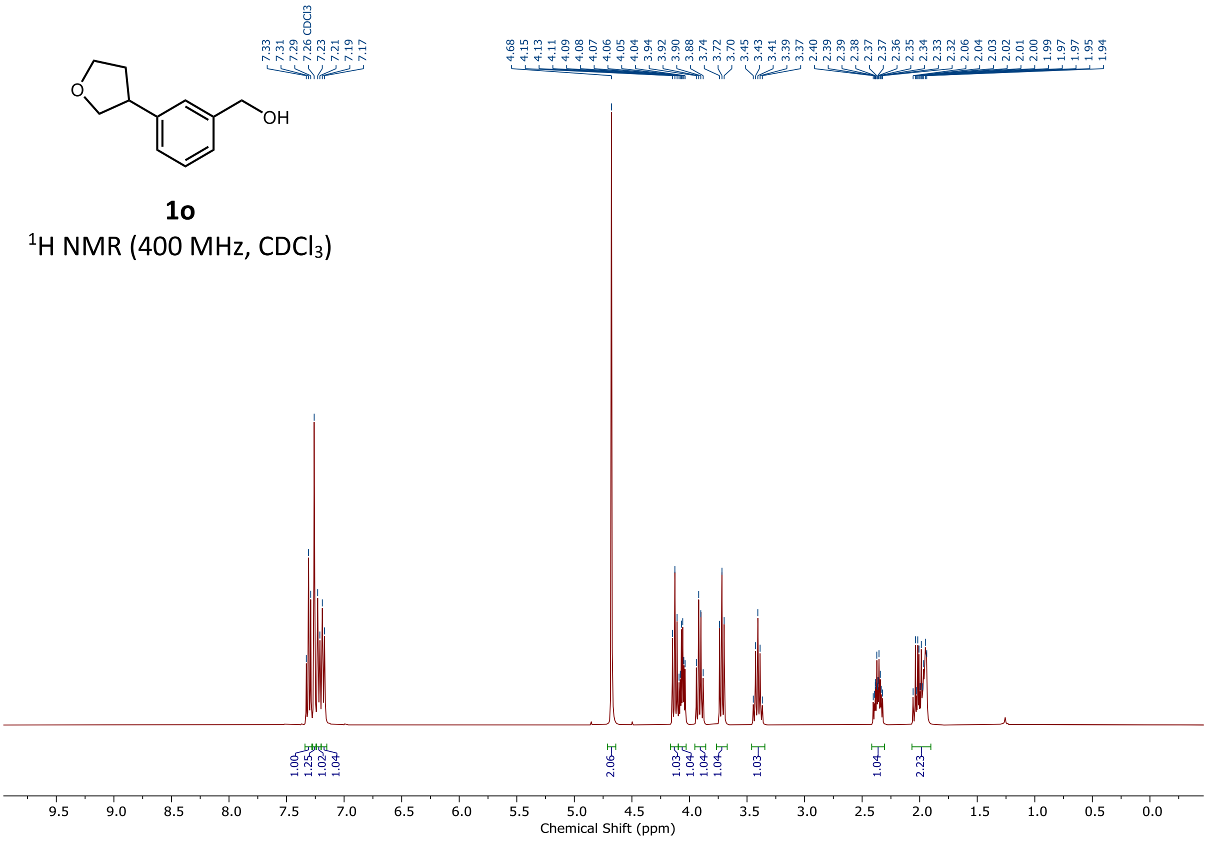


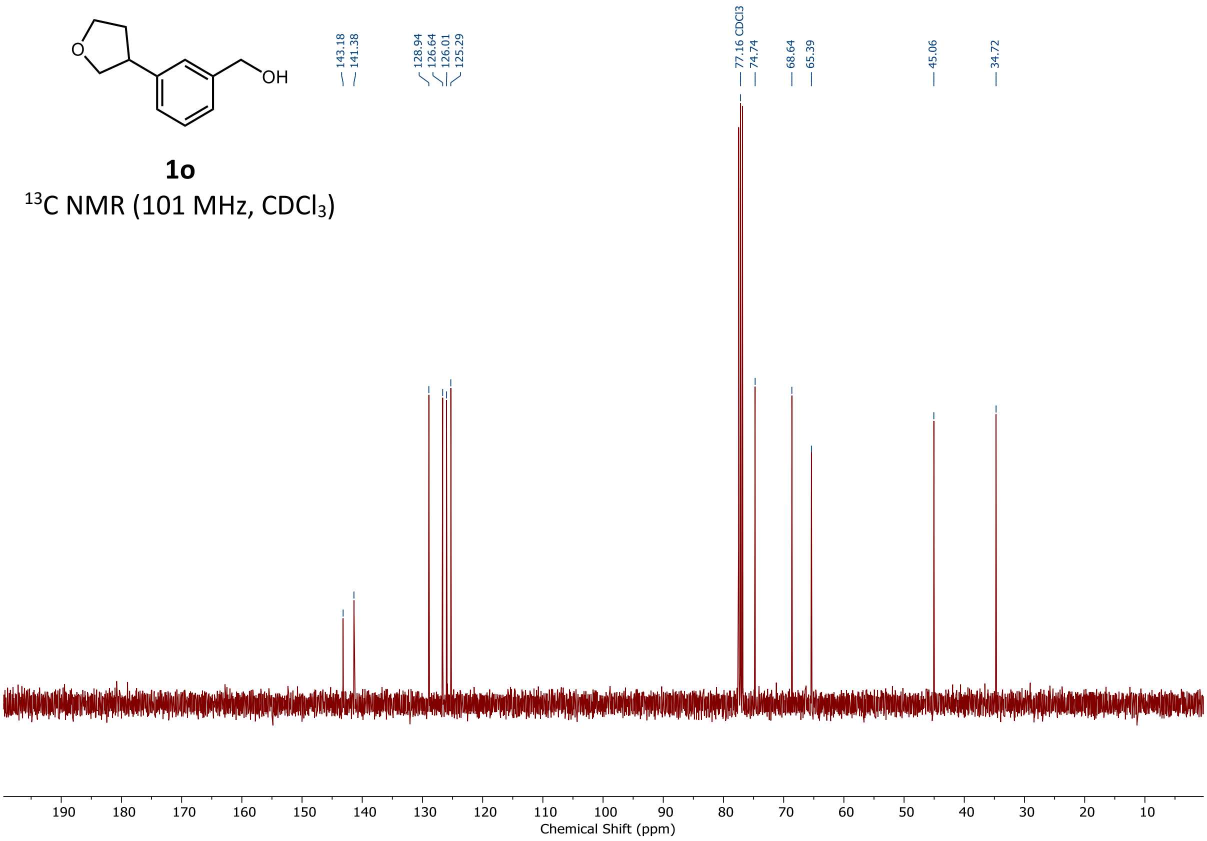

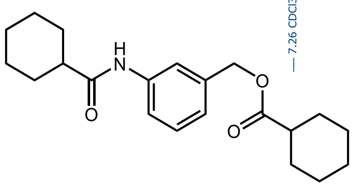

**S1**

$^{1}H$ NMR (400 MHz, $CDCl_3$)

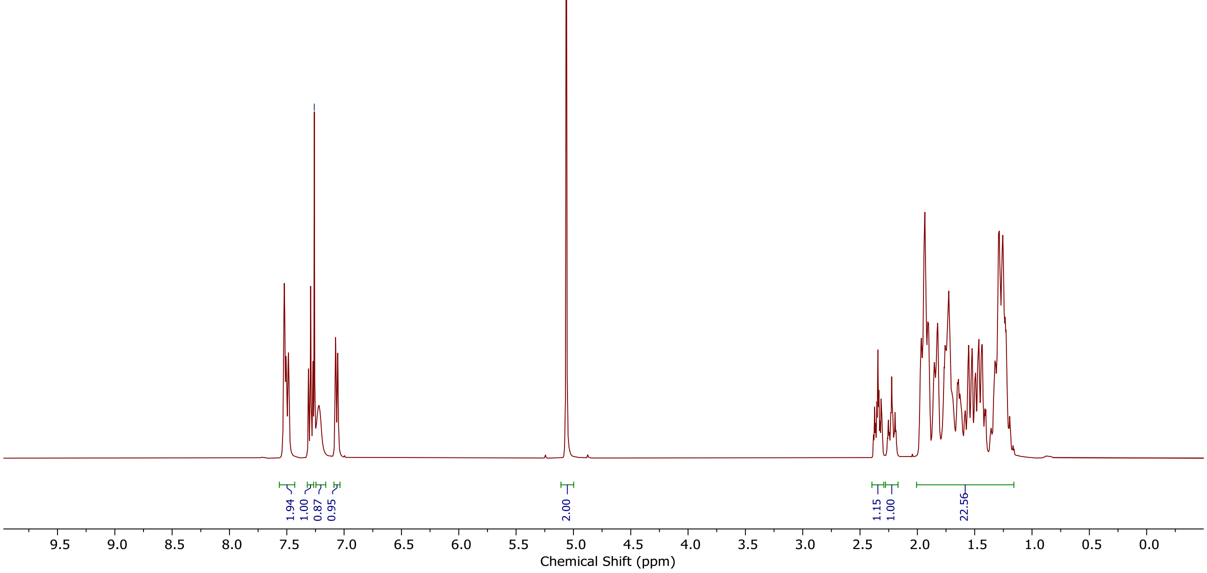


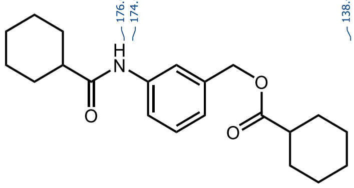

**S1**

$^{13}C$ NMR (101 MHz, $CDCl_3$)

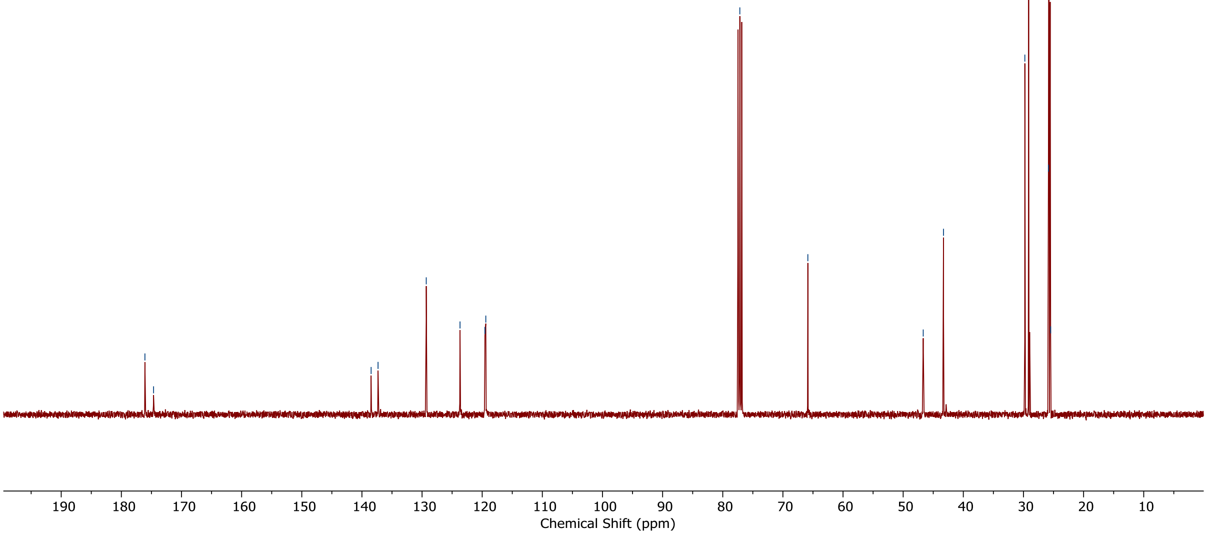

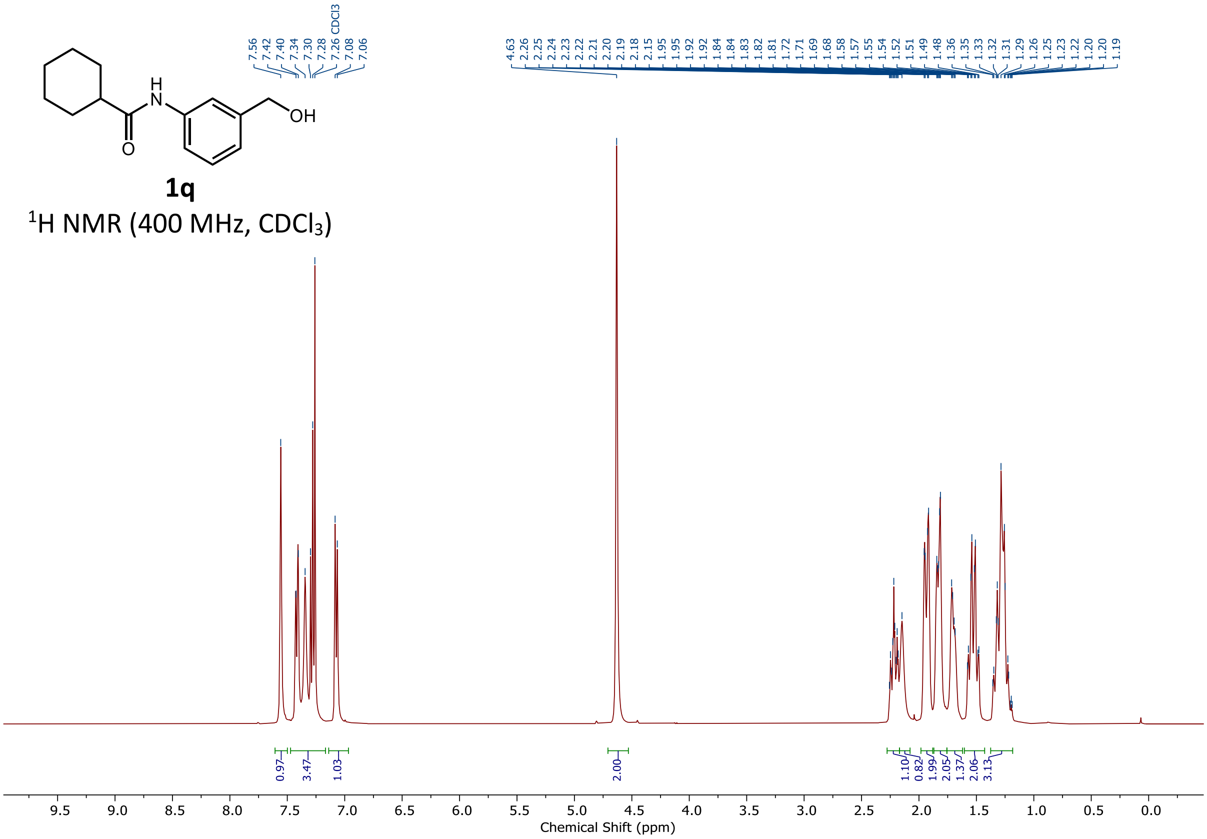


**1q**

$^{1}$H NMR (400 MHz, $CDCl_3$)

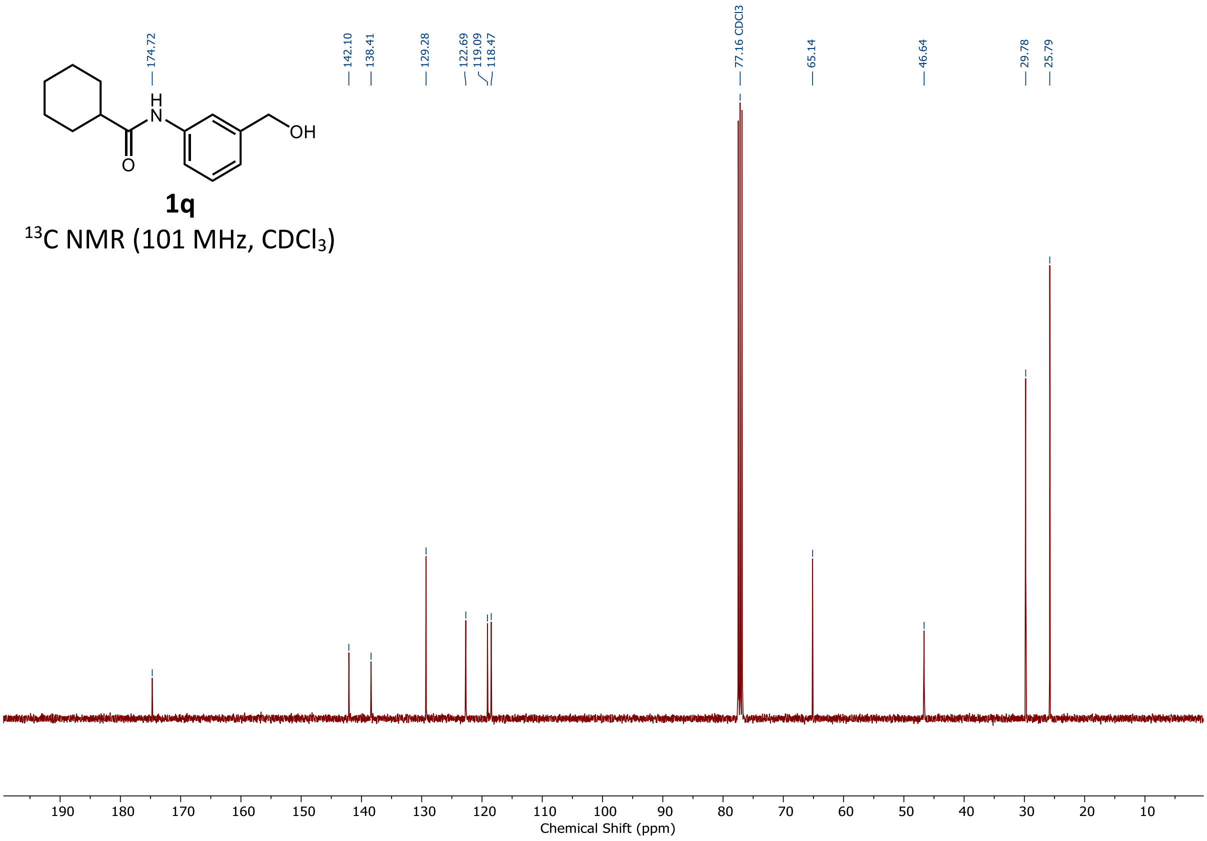


**1q**

$^{13}$C NMR (101 MHz, $CDCl_3$)

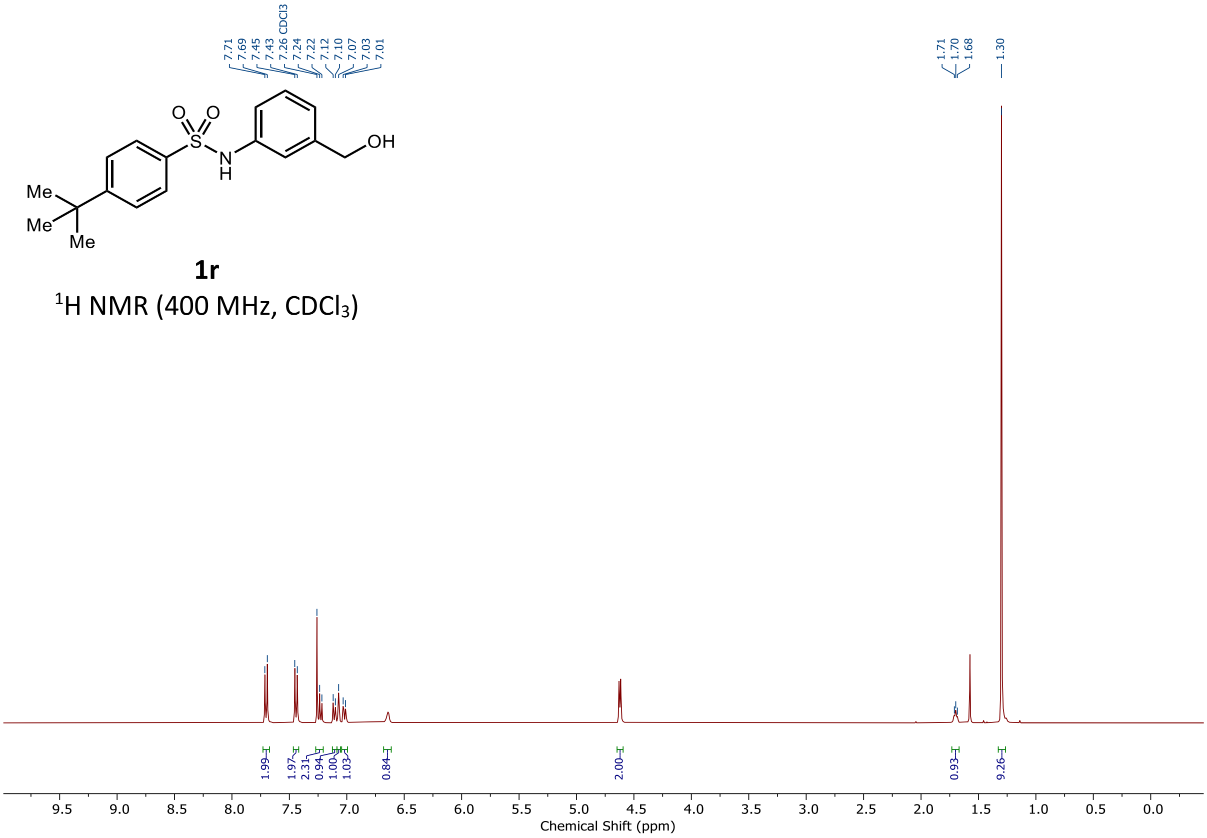


**1r**

$^{1}$H NMR (400 MHz, $CDCl_3$)

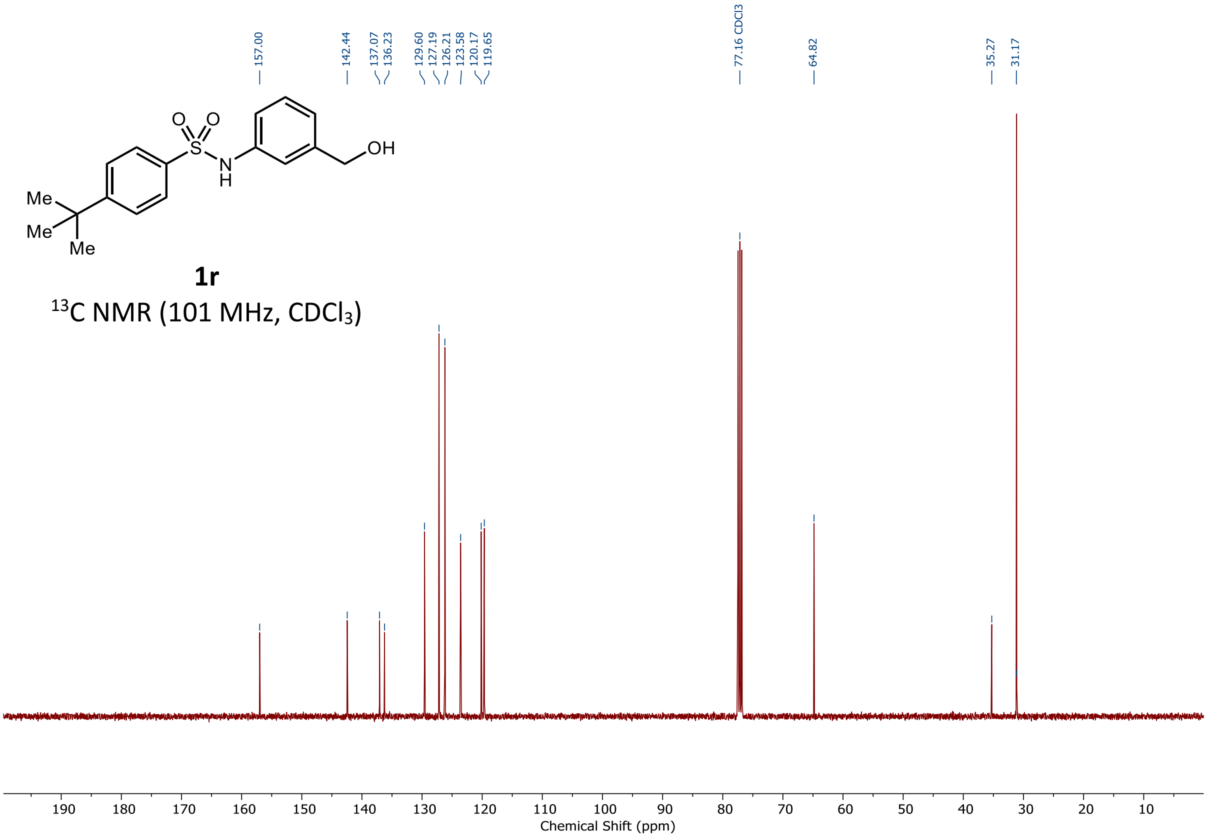


**1r**

$^{13}$C NMR (101 MHz, $CDCl_3$)

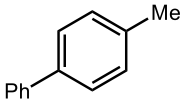


**2a**

$^{1}$H NMR (400 MHz, $CDCl_3$)

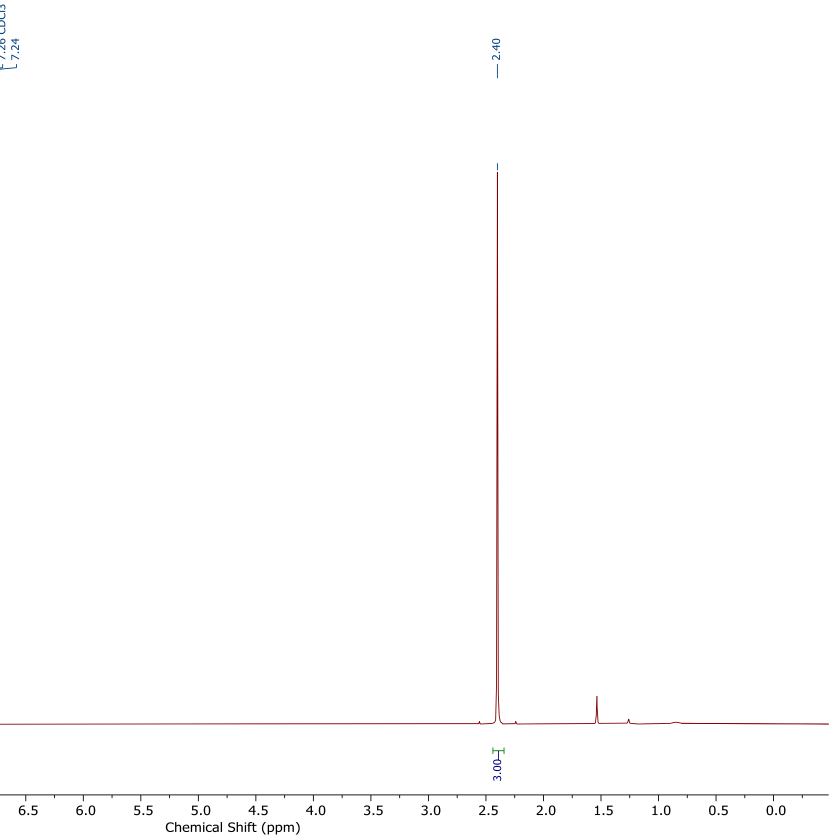


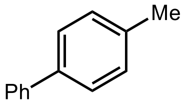


**2a**

$^{13}$C NMR (101 MHz, $CDCl_3$)

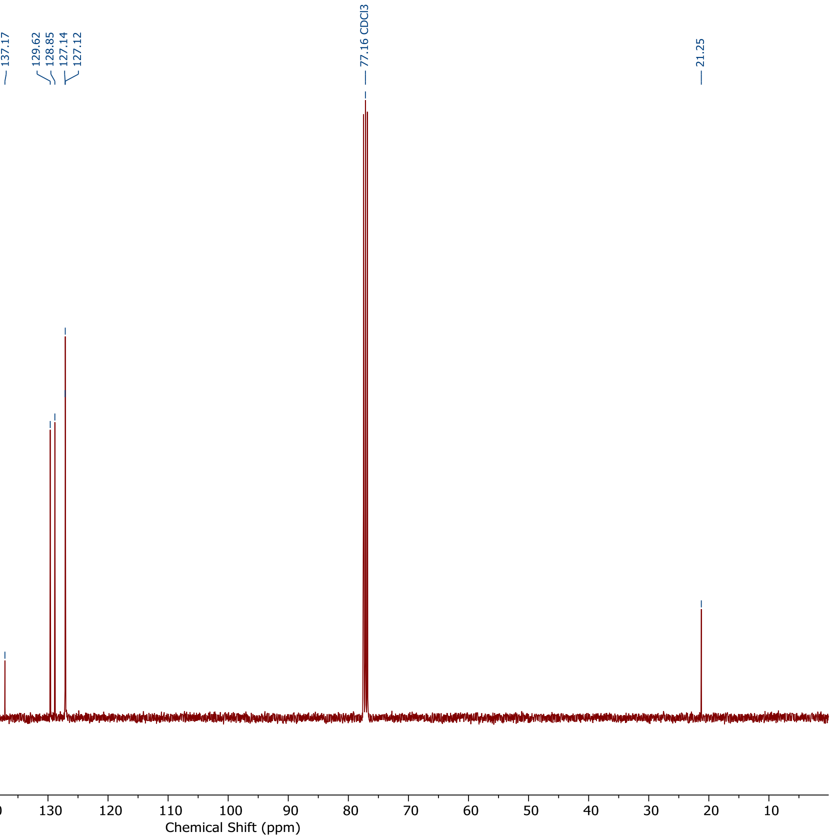

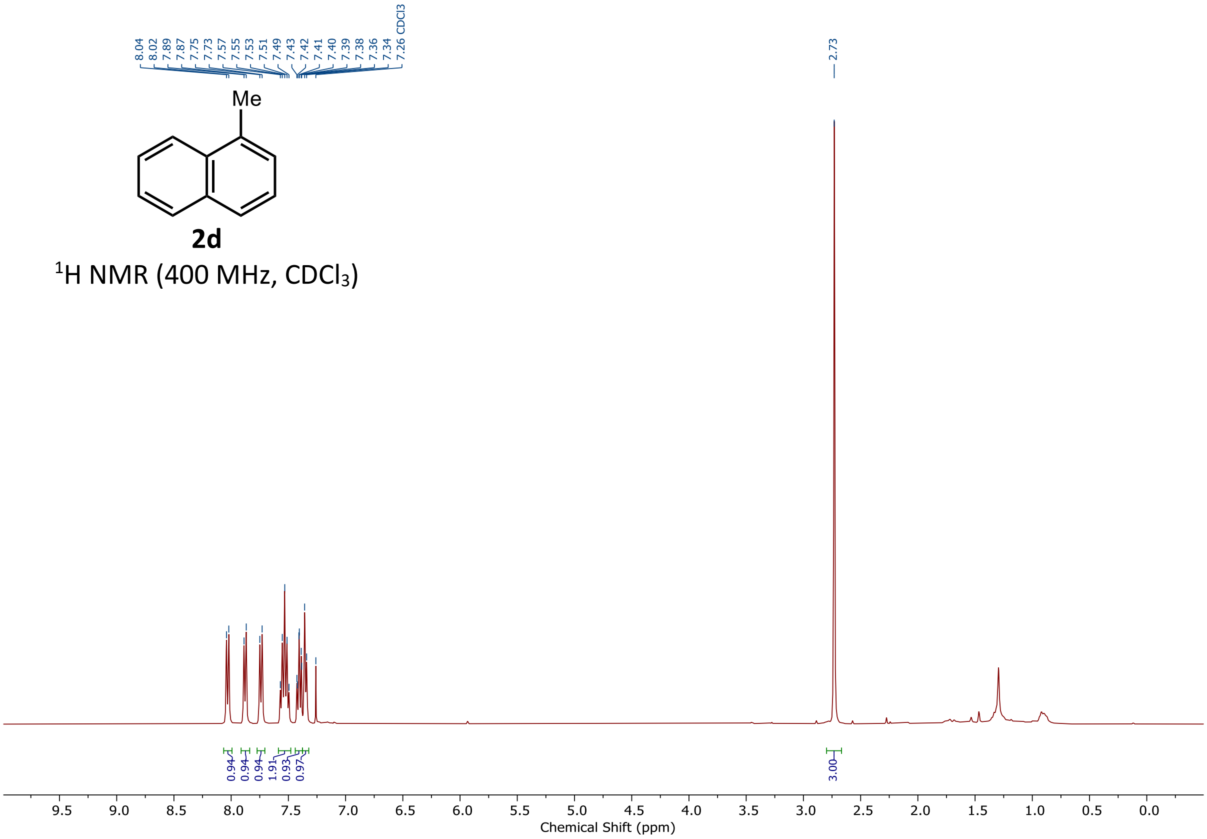
8.04
8.02
7.89
7.87
7.75
7.73
7.57
7.55
7.53
7.51
7.49
7.43
7.42
7.41
7.40
7.39
7.38
7.36
7.34
7.26 CDCl3
2.73
Me
2d
$^{1}$H NMR (400 MHz, $CDCl_3$)
0.94
0.94
0.94
1.91
0.93
0.97
3.00
9.5 9.0 8.5 8.0 7.5 7.0 6.5 6.0 5.5 5.0 4.5 4.0 3.5 3.0 2.5 2.0 1.5 1.0 0.5 0.0
Chemical Shift (ppm)

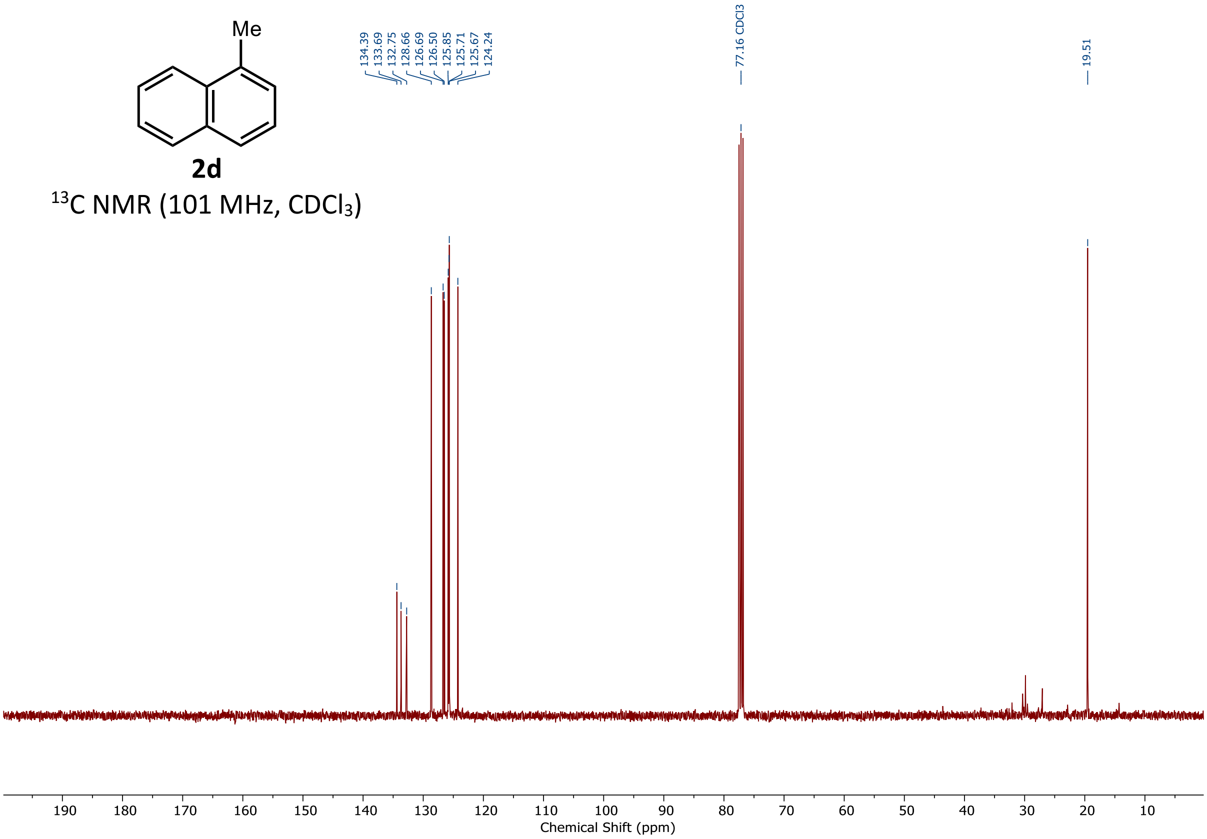
Me
2d
$^{13}$C NMR (101 MHz, $CDCl_3$)
134.39
133.69
132.75
128.66
126.69
126.50
125.85
125.71
125.67
124.24
77.16 CDCl3
19.51
190 180 170 160 150 140 130 120 110 100 90 80 70 60 50 40 30 20 10
Chemical Shift (ppm)

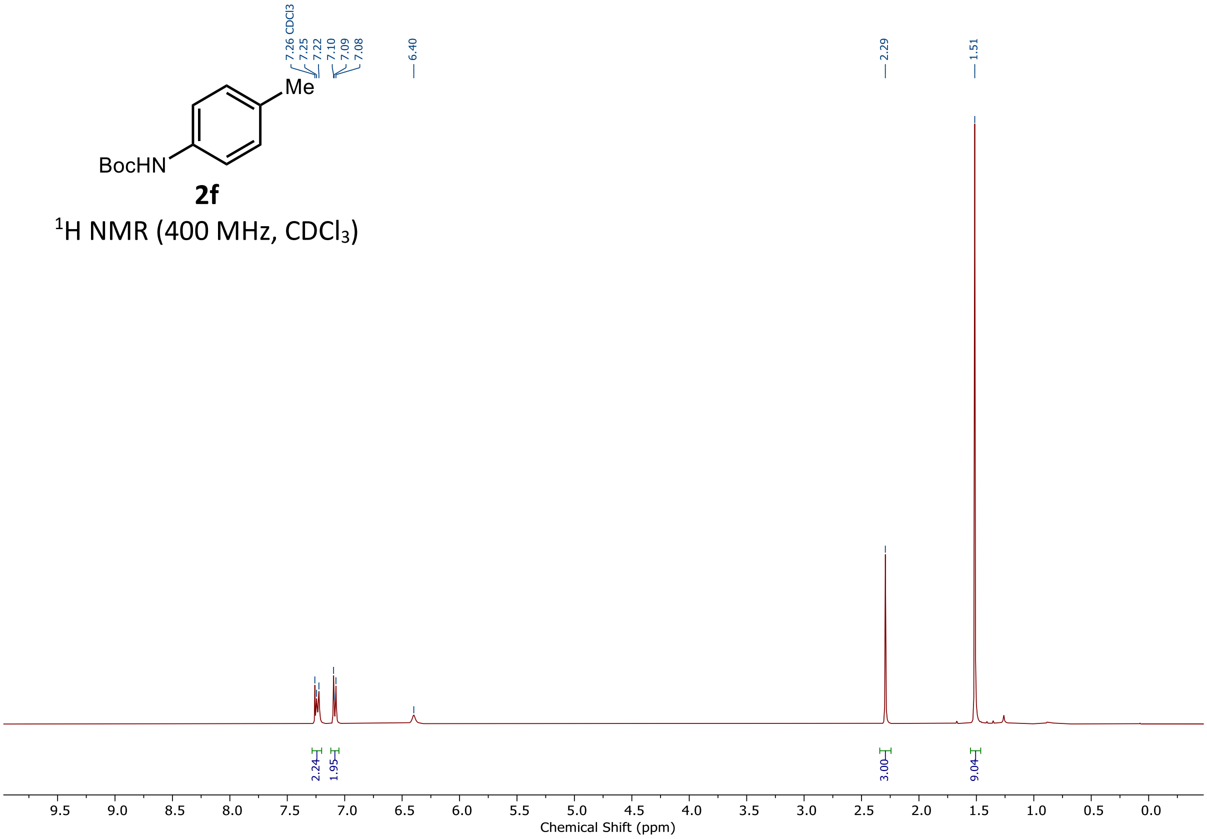

7.26 CDCl3
7.25
7.22
7.10
7.09
7.08
6.40
2.29
1.51
Me
BocHN
2f
¹H NMR (400 MHz, CDCl₃)
2.24
1.95
3.00
9.04
Chemical Shift (ppm)


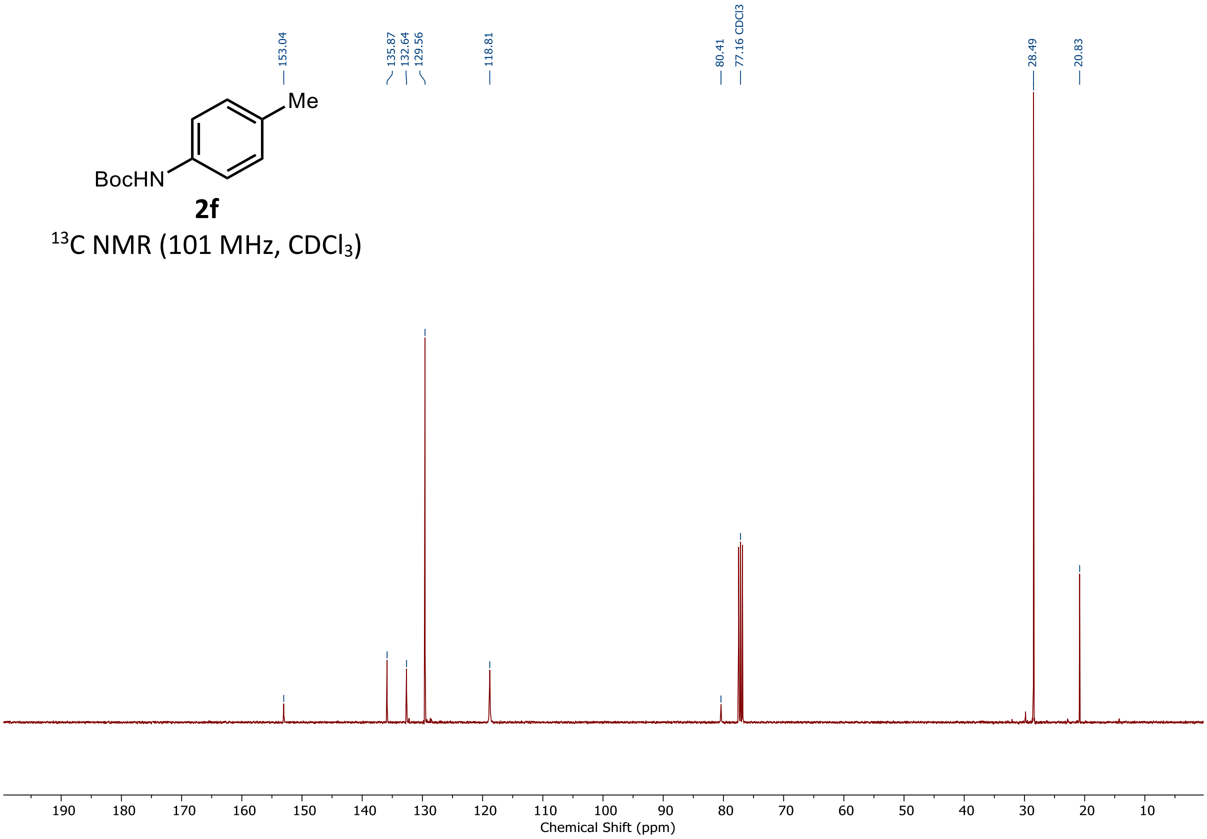

153.04
135.87
132.64
129.56
118.81
80.41
77.16 CDCl3
28.49
20.83
Me
BocHN
2f
¹³C NMR (101 MHz, CDCl₃)
Chemical Shift (ppm)

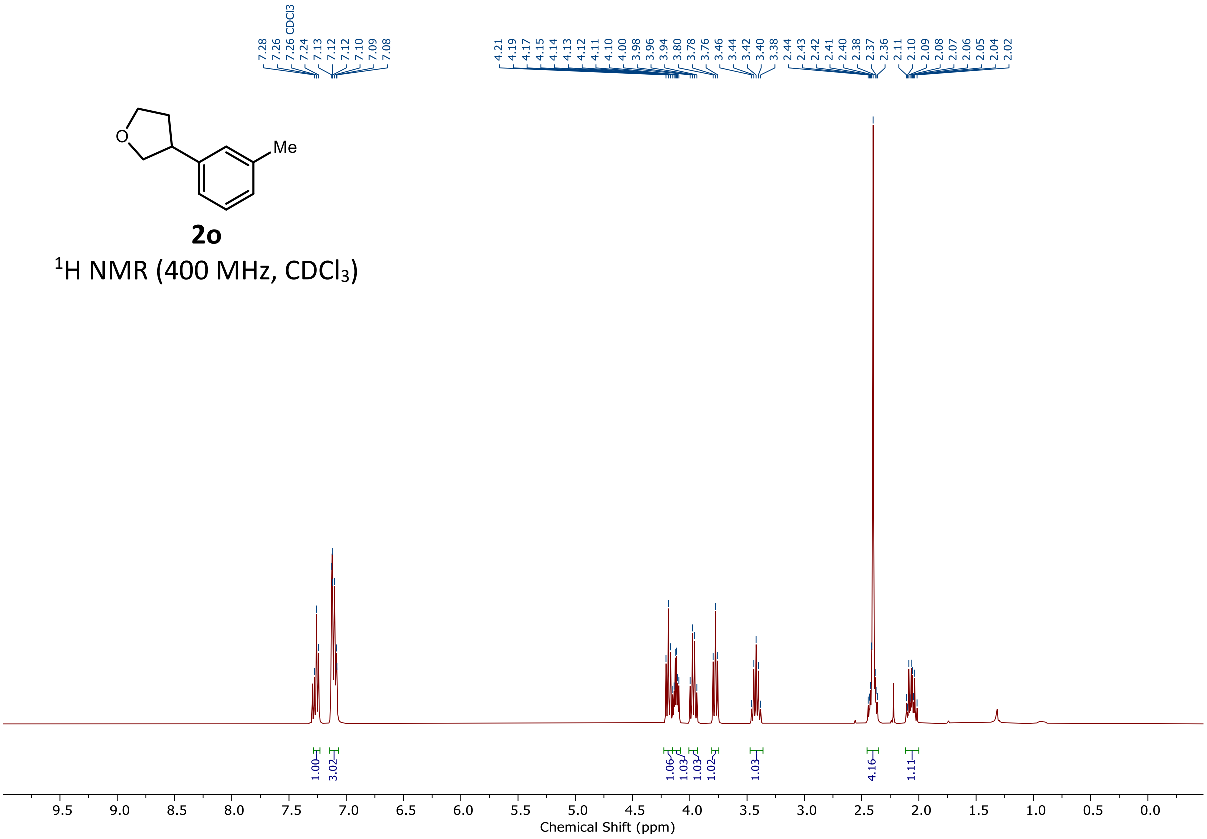


**2o**

$^{1}H$ NMR (400 MHz, $CDCl_3$)

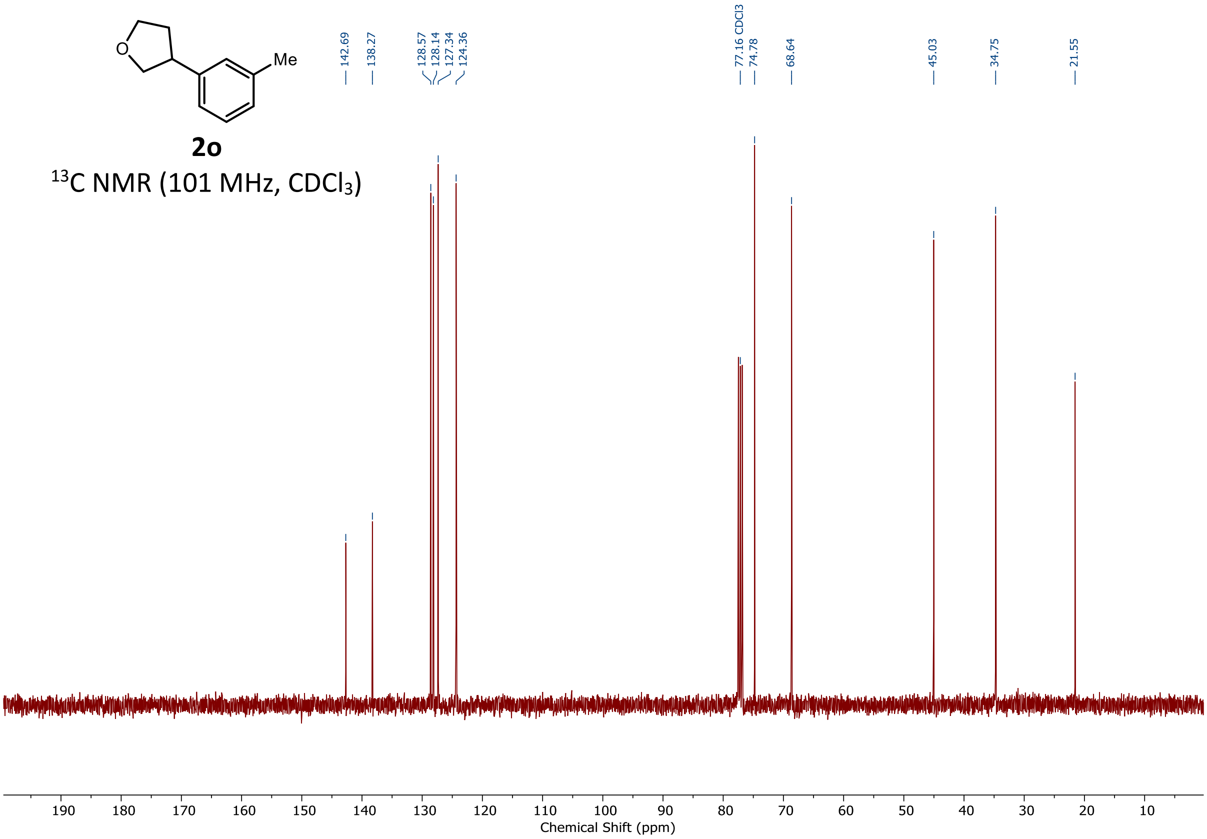


**2o**

$^{13}C$ NMR (101 MHz, $CDCl_3$)

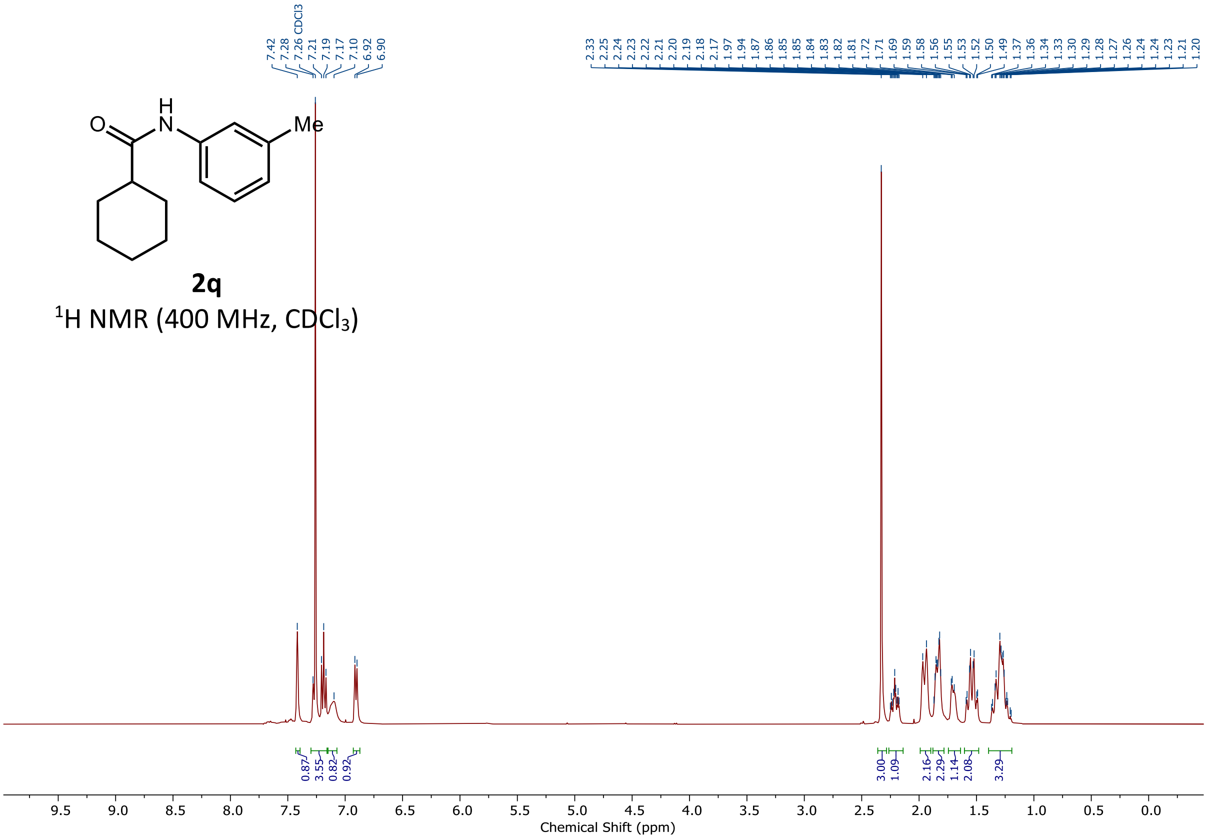


**2q**

$^1$H NMR (400 MHz, $CDCl_3$)

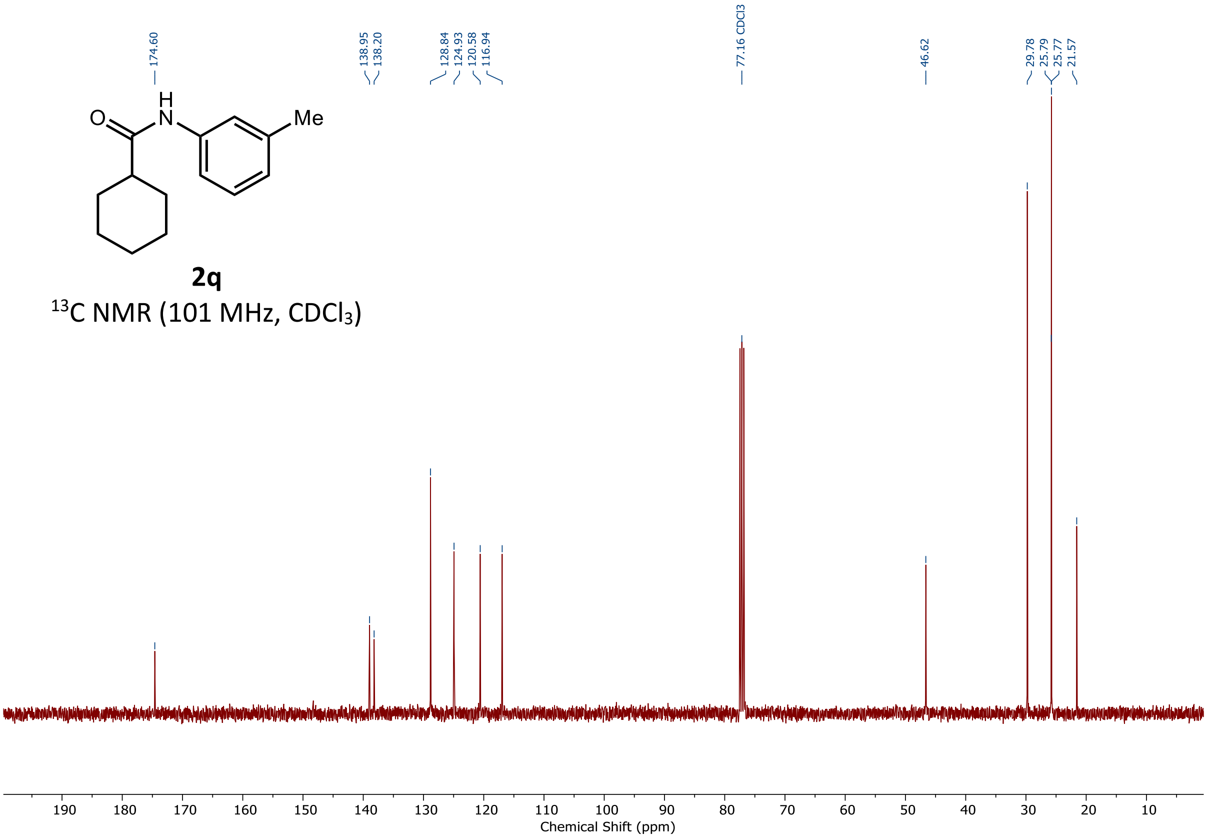


**2q**

$^{13}$C NMR (101 MHz, $CDCl_3$)

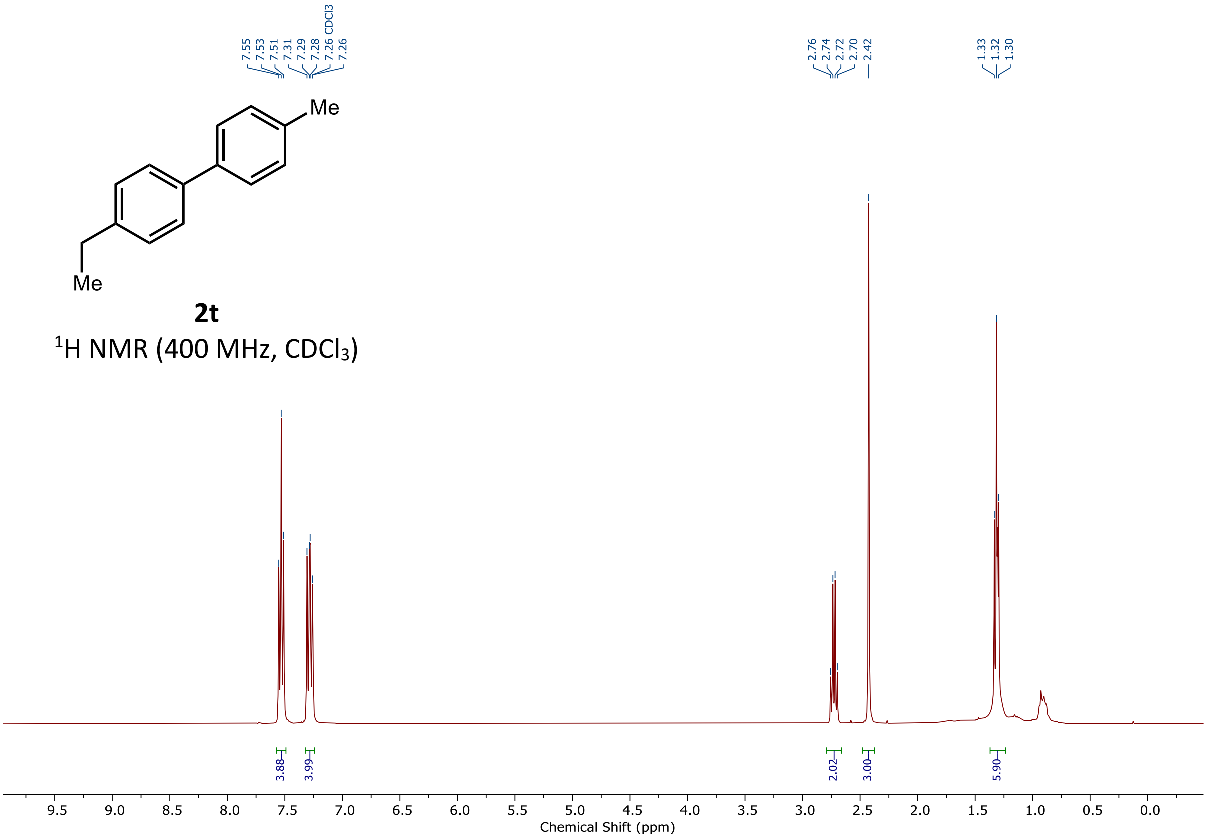


**2t**

$^{1}$H NMR (400 MHz, $CDCl_3$)

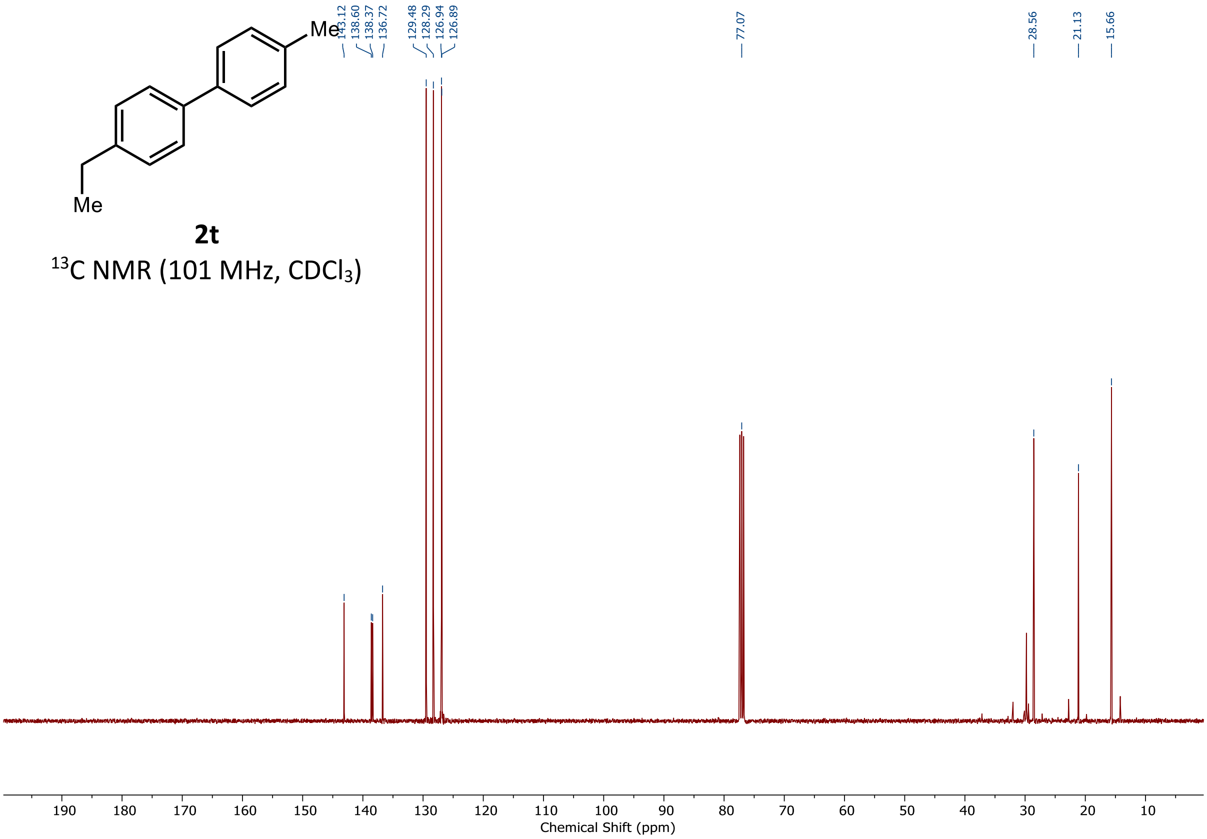


**2t**

$^{13}$C NMR (101 MHz, $CDCl_3$)

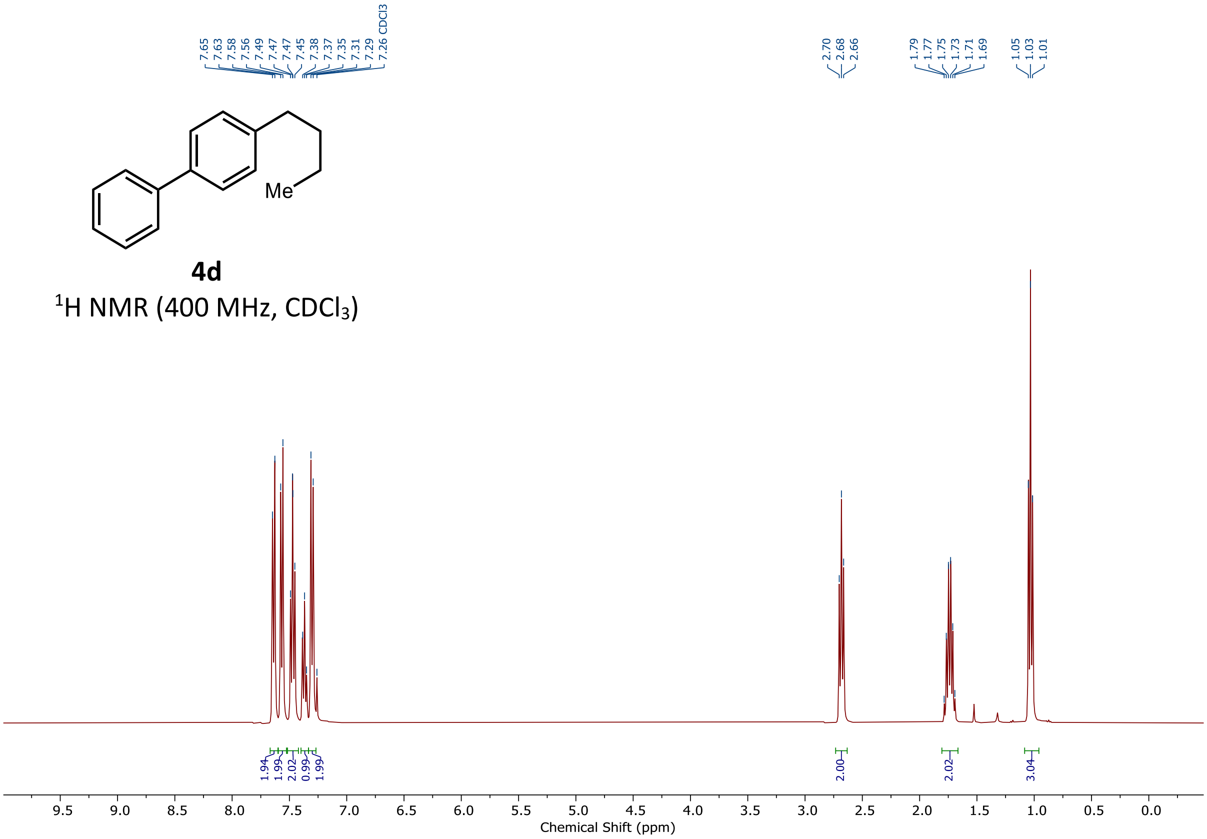

Me
4d
$^{1}$H NMR (400 MHz, CDCl$_3$)
7.65 7.63 7.58 7.56 7.49 7.47 7.47 7.45 7.38 7.37 7.35 7.31 7.29 7.26 CDCl3
2.70 2.68 2.66
1.79 1.77 1.75 1.73 1.71 1.69
1.05 1.03 1.01
1.94 1.99 2.02 0.99 1.99
2.00
2.02
3.04
Chemical Shift (ppm)


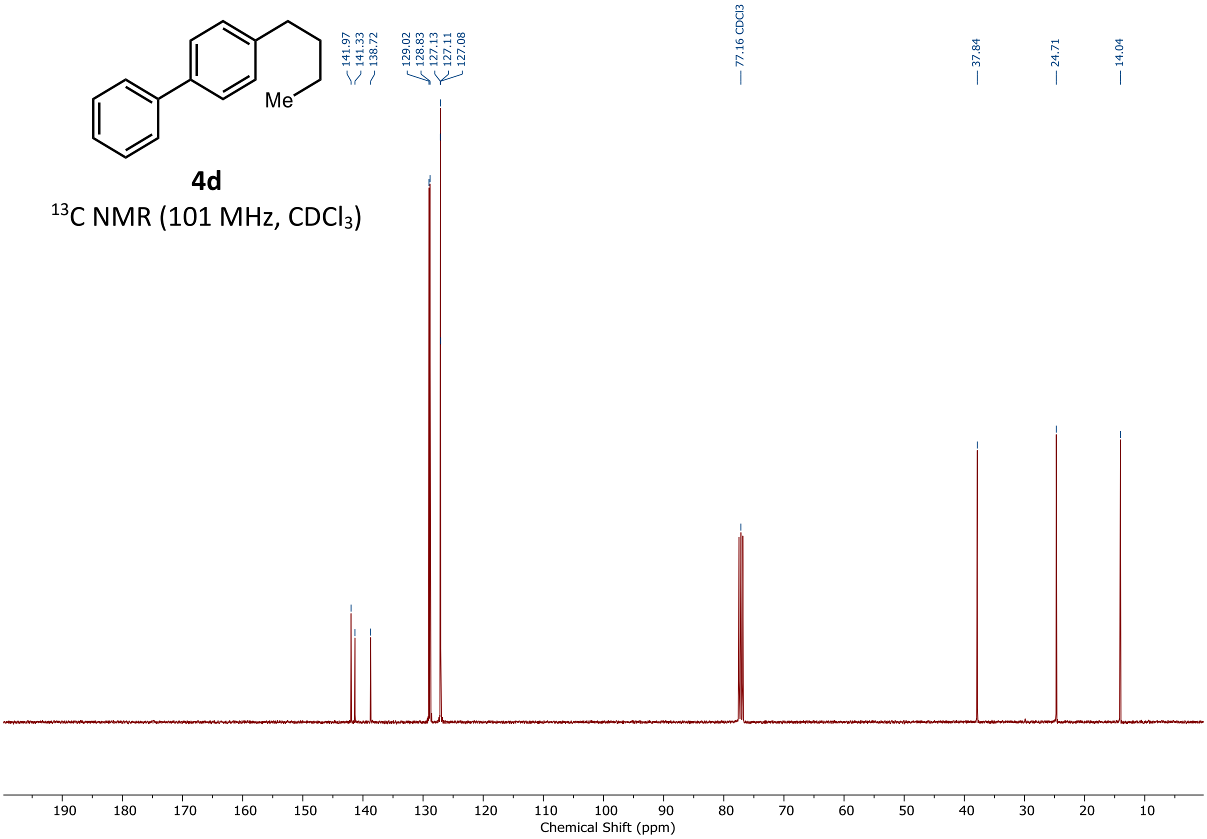

Me
4d
$^{13}$C NMR (101 MHz, CDCl$_3$)
141.97 141.33 138.72
129.02 128.83 127.13 127.11 127.08
77.16 CDCl3
37.84
24.71
14.04
Chemical Shift (ppm)

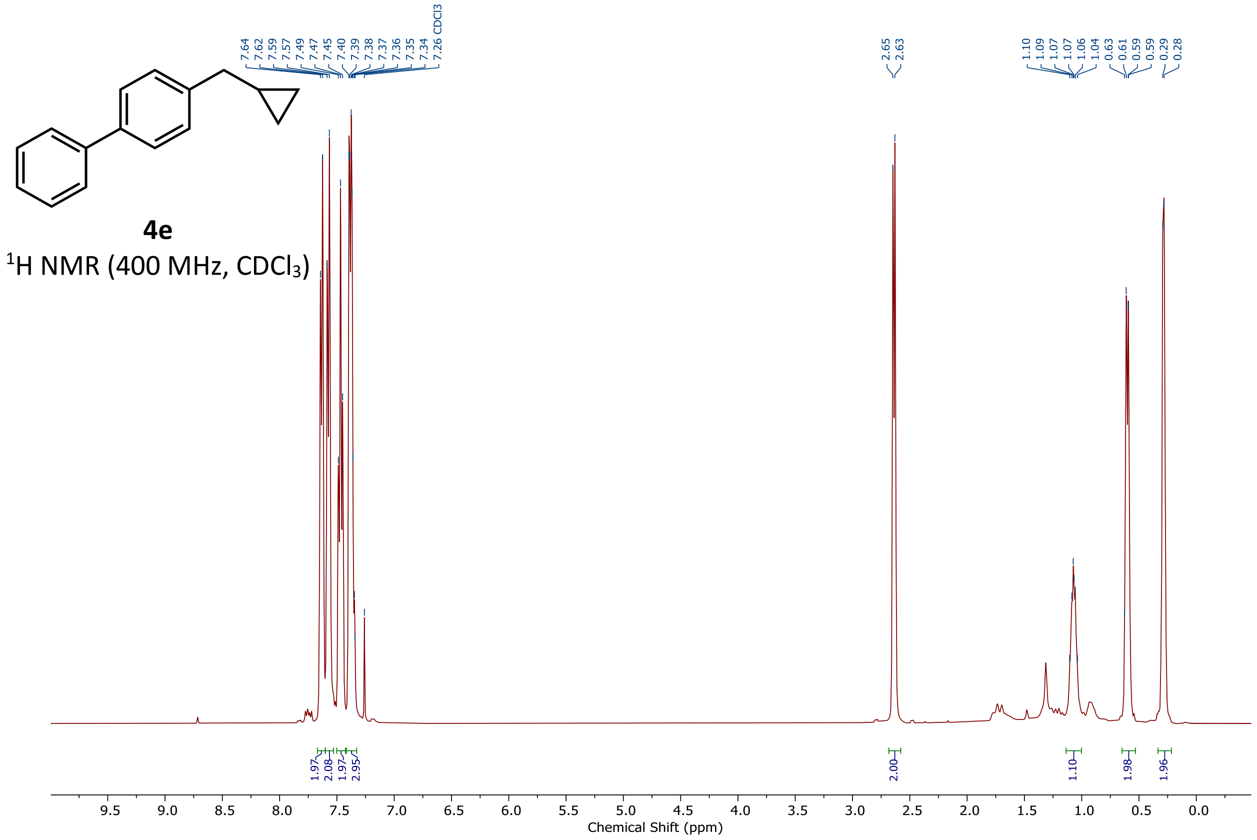

7.64
7.62
7.59
7.57
7.49
7.47
7.45
7.40
7.39
7.38
7.37
7.36
7.35
7.34
7.26 CDCl3
2.65
2.63
1.10
1.09
1.07
1.07
1.06
1.04
0.63
0.61
0.59
0.59
0.29
0.28
4e
$^{1}$H NMR (400 MHz, CDCl$_3$)
1.97
2.08
1.97
2.95
2.00
1.10
1.98
1.96
Chemical Shift (ppm)


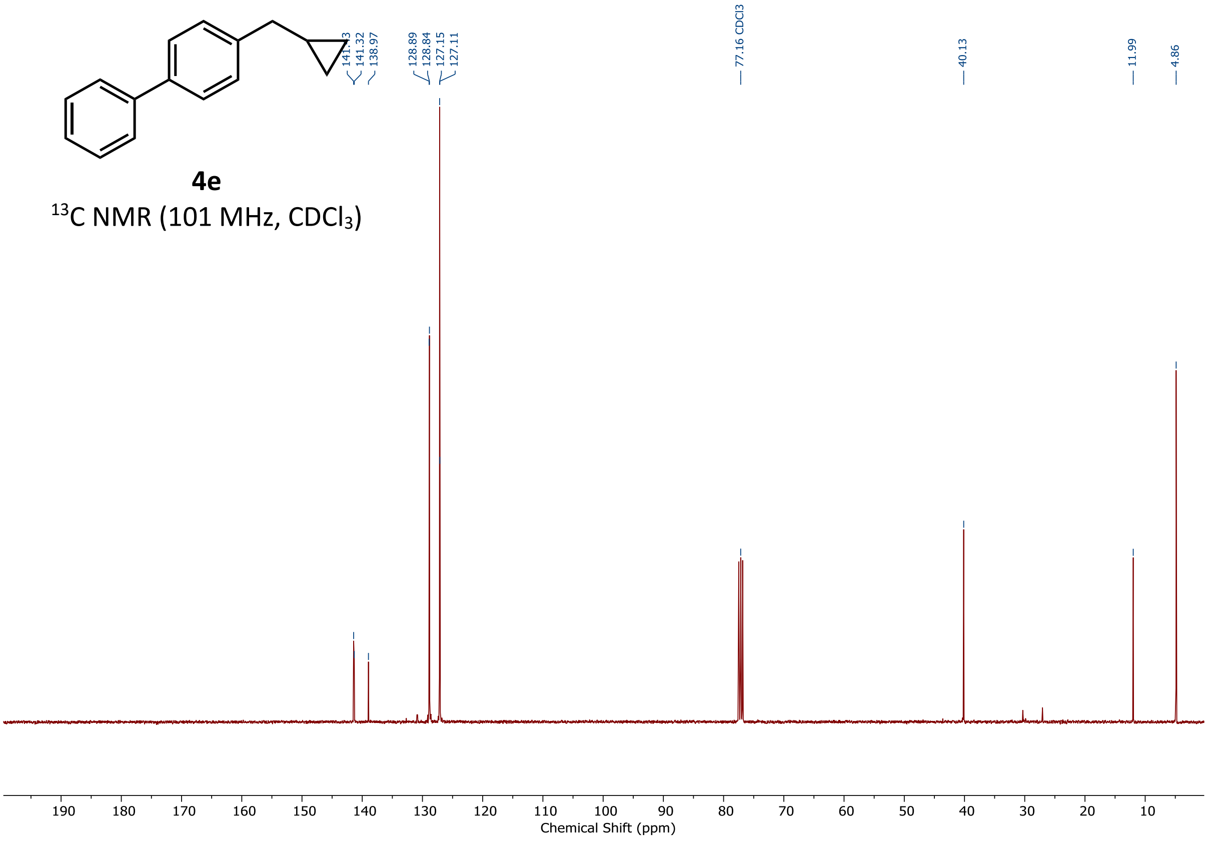

141.32
138.97
128.89
128.84
127.15
127.11
77.16 CDCl3
40.13
11.99
4.86
4e
$^{13}$C NMR (101 MHz, CDCl$_3$)
Chemical Shift (ppm)

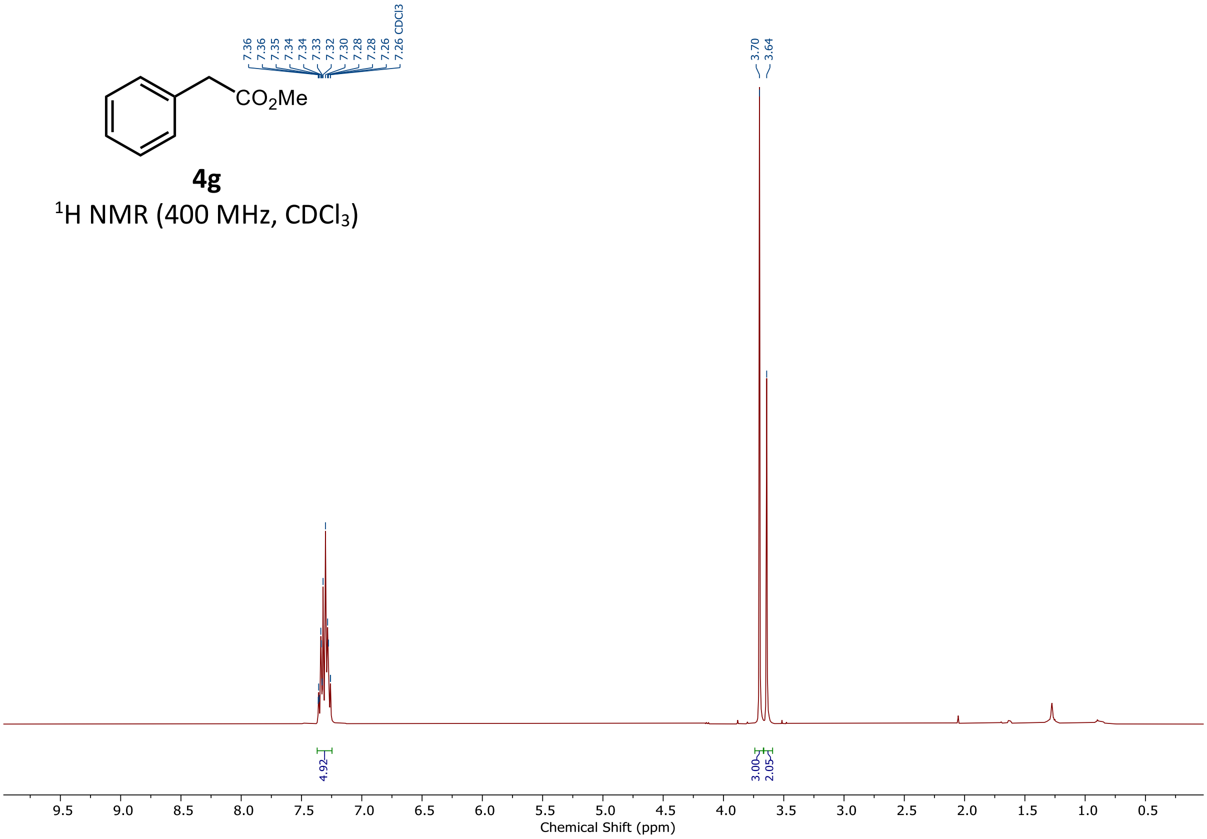
7.36
7.36
7.35
7.34
7.34
7.33
7.32
7.30
7.28
7.28
7.26
7.26 CDCl3
3.70
3.64
CO2Me
4g
1H NMR (400 MHz, CDCl3)
4.92
3.00
2.05
9.5 9.0 8.5 8.0 7.5 7.0 6.5 6.0 5.5 5.0 4.5 4.0 3.5 3.0 2.5 2.0 1.5 1.0 0.5
Chemical Shift (ppm)

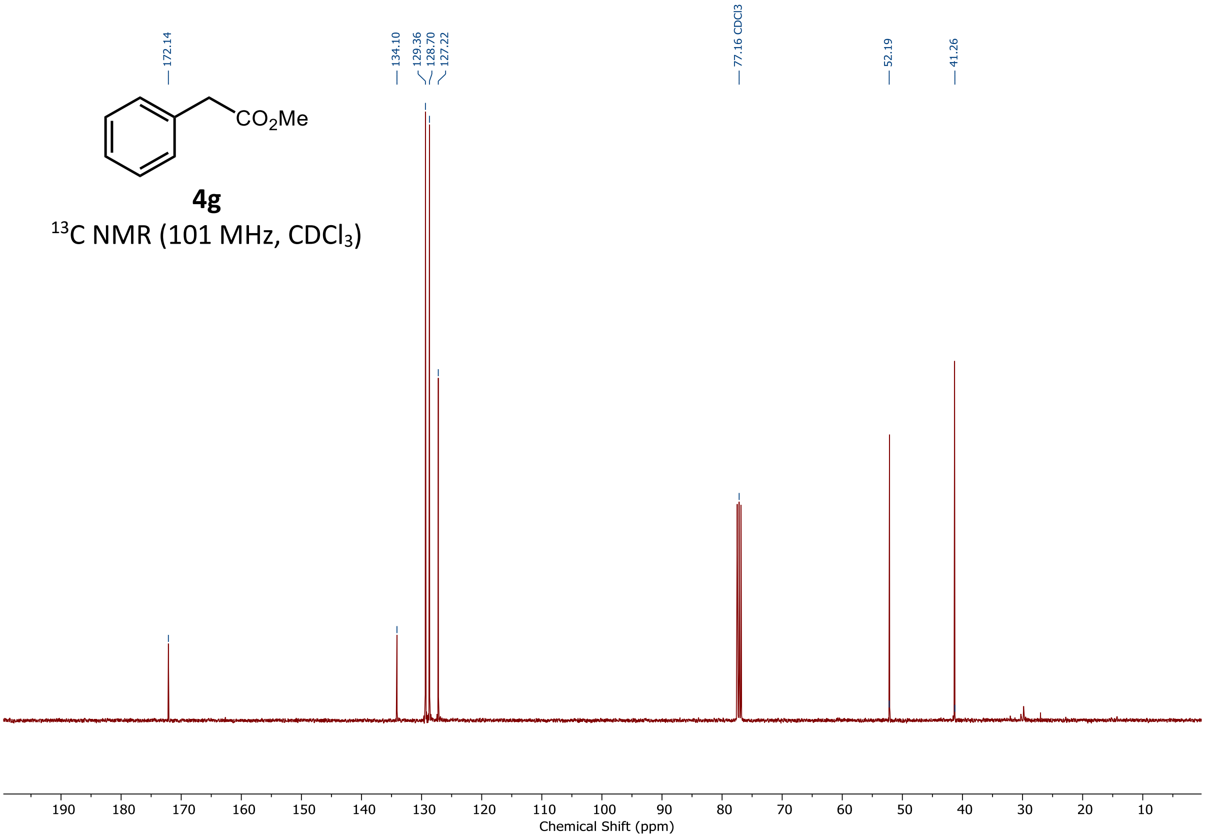
172.14
134.10
129.36
128.70
127.22
77.16 CDCl3
52.19
41.26
CO2Me
4g
13C NMR (101 MHz, CDCl3)
190 180 170 160 150 140 130 120 110 100 90 80 70 60 50 40 30 20 10
Chemical Shift (ppm)

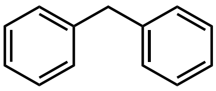

**4h**

$^{1}H$ NMR (400 MHz, $CDCl_3$)

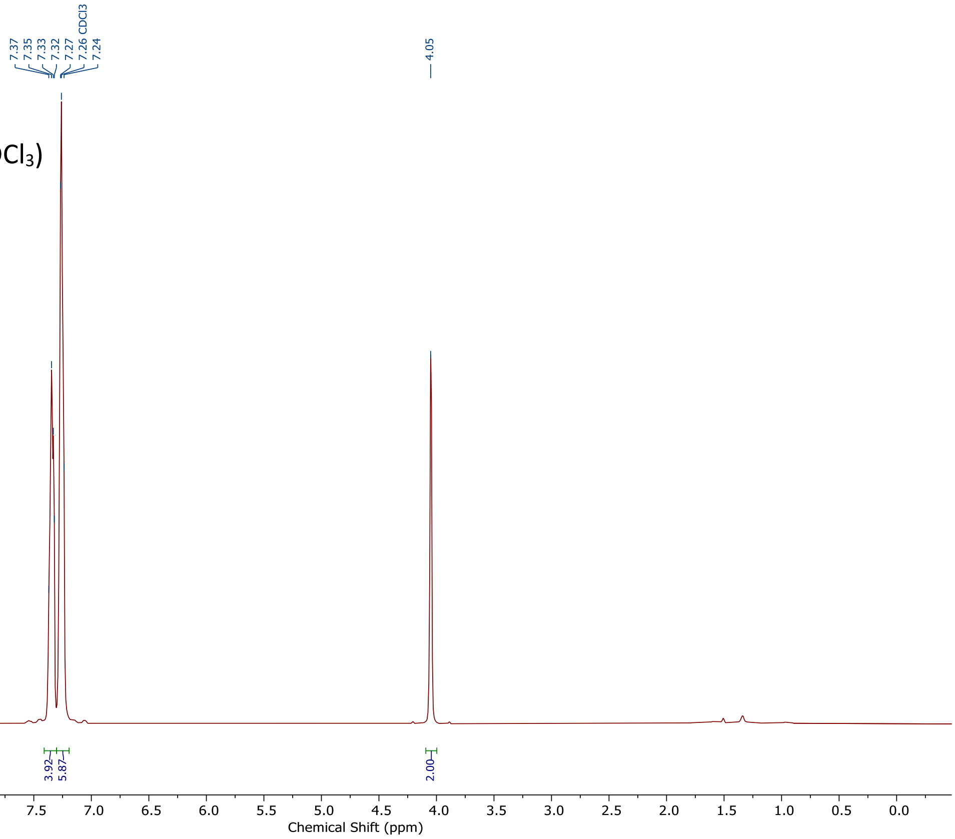


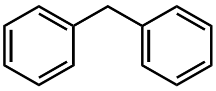

**4h**

$^{13}C$ NMR (101 MHz, $CDCl_3$)

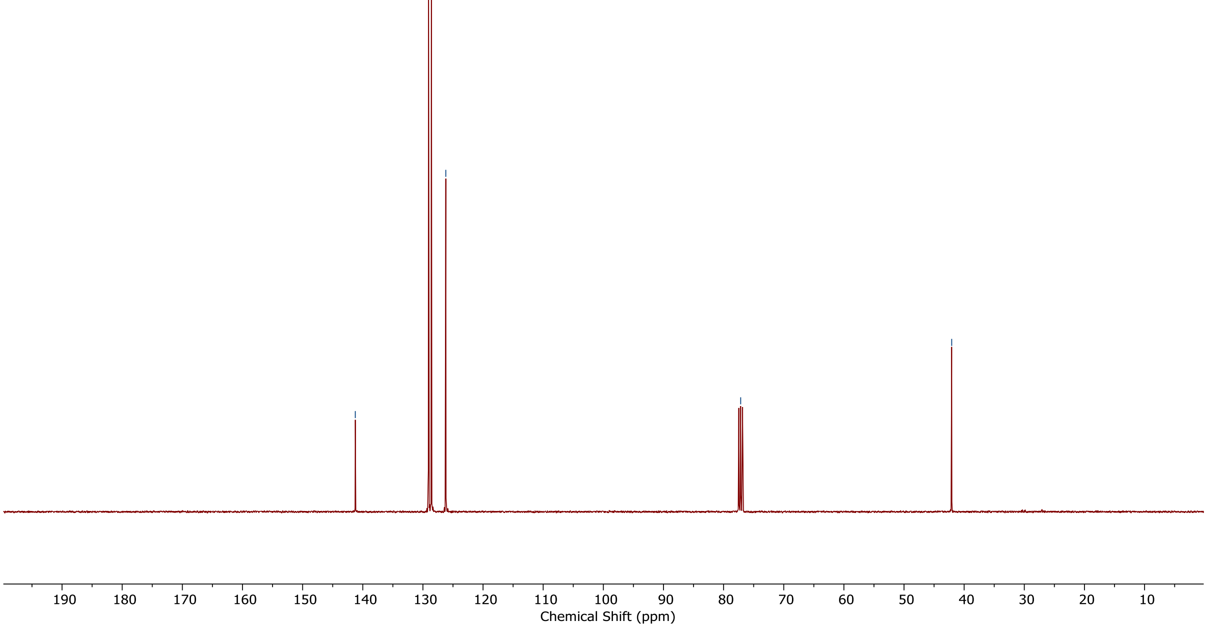

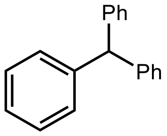

**4i**

$^{1}$H NMR (400 MHz, $CDCl_3$)

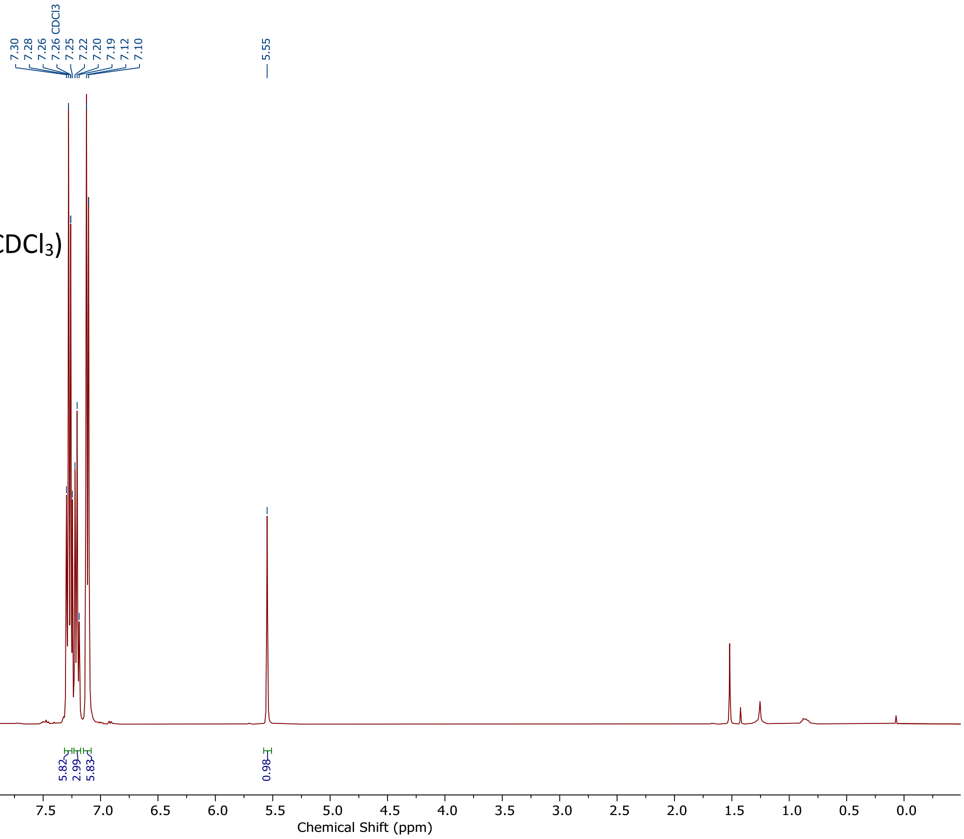


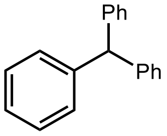

**4i**

$^{13}$C NMR (101 MHz, $CDCl_3$)

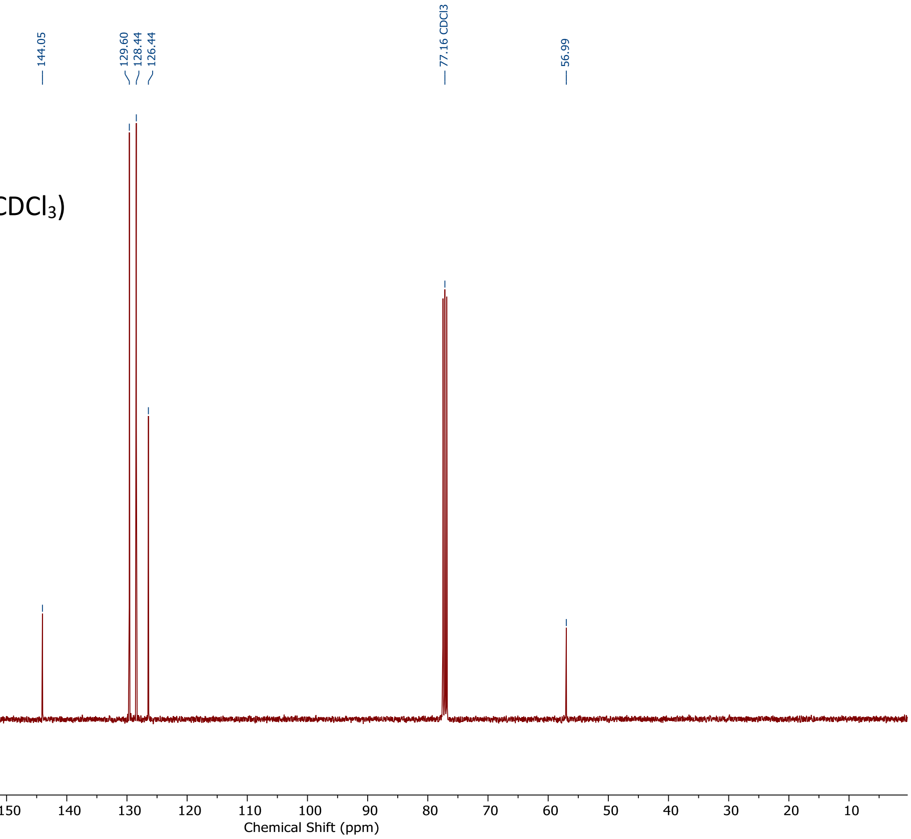

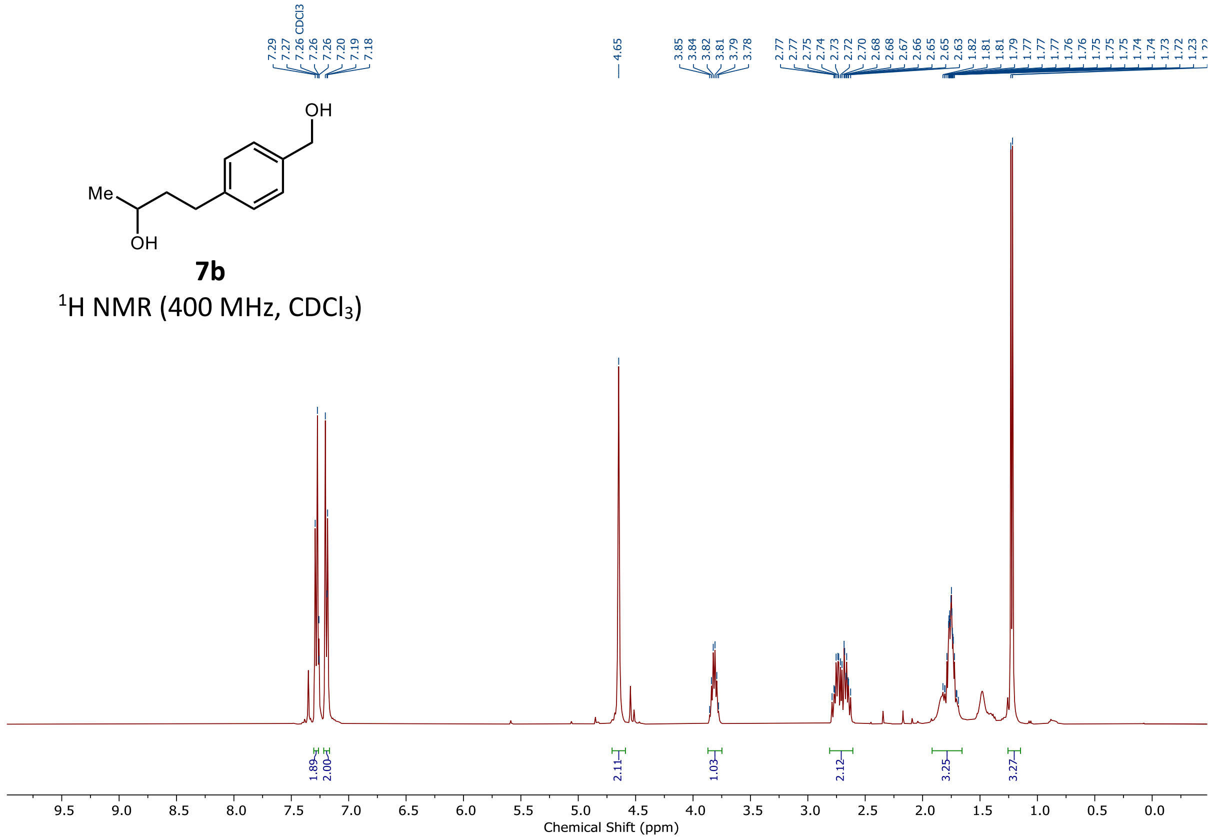
7.29
7.27
7.26 CDCl3
7.26
7.26
7.20
7.19
7.18
4.65
3.85
3.84
3.82
3.81
3.79
3.78
2.77
2.77
2.75
2.74
2.73
2.72
2.70
2.68
2.68
2.67
2.66
2.65
2.63
1.82
1.81
1.81
1.79
1.77
1.77
1.77
1.76
1.76
1.75
1.75
1.75
1.74
1.74
1.73
1.72
1.23
OH
Me
OH
7b
1H NMR (400 MHz, CDCl3)
1.89
2.00
2.11
1.03
2.12
3.25
3.27
9.5 9.0 8.5 8.0 7.5 7.0 6.5 6.0 5.5 5.0 4.5 4.0 3.5 3.0 2.5 2.0 1.5 1.0 0.5 0.0
Chemical Shift (ppm)

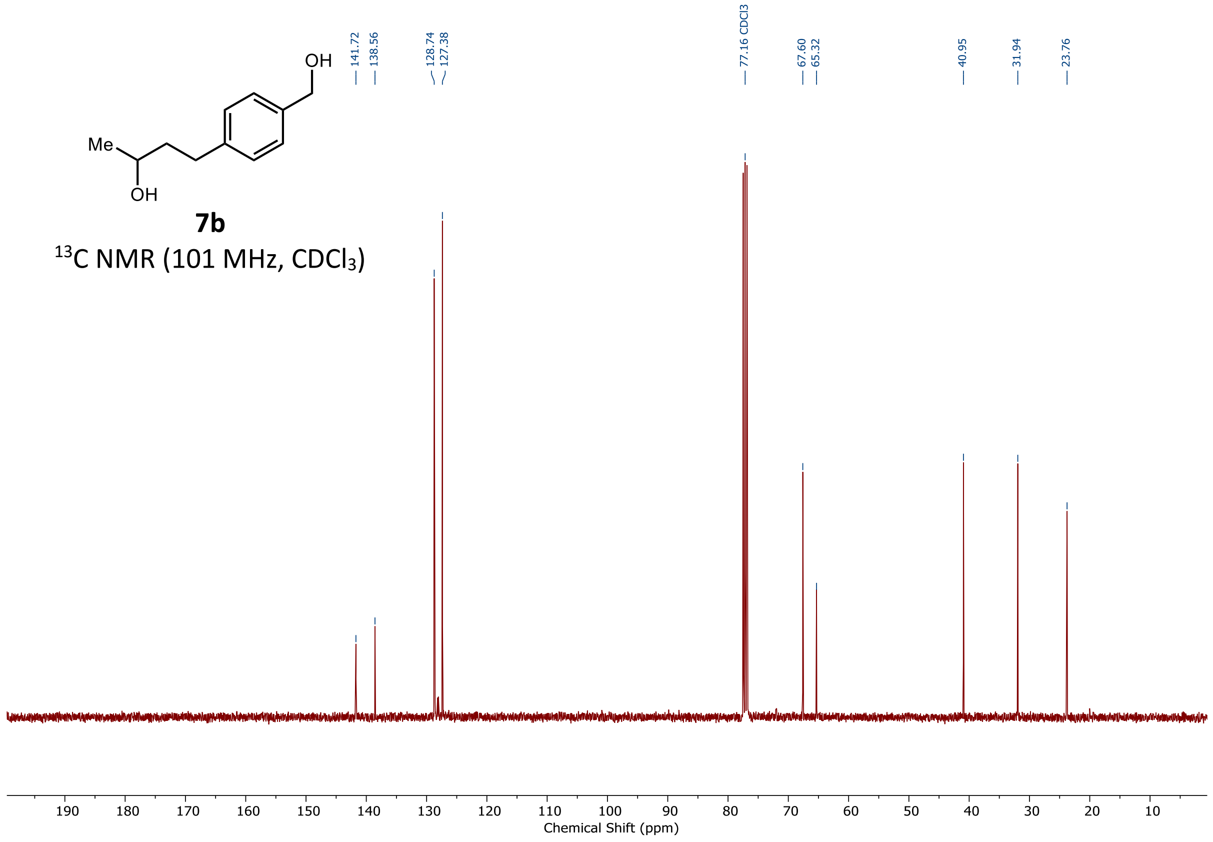
141.72
138.56
128.74
127.38
77.16 CDCl3
67.60
65.32
40.95
31.94
23.76
OH
Me
OH
7b
13C NMR (101 MHz, CDCl3)
190 180 170 160 150 140 130 120 110 100 90 80 70 60 50 40 30 20 10
Chemical Shift (ppm)

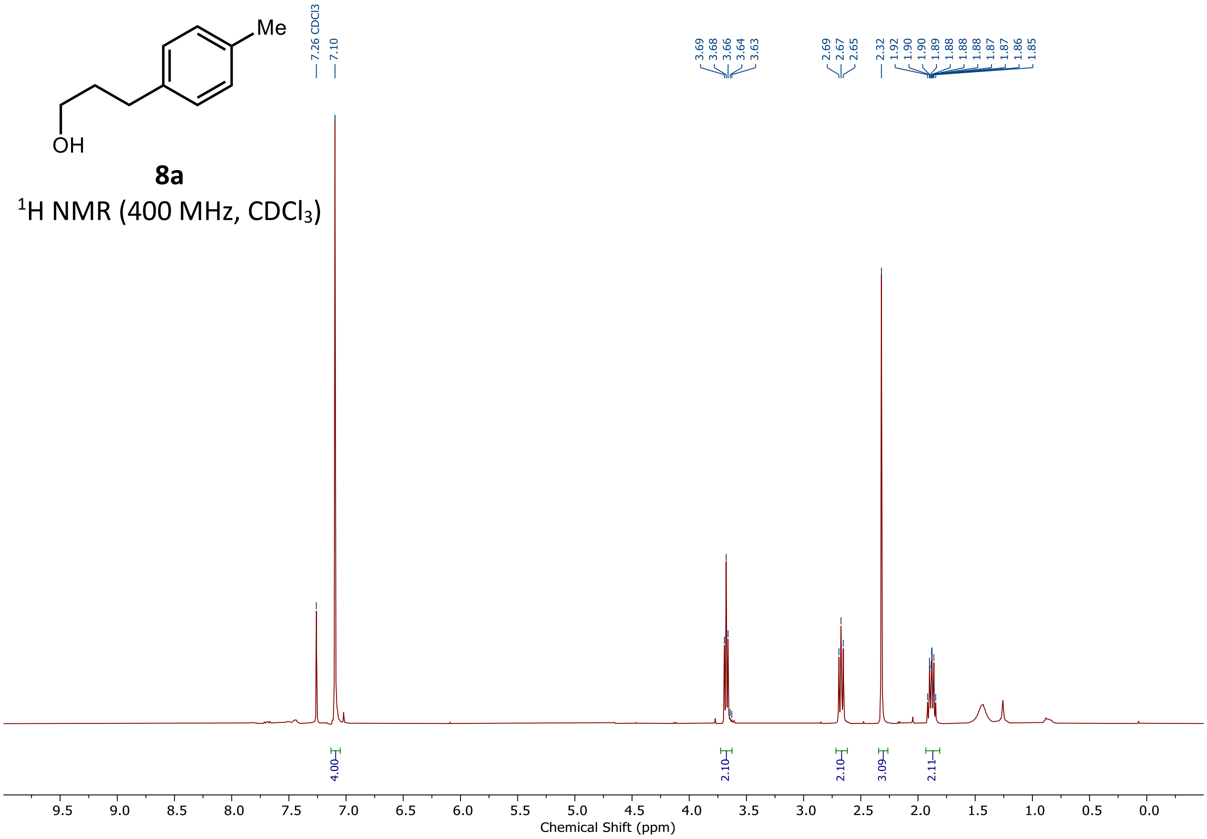
Me
OH
8a
1H NMR (400 MHz, CDCl3)
7.26 CDCl3
7.10
3.69
3.68
3.66
3.64
3.63
2.69
2.67
2.65
2.32
1.92
1.90
1.90
1.89
1.88
1.88
1.88
1.87
1.87
1.86
1.85
4.00
2.10
2.10
3.09
2.11
9.5 9.0 8.5 8.0 7.5 7.0 6.5 6.0 5.5 5.0 4.5 4.0 3.5 3.0 2.5 2.0 1.5 1.0 0.5 0.0
Chemical Shift (ppm)

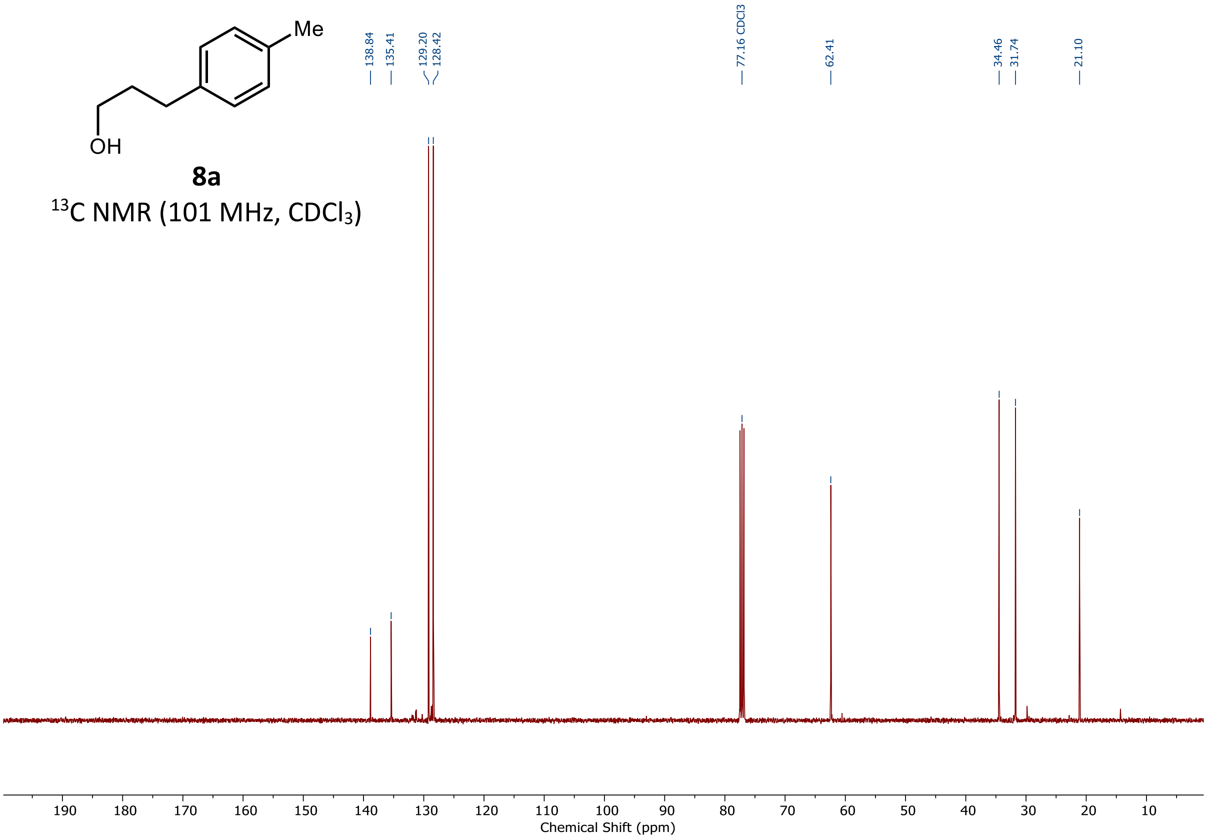
Me
OH
8a
13C NMR (101 MHz, CDCl3)
138.84
135.41
129.20
128.42
77.16 CDCl3
62.41
34.46
31.74
21.10
190 180 170 160 150 140 130 120 110 100 90 80 70 60 50 40 30 20 10
Chemical Shift (ppm)

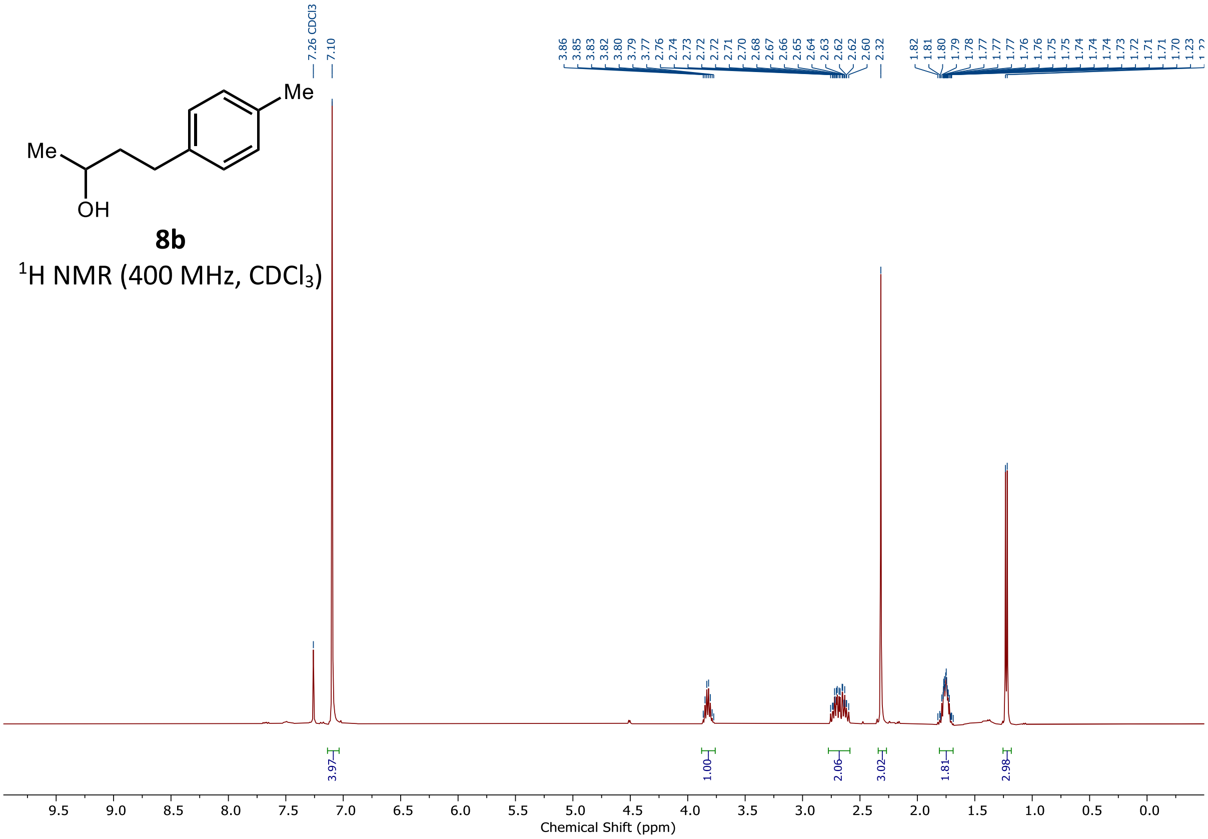
Me
Me
OH
8b
$^{1}$H NMR (400 MHz, CDCl$_3$)
7.26 CDCl3
7.10
3.86
3.85
3.83
3.82
3.80
3.79
3.77
2.76
2.74
2.73
2.72
2.72
2.71
2.70
2.68
2.67
2.66
2.65
2.64
2.63
2.62
2.62
2.60
2.32
1.82
1.81
1.80
1.79
1.78
1.77
1.77
1.76
1.76
1.75
1.74
1.74
1.73
1.72
1.71
1.70
1.23
3.97
1.00
2.06
3.02
1.81
2.98
9.5
9.0
8.5
8.0
7.5
7.0
6.5
6.0
5.5
5.0
4.5
4.0
3.5
3.0
2.5
2.0
1.5
1.0
0.5
0.0
Chemical Shift (ppm)

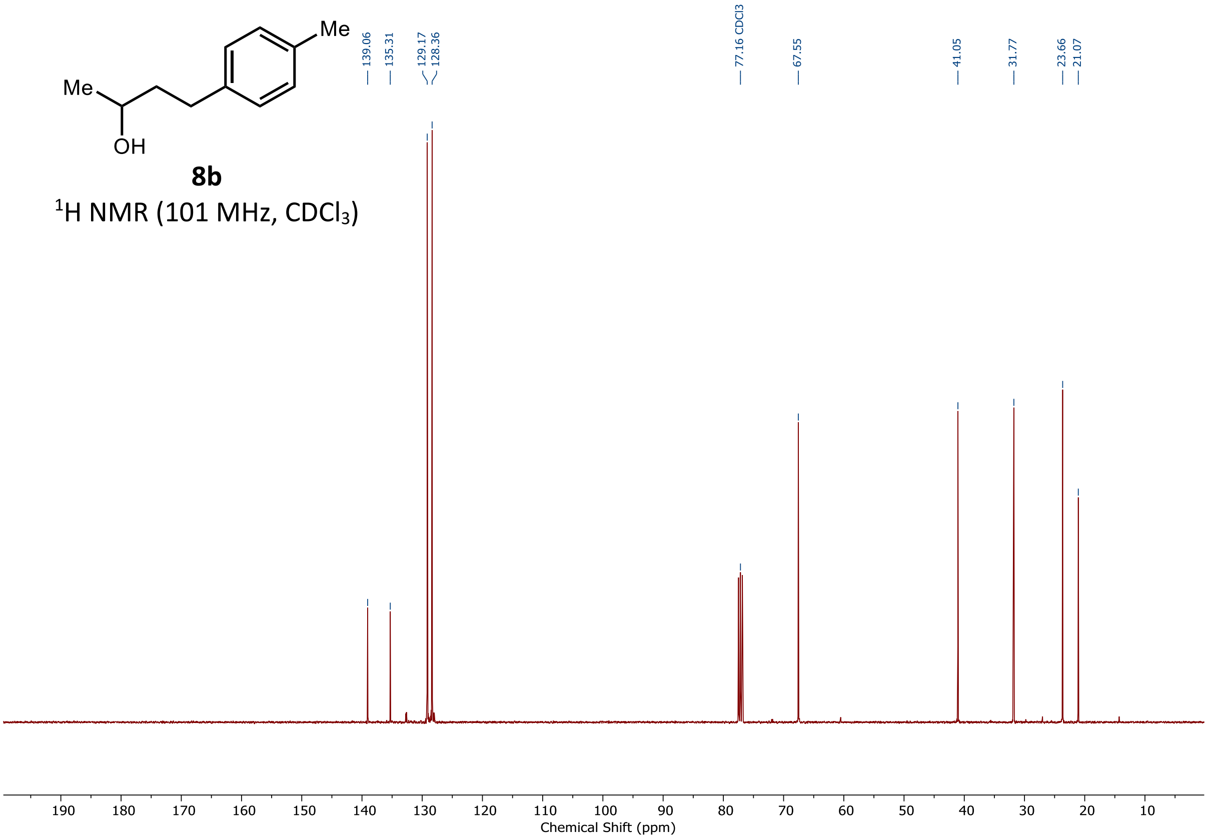
Me
Me
OH
8b
$^{1}$H NMR (101 MHz, CDCl$_3$)
139.06
135.31
129.17
128.36
77.16 CDCl3
67.55
41.05
31.77
23.66
21.07
190
180
170
160
150
140
130
120
110
100
90
80
70
60
50
40
30
20
10
Chemical Shift (ppm)

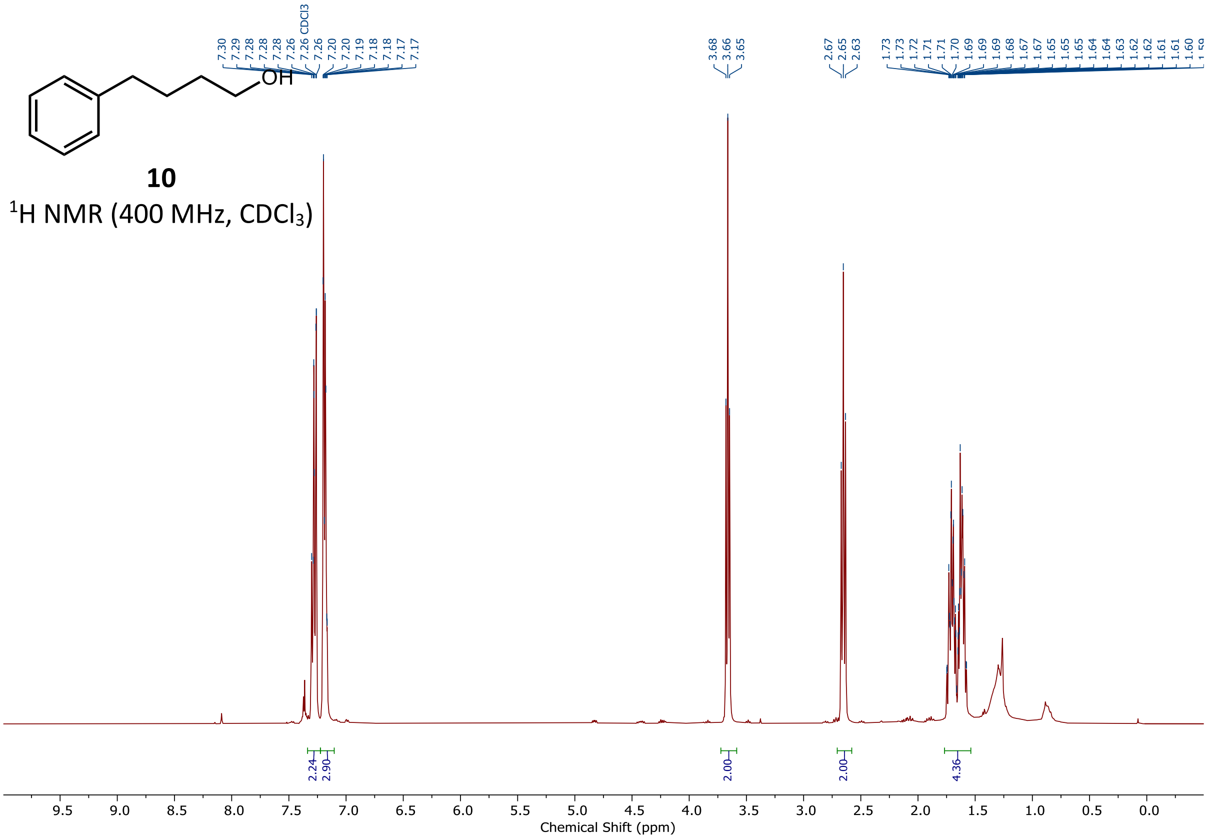
7.30 7.29 7.28 7.28 7.28 7.26 7.26 CDCl3 7.26 7.20 7.20 7.19 7.18 7.18 7.17 7.17
3.68 3.66 3.65
2.67 2.65 2.63
1.73 1.73 1.72 1.71 1.71 1.70 1.69 1.69 1.69 1.68 1.67 1.67 1.65 1.65 1.65 1.64 1.64 1.63 1.62 1.62 1.61 1.61 1.60
OH
10
$^{1}$H NMR (400 MHz, $CDCl_3$)
2.24 2.90 2.00 2.00 4.36
9.5 9.0 8.5 8.0 7.5 7.0 6.5 6.0 5.5 5.0 4.5 4.0 3.5 3.0 2.5 2.0 1.5 1.0 0.5 0.0
Chemical Shift (ppm)

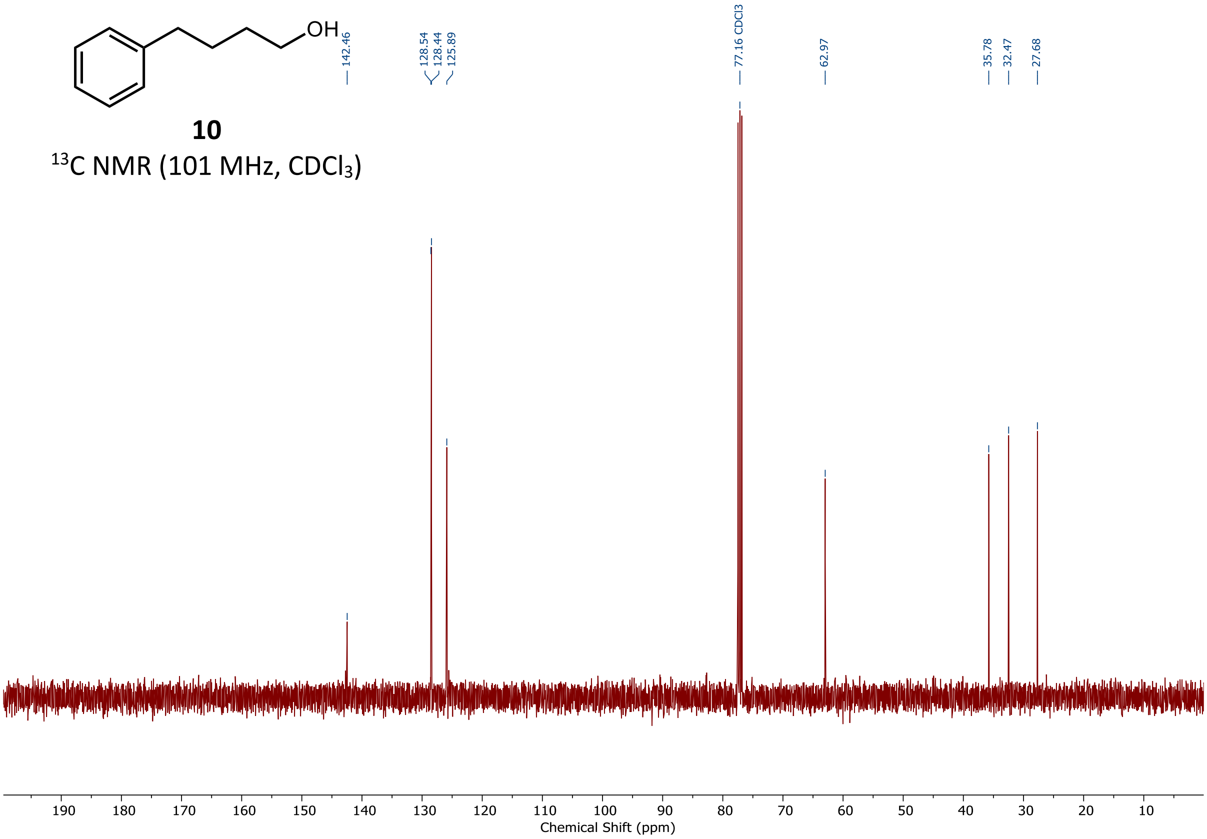
OH
10
$^{13}$C NMR (101 MHz, $CDCl_3$)
142.46
128.54 128.44 125.89
77.16 CDCl3
62.97
35.78 32.47 27.68
190 180 170 160 150 140 130 120 110 100 90 80 70 60 50 40 30 20 10
Chemical Shift (ppm)

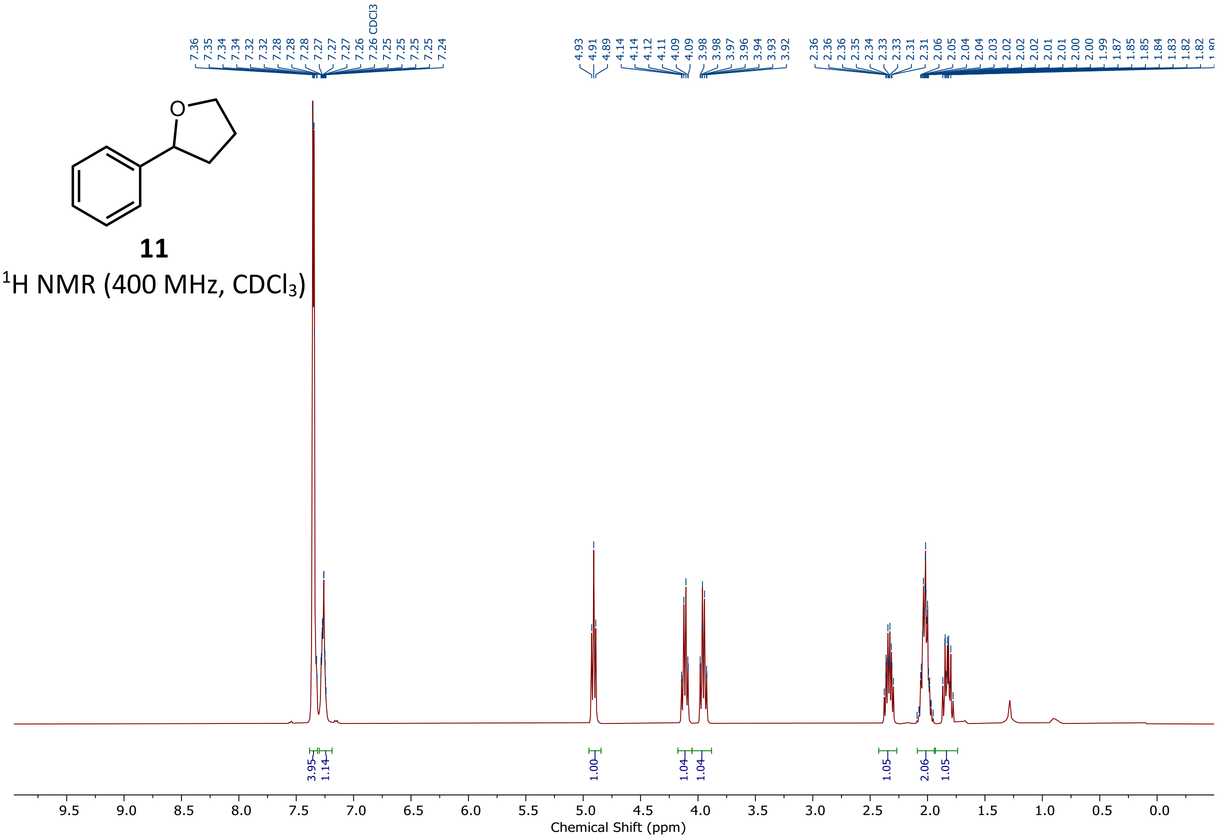


**11**

$^{1}H$ NMR (400 MHz, $CDCl_3$)

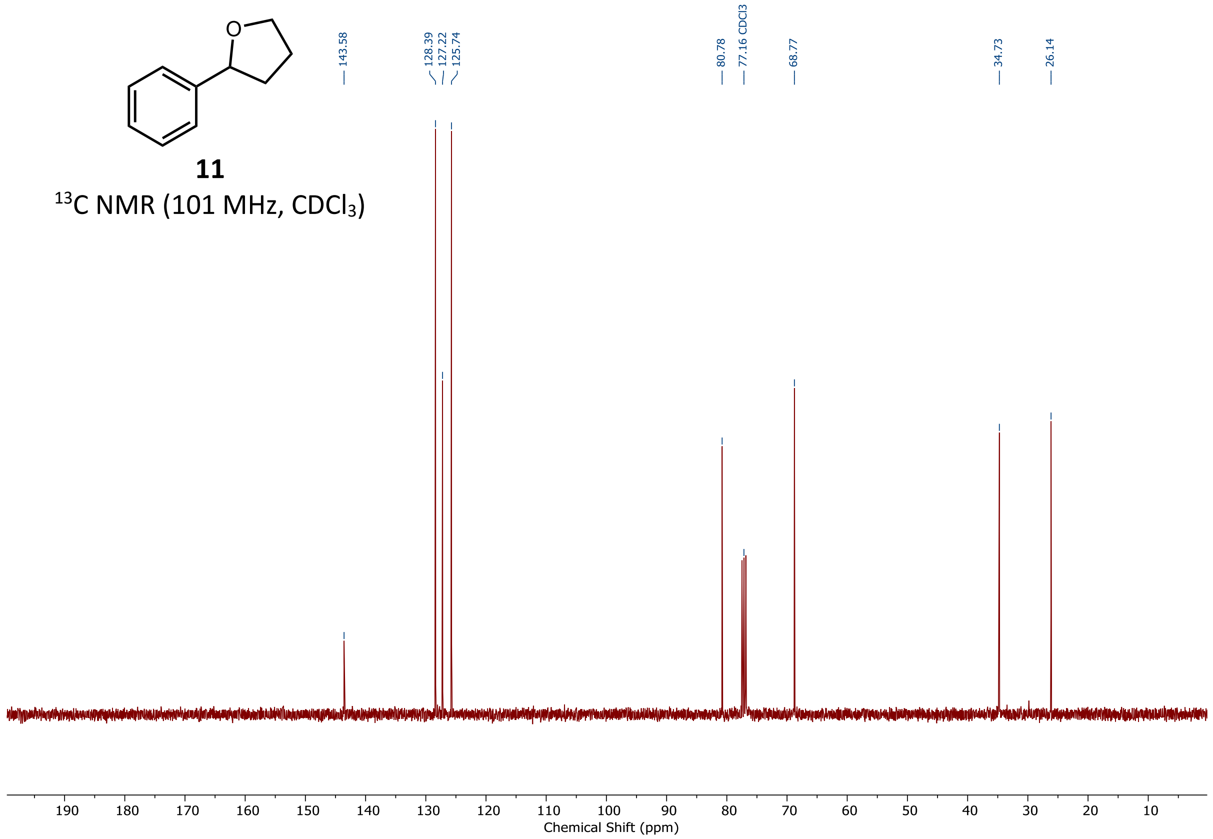


**11**

$^{13}C$ NMR (101 MHz, $CDCl_3$)

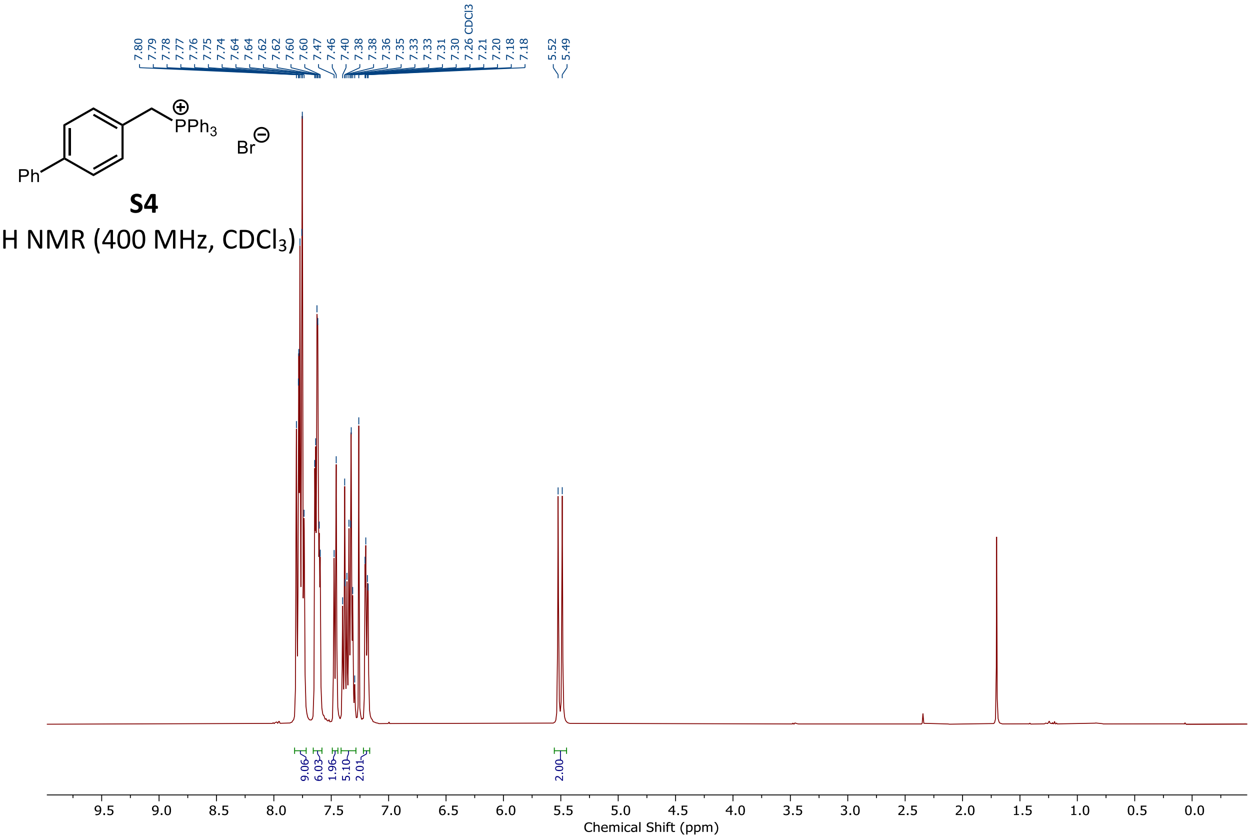


**S4**

$^{1}H$ NMR (400 MHz, $CDCl_3$)

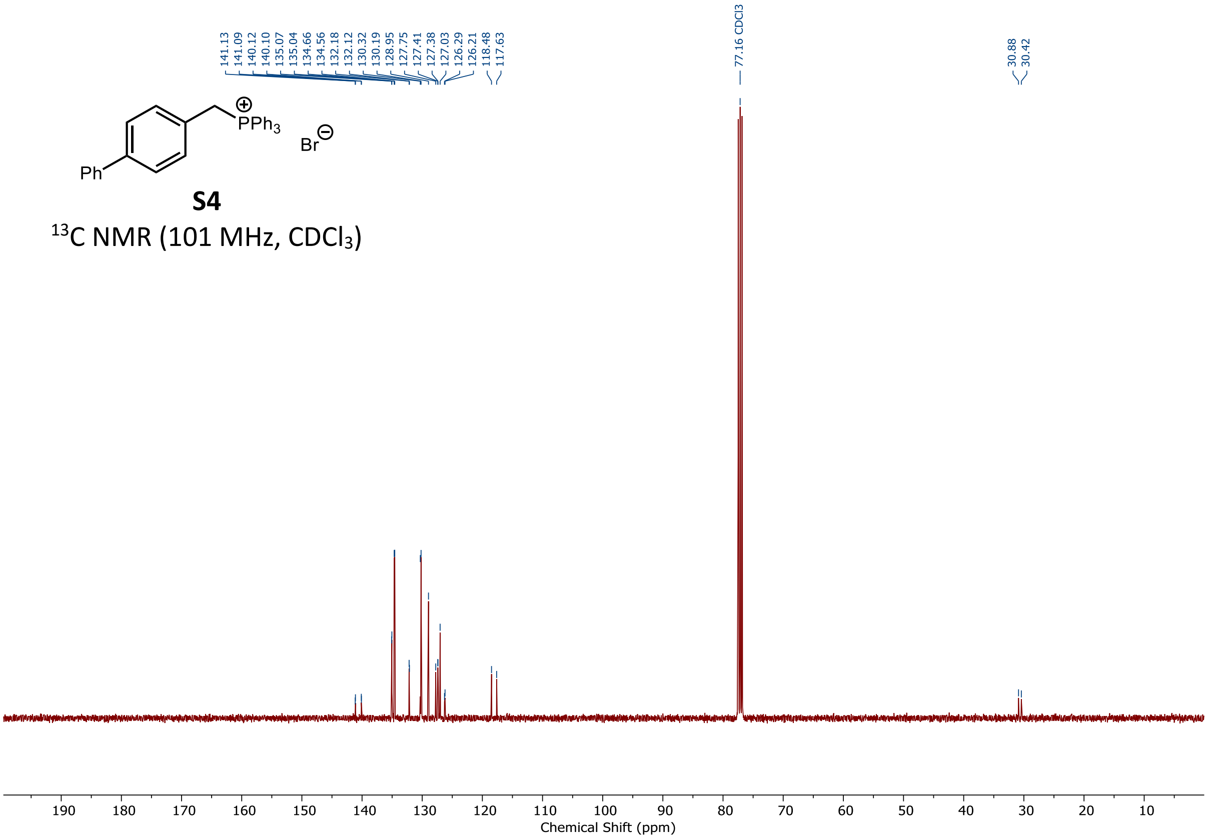


**S4**

$^{13}C$ NMR (101 MHz, $CDCl_3$)

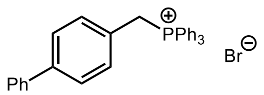


**S4**

$^{31}P$ NMR (162 MHz, $CDCl_3$)

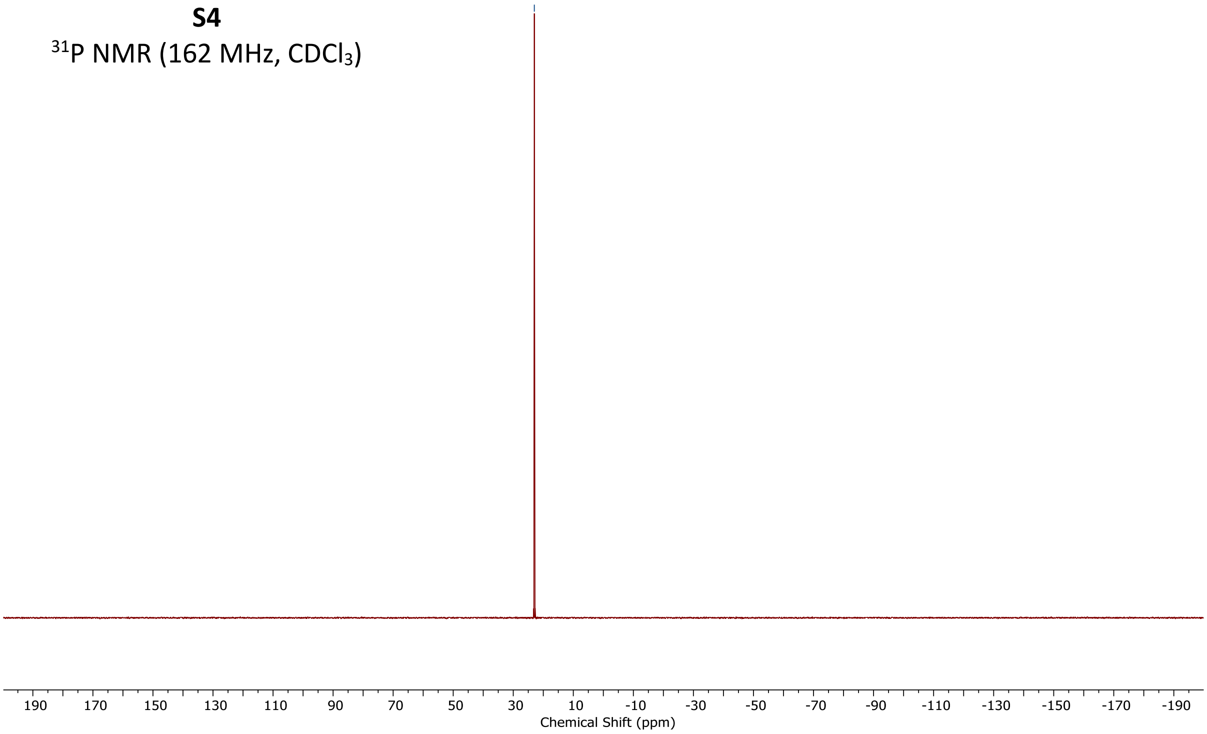

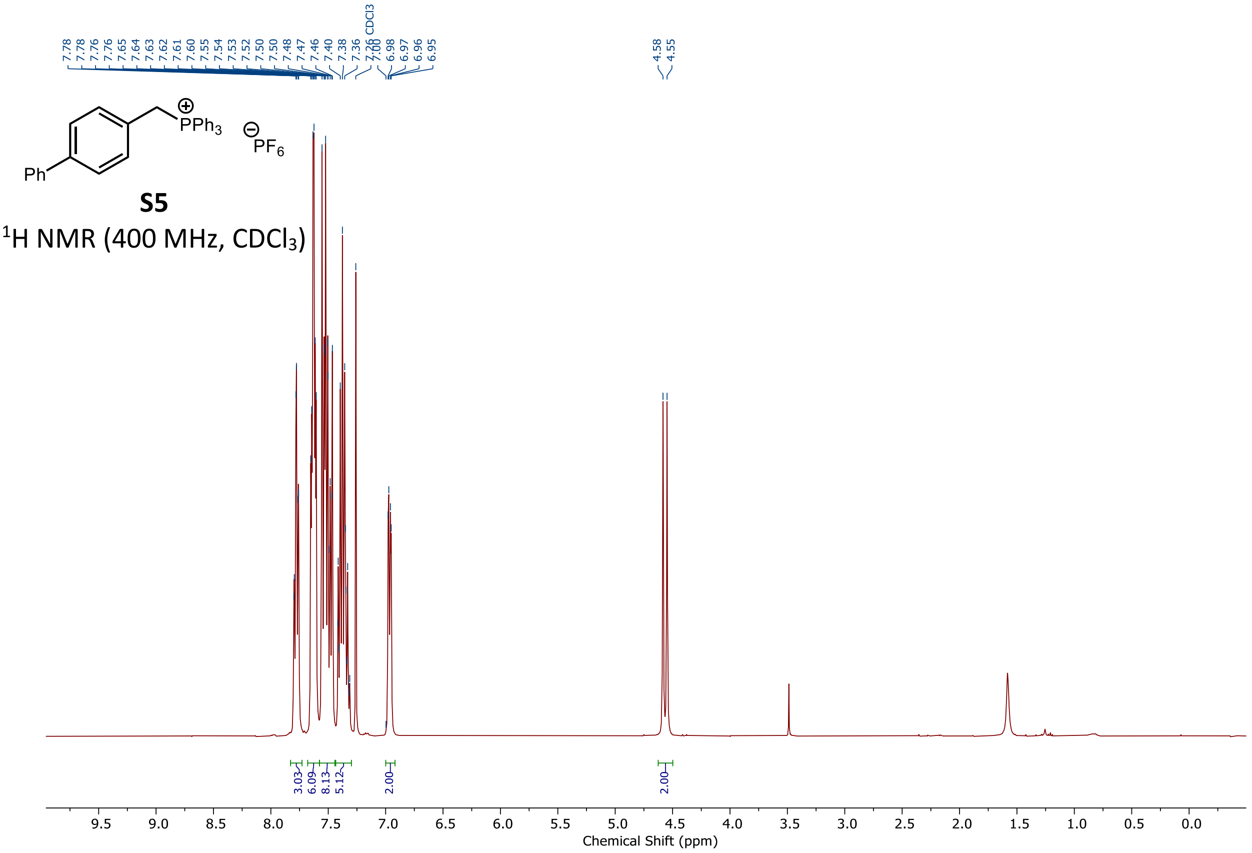

PPh3
PF6
Ph
S5
1H NMR (400 MHz, CDCl3)
Chemical Shift (ppm)


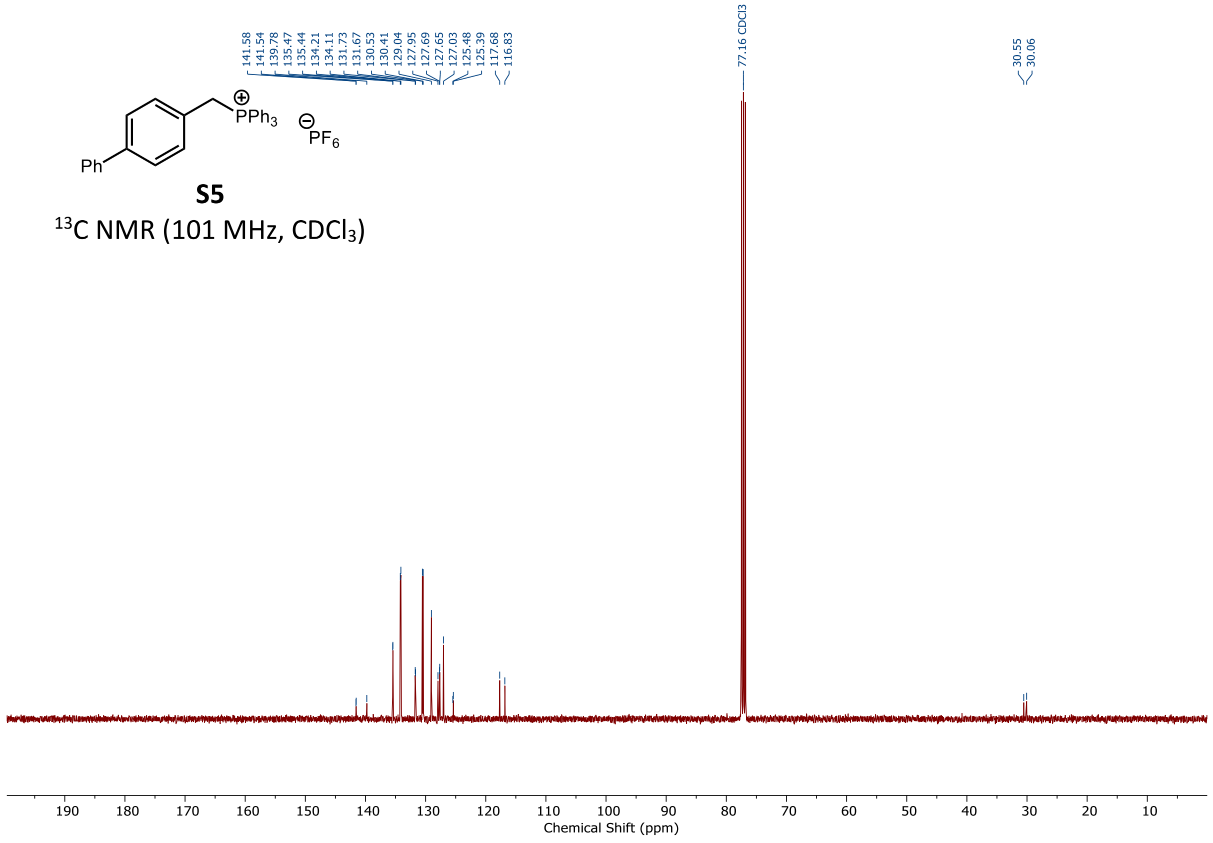

PPh3
PF6
Ph
S5
13C NMR (101 MHz, CDCl3)
Chemical Shift (ppm)

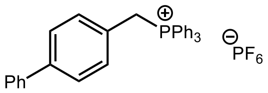


**S5**

$^{31}$P NMR (162 MHz, $CDCl_3$)

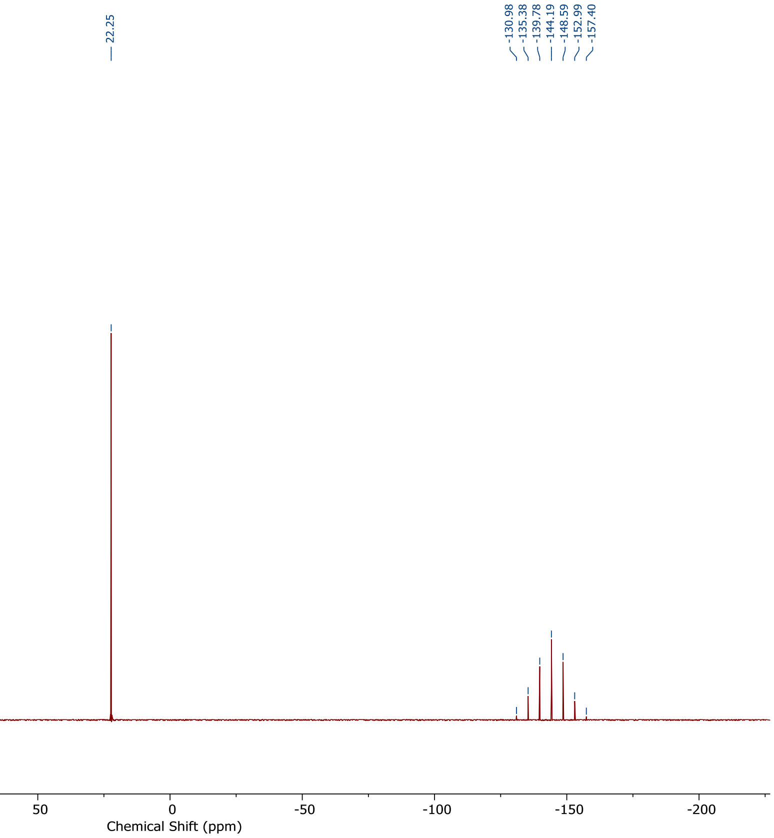


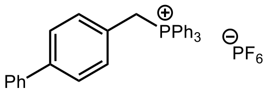


**S5**

$^{19}$F NMR (376 MHz, $CDCl_3$)

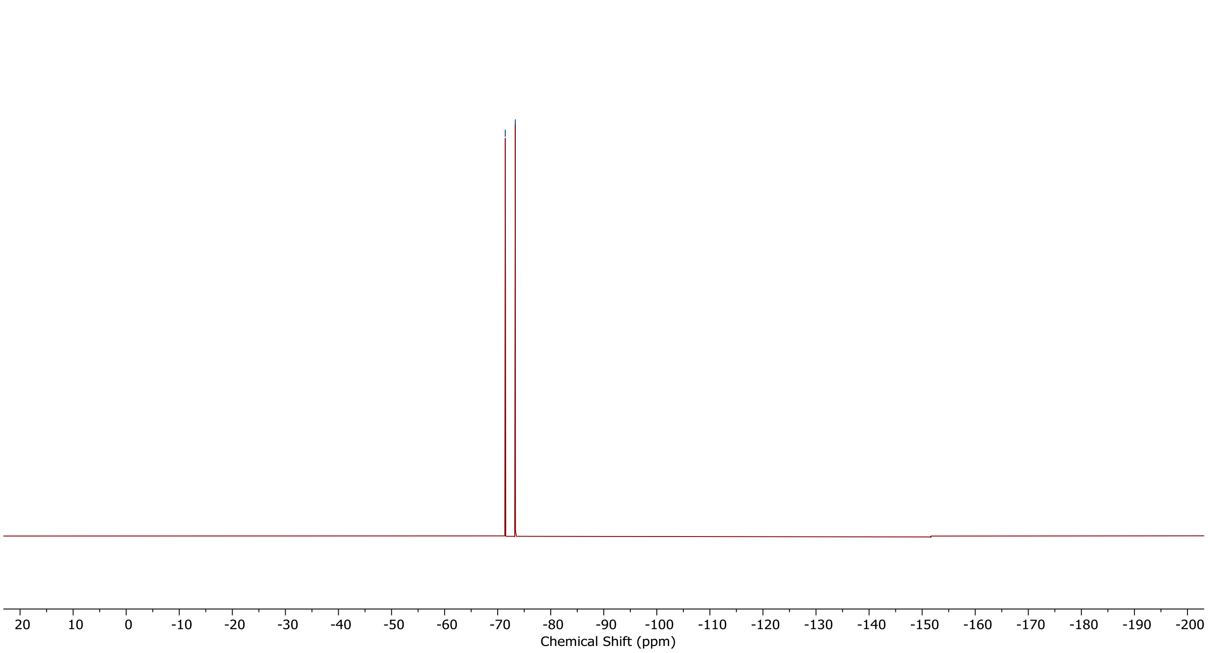

# 14. GC–MS Chromatograms of Reaction Mixtures

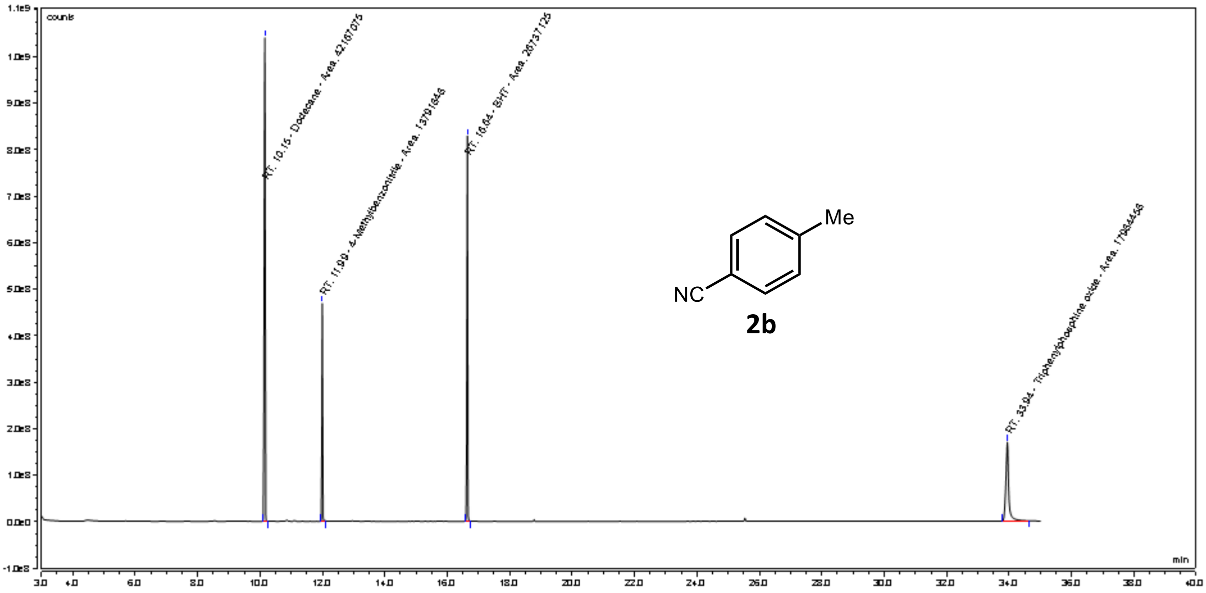


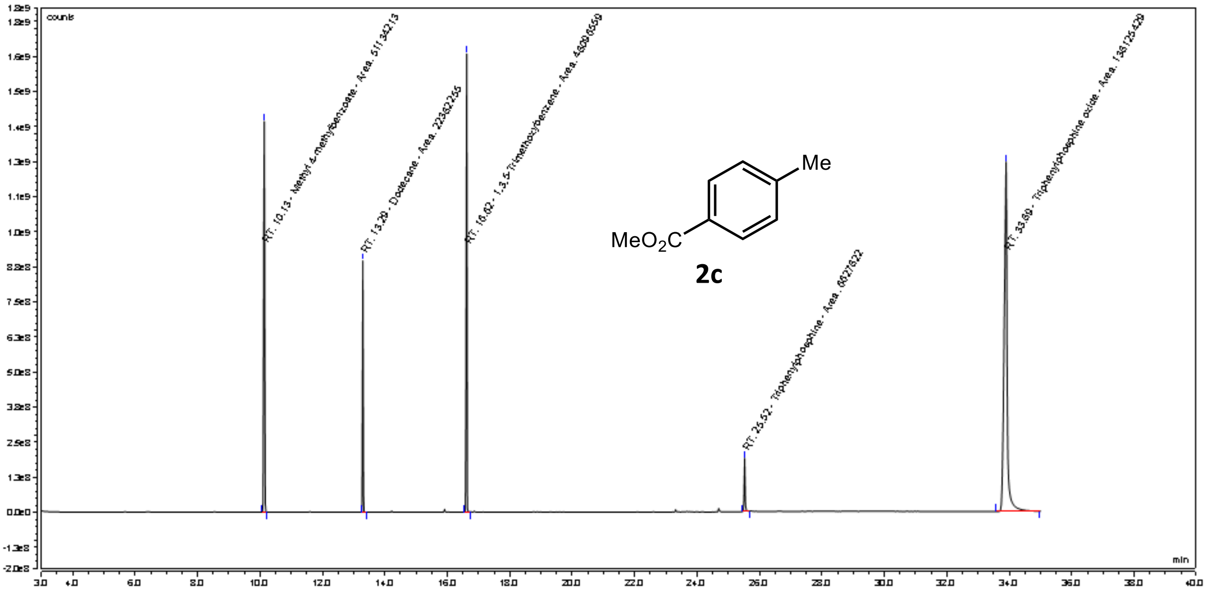

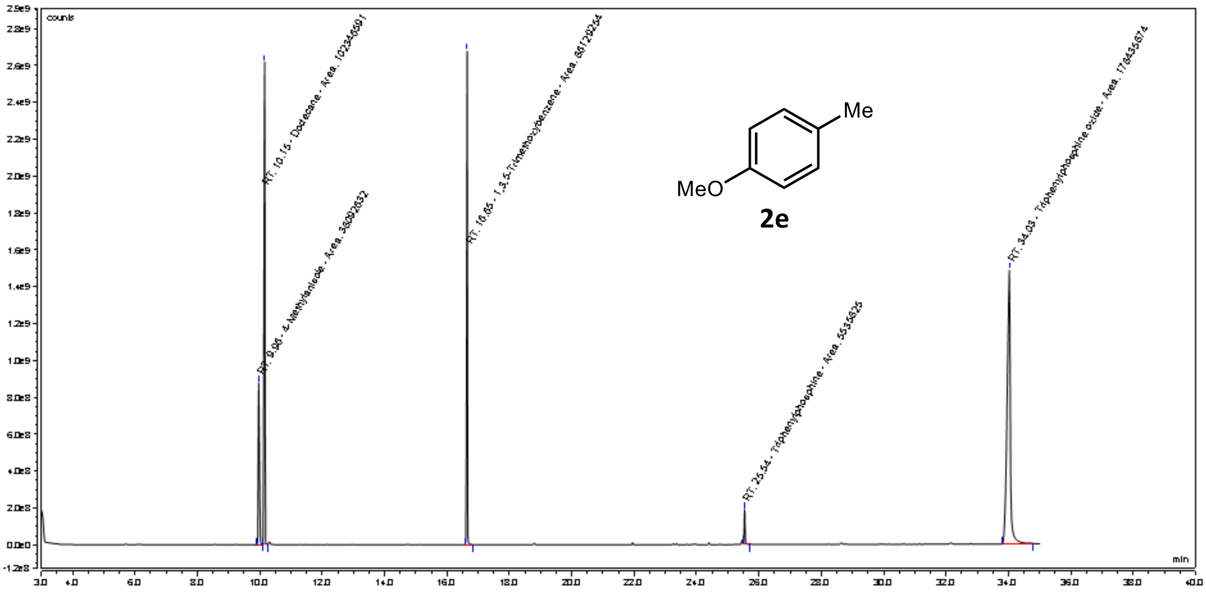


**2g** prepared from alcohol 4-*tert*-butylbenzyl alcohol:

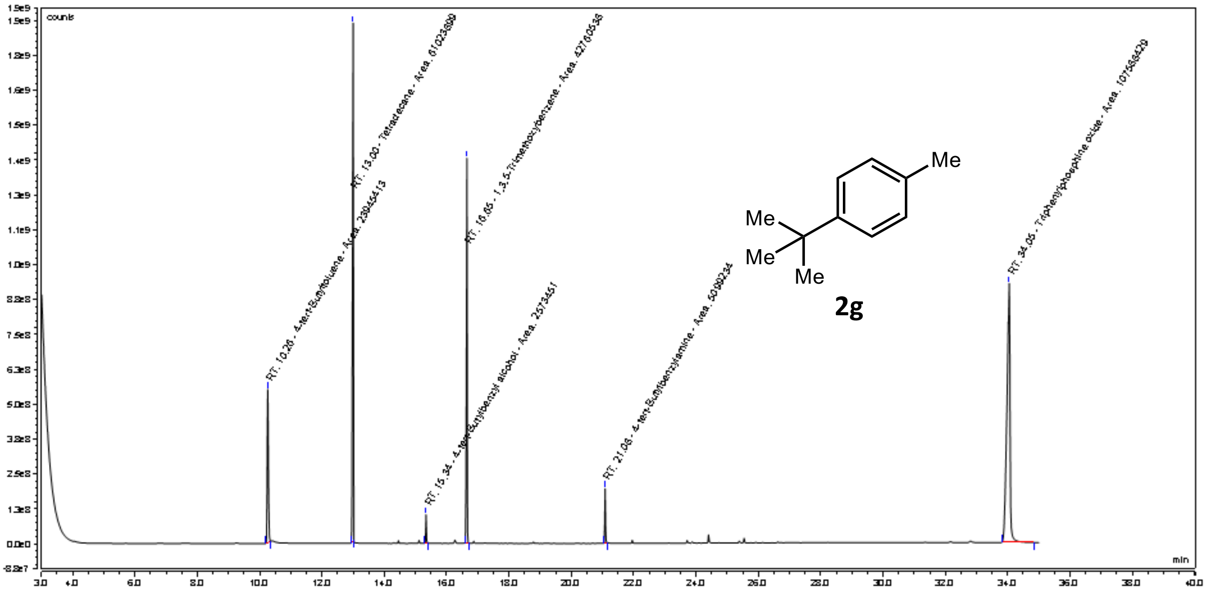

**2g** prepared from 4-*tert*-butylbenzyl mercaptan:

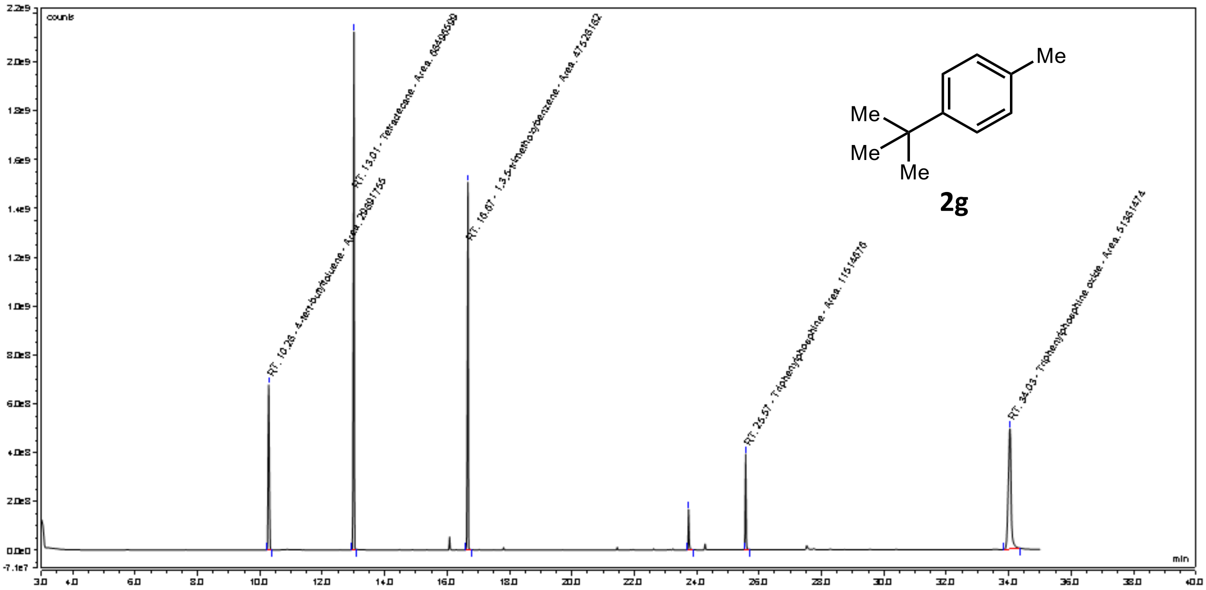


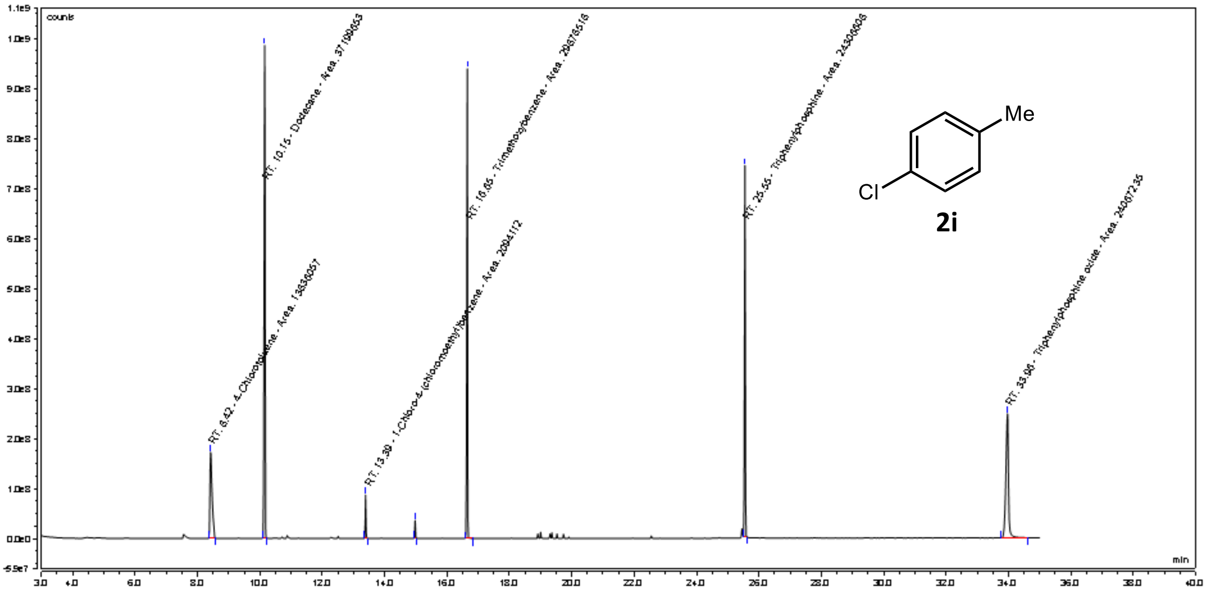

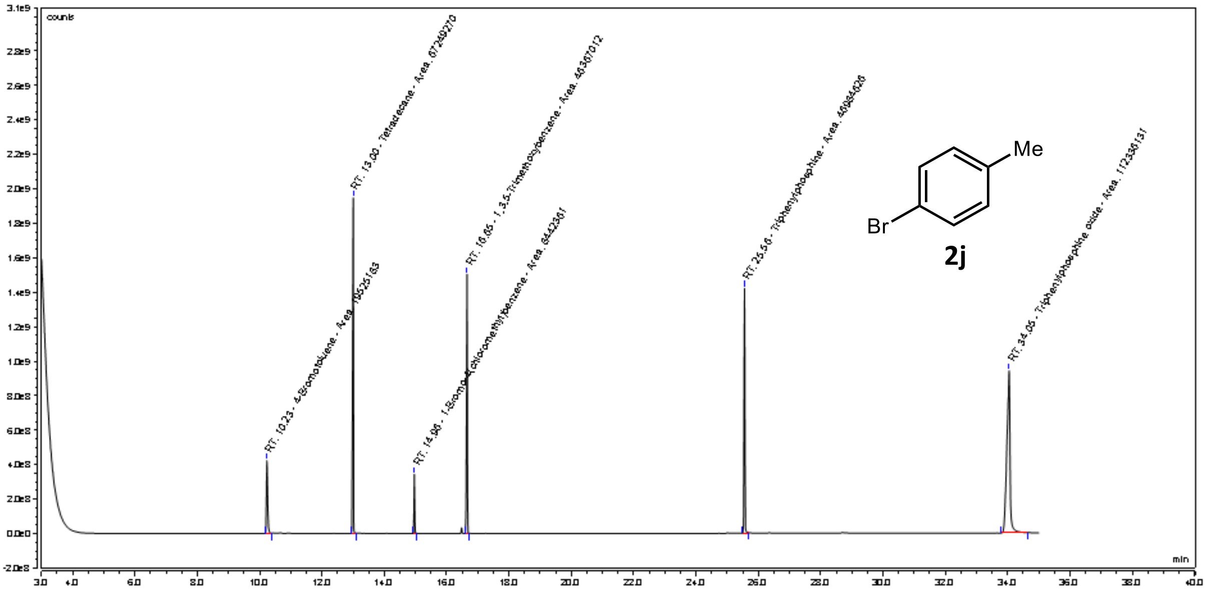

counts
RT: 10.23 - 4-Bromotoluene - Area: 19525163
RT: 13.00 - Tetradecane - Area: 67269270
RT: 14.96 - 1-Bromo-4-(chloromethyl)benzene - Area: 6442361
RT: 16.65 - 1,3,5-Trimethoxybenzene - Area: 48367012
RT: 25.56 - Triphenylphosphine - Area: 40964626
RT: 34.05 - Triphenylphosphine oxide - Area: 112336131
Me
Br
2j
min


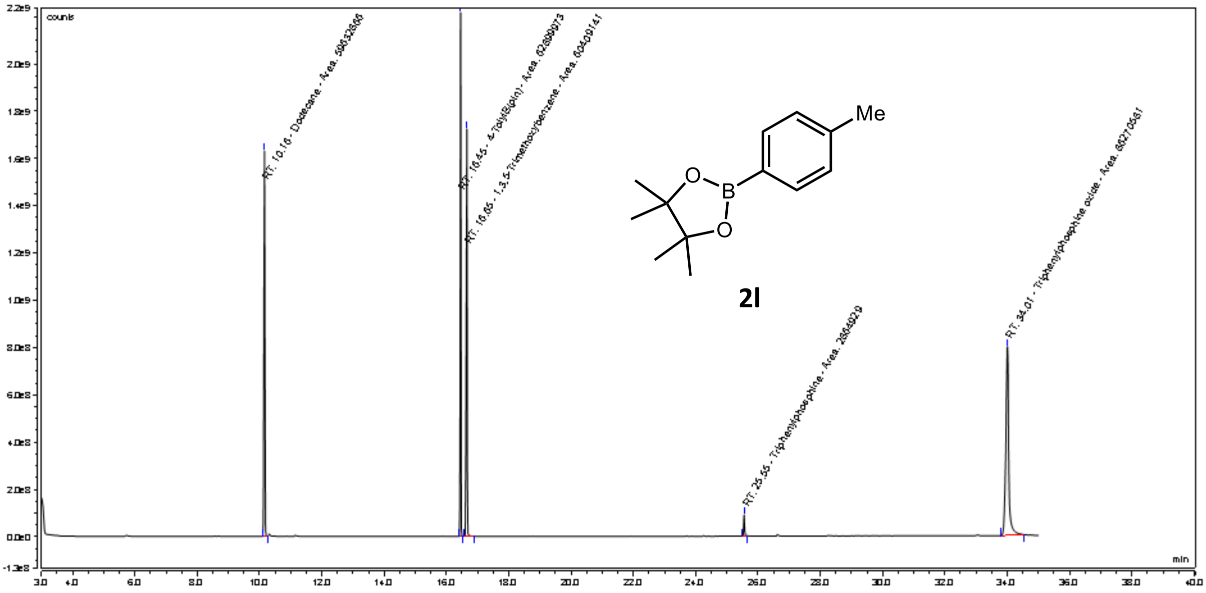

counts
RT: 10.16 - Dodecane - Area: 59632666
RT: 16.45 - 4-TolylB(pin) - Area: 62809973
RT: 16.65 - 1,3,5-Trimethoxybenzene - Area: 60409141
RT: 25.55 - Triphenylphosphine - Area: 2864929
RT: 34.01 - Triphenylphosphine oxide - Area: 86270561
Me
O
B
O
2l
min

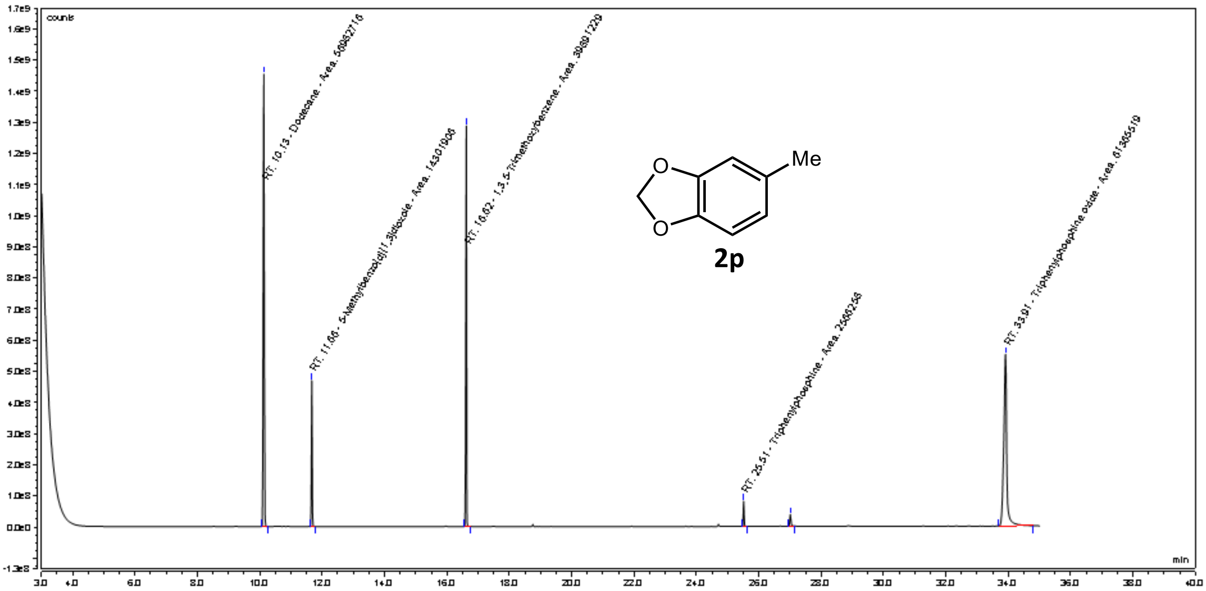
Me
O
O
2p

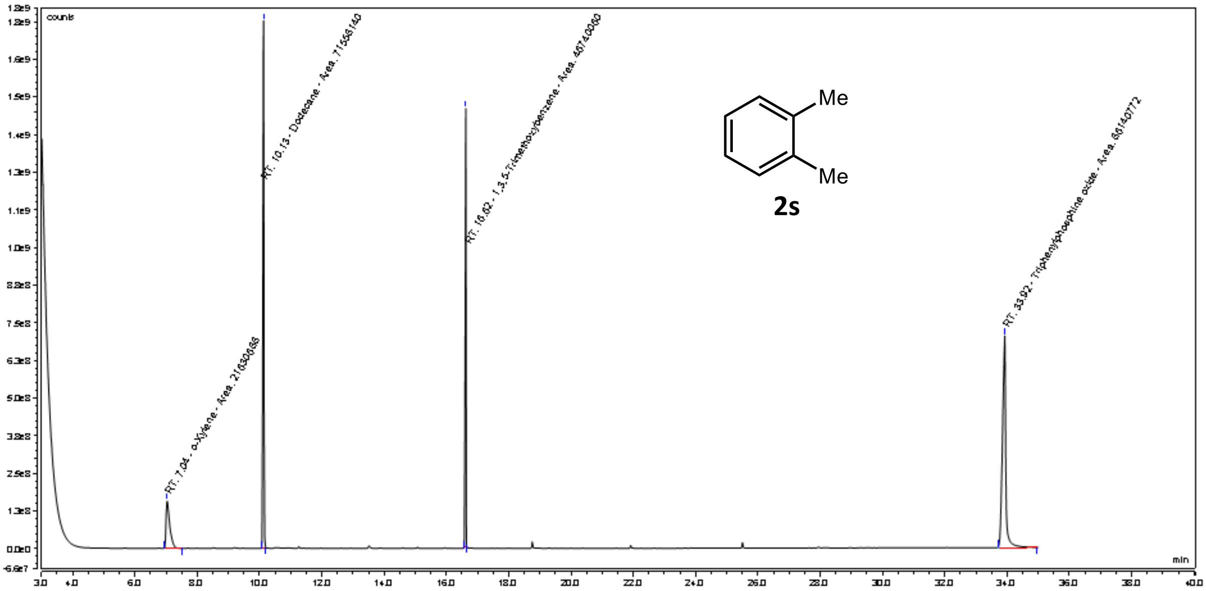
Me
Me
2s

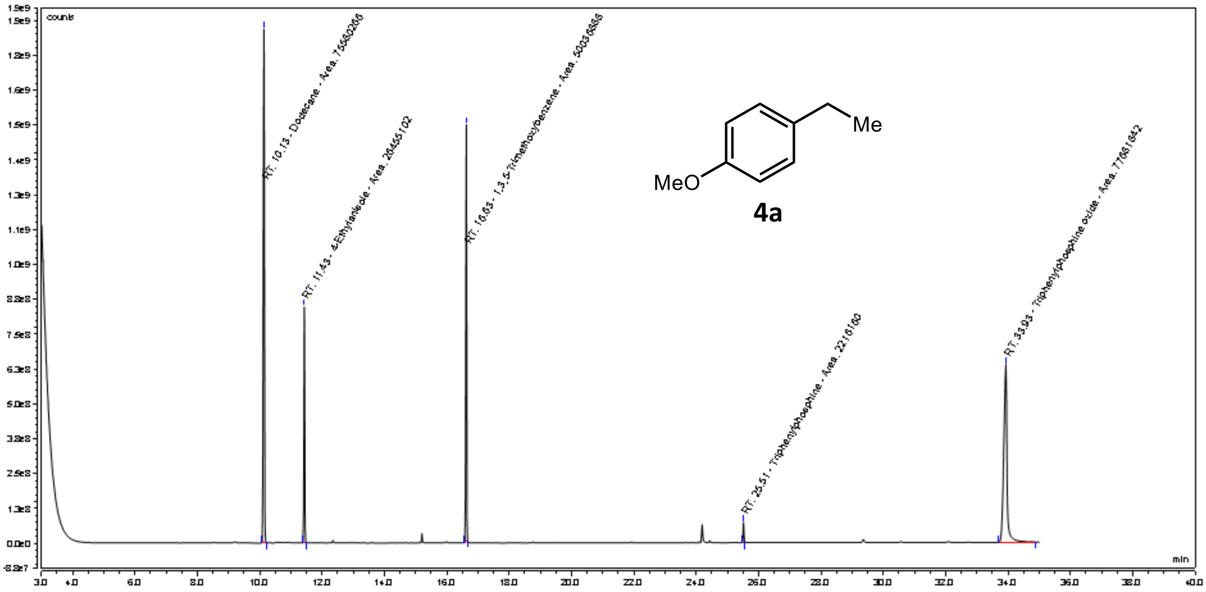
counts
RT: 10.13 - Dodecane - Area: 75580266
RT: 11.43 - 4-Ethylanisole - Area: 28455102
RT: 16.63 - 1,3,5-Trimethoxybenzene - Area: 50036656
RT: 25.51 - Triphenylphosphine - Area: 2216160
RT: 33.93 - Triphenylphosphine oxide - Area: 77681842
MeO
Me
4a
min

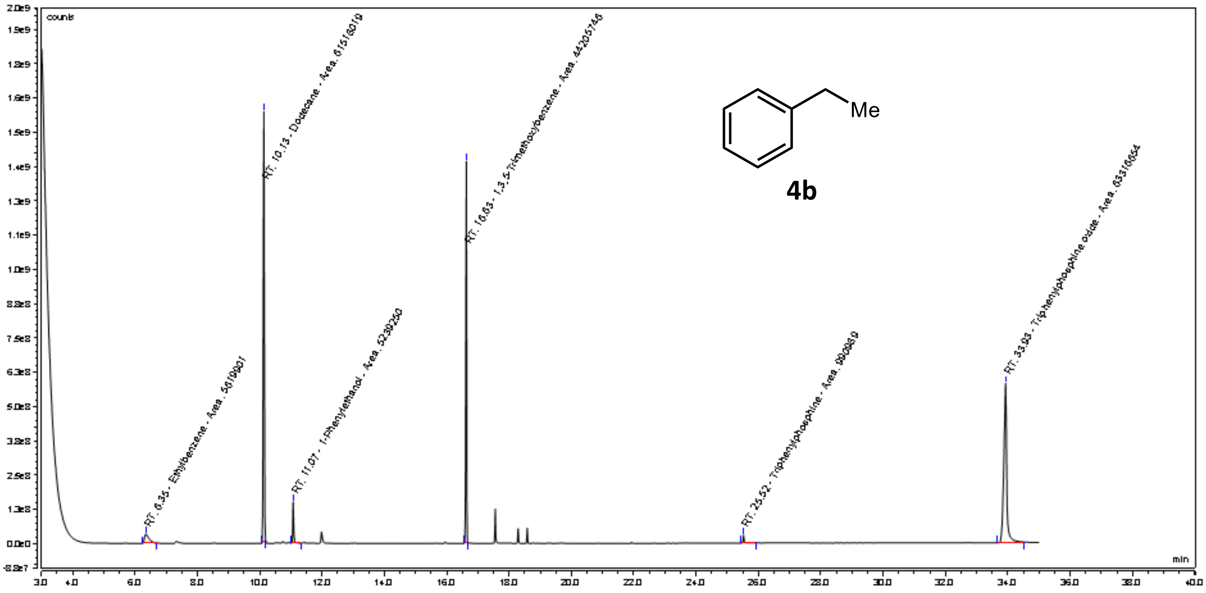
counts
RT: 6.35 - Ethylbenzene - Area: 5619901
RT: 10.13 - Dodecane - Area: 61516019
RT: 11.07 - 1-Phenylethanol - Area: 5239250
RT: 16.63 - 1,3,5-Trimethoxybenzene - Area: 44205746
RT: 25.52 - Triphenylphosphine - Area: 900969
RT: 33.93 - Triphenylphosphine oxide - Area: 63316654
Me
4b
min

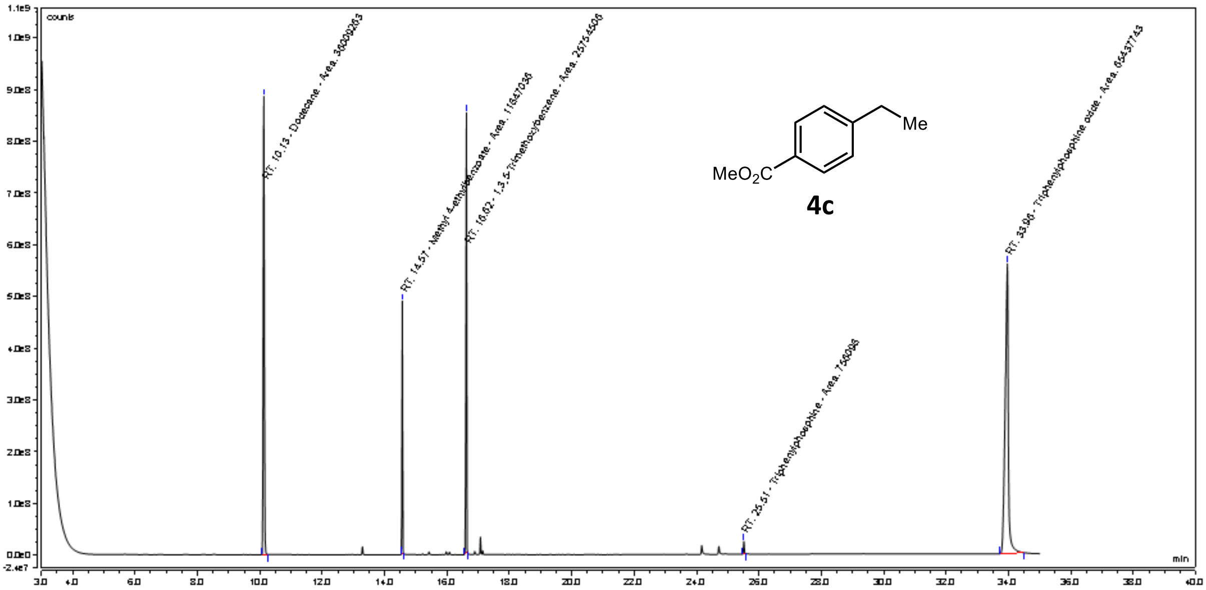
counts
RT: 10.13 - Dodecane - Area: 36009263
RT: 14.57 - Methyl 4-ethylbenzoate - Area: 11847036
RT: 16.62 - 1,3,5-Trimethoxybenzene - Area: 25754506
RT: 25.51 - Triphenylphosphine - Area: 756096
RT: 33.96 - Triphenylphosphine oxide - Area: 65437743
MeO2C
Me
4c
min

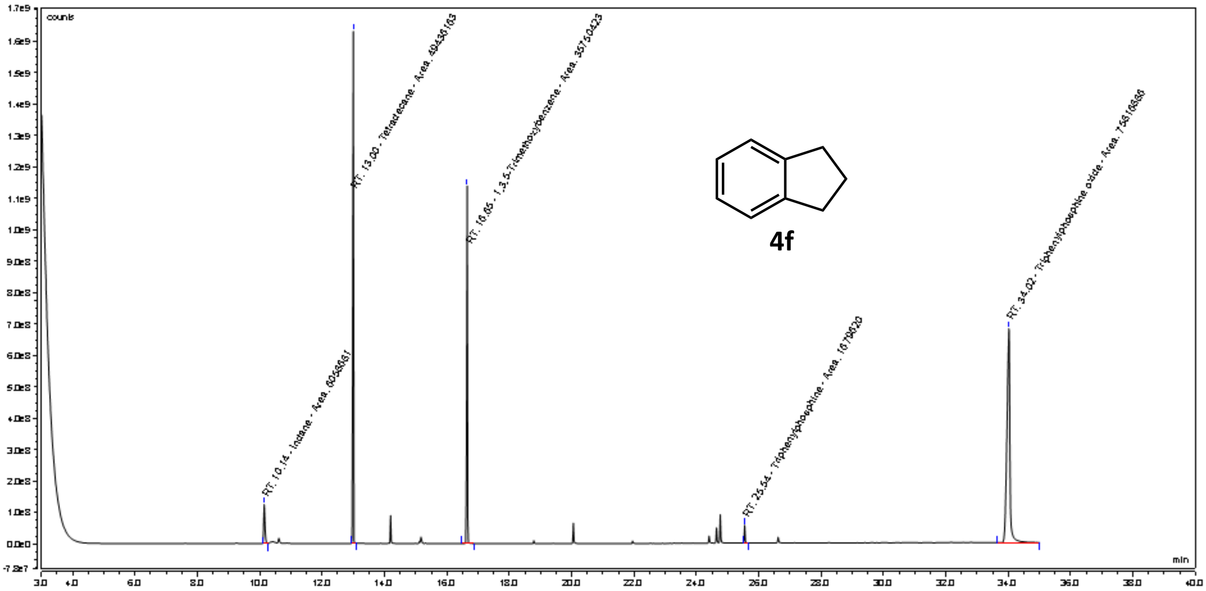
counts
RT: 10.14 - Indane - Area: 6058681
RT: 13.00 - Tetradecane - Area: 49436163
RT: 16.65 - 1,3,5-Trimethoxybenzene - Area: 35750423
RT: 25.54 - Triphenylphosphine - Area: 1679820
RT: 34.02 - Triphenylphosphine oxide - Area: 75816866
4f
min

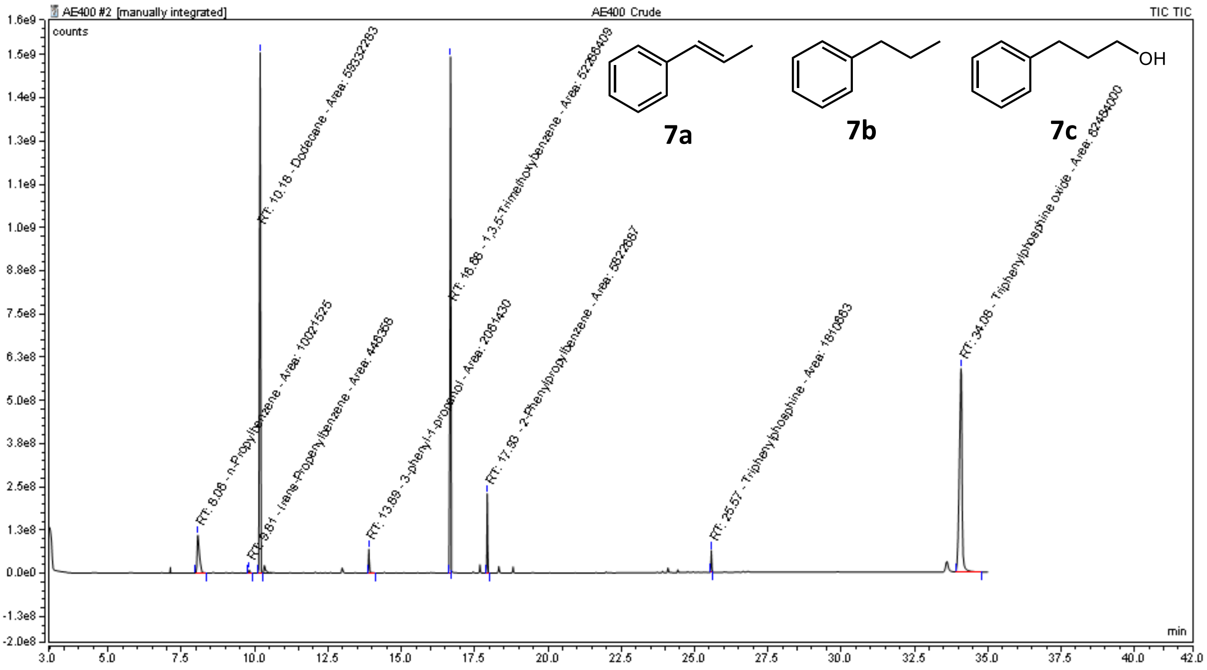
AE400 #2 [manually integrated]
AE400 Crude
TIC TIC
counts
RT: 8.06 - n-Propylbenzene - Area: 10021525
RT: 10.16 - Dodecane - Area: 59332263
RT: 13.89 - 3-phenyl-1-propanol - Area: 2081430
RT: 17.93 - 2-Phenylpropylbenzene
RT: 25.57 - Triphenylphosphine
RT: 34.09 - Triphenylphosphine oxide
7a
7b
OH
7c
min
